# The Incidence and Risk Factors for the Development of Fractures in Military Populations: A Systematic Review

**DOI:** 10.3390/healthcare14101322

**Published:** 2026-05-13

**Authors:** Patrick G. Campbell, Rodney Pope, Vinicius Simas, Elisa F. D. Canetti, Benjamin Schram, Robin M. Orr

**Affiliations:** 1 School of Behavioural and Health Sciences, Australian Catholic University, Banyo, QLD 4014, Australia; 2 Sports Performance, Recovery, Injury and New Technologies (SPRINT) Research Centre, Australian Catholic University, Brisbane, QLD 4014, Australia; 3 National Centre for Veterans and Families, Australian Catholic University, Banyo, QLD 4014, Australia; 4 Tactical Research Unit, Bond University, Robina, QLD 4226, Australia; 5 School of Allied Health, Exercise and Sports Sciences, Charles Sturt University, Albury, NSW 2640, Australia; 6 Faculty of Health Science and Medicine, Bond University, Robina, QLD 4226, Australia

**Keywords:** military recruits, prevalence, risk, armed forces personnel, injury

## Abstract

**Objectives:** Traumatic fractures represent a considerable burden to military personnel across nations. Despite substantial research examining traumatic fracture incidence and risk factors, there is no comprehensive synthesis of evidence on traumatic bone fractures in military populations. This study aimed to identify and synthesise findings from studies reporting on the incidence of, and risk factors for, traumatic fractures in military personnel. **Design:** Systematic review. **Methods:** A structured search was performed in PubMed, EBSCO, CINAHL, and ProQuest using key terms related to fractures, occupational exposure, and risk. Eligible studies were screened, and key findings including risk factors, incidence rates, and effect sizes were systematically extracted and summarised. **Results:** Twenty-nine studies were included, with four studies reporting on recruits/trainees and 25 on qualified personnel. Recruit incidence ranged from 21.8 to 105.3 fractures per 1000 person-years, while the incidence in qualified personnel ranged from 1.9 to 57.6 fractures per 1000 person-years. Enlisted personnel, younger servicemembers (18–29 years), and personnel of the Army and Marine Corps branches were at increased risk. Traumatic fractures predominantly occurred in the lower extremities. Risk factors and mechanisms for traumatic fractures in military personnel included sports participation and physical training. Other common mechanisms included motor vehicle accidents and collisions, and (in combat settings) blasts from improvised explosive devices and gunshot wounds. **Conclusions:** Traumatic fractures are a substantial source of injury across military careers, not limited to initial training. Army and Marine Corps personnel face higher fracture risks, particularly in the lower limbs, with sports/physical training and combat-related risk factors associated with increased susceptibility.

## 1. Introduction

Traumatic fractures of skeletal bone are characterised by breaks or ruptures in the bone, and are investigated and diagnosed using clinical examinations, plain radiographs, magnetic resonance imaging (MRI), computed tomography (CT) scans and other forms of medical imaging (e.g., bone scan) [1]. In civilian populations, traumatic fractures represent a substantial public health concern. For example, the overall incidence of traumatic fractures in the United Kingdom was calculated to be 36 cases per 1000 people per year, with accompanying public health costs in excess of £700 million [1]. Similarly, within a Dutch population the total incidence of non-vertebral traumatic fractures was reported to be 25.4 (95% CI, 23.0–28.0) cases per 1000 person-years [2]. In this Dutch study, the most common sites of traumatic bone fracture were the hip, vertebrae, wrist, hand, and pelvis, with incidence rates for traumatic fractures at these sites ranging from 1.24 to 5.37 cases per 1000 person-years [2]. Traumatic fractures also pose a significant burden within military populations. Traumatic fractures have been calculated to comprise ~10% of lower limb injuries within the New Zealand Defence Force, representing a considerable injury burden for military organisations and frequently leading to recruit discharge [3]. The impacts of these injuries extend beyond individual health outcomes, influencing operational effectiveness, medical discharge rates among recruits and qualified personnel, and associated economic costs [1,4,5,6].

Traumatic fractures within civilian populations are often associated with bone diseases like osteoporosis in aged populations or occur within younger children [1,2]. Conversely, in military populations, traumatic fractures are often directly related to the environmental contexts and occupational tasks of military personnel. For example, traumatic fractures often occur in high-energy trauma events involving military vehicles, combat scenarios, enemy munition fire, battle training, or other occupation-specific tasks, and frequently affect the spine, clavicle, face/head region, femur, tibia, fibula, hands, and/or feet [3]. Additional prevalent risk factors for traumatic fractures in military personnel include falls, slips and trips, and motor vehicle accidents [7]. They can also be caused by enemy munitions and their impacts on the individual, vehicles, vessels, aircraft and equipment, particularly during deployment in active combat settings [8,9,10]. Collectively, these examples illustrate how occupational exposures in military contexts contribute to traumatic fracture risk through both routine training activities and operational environments.

Research on the risk factors for traumatic bone fractures in military populations lacks a comprehensive evidence synthesis. Consequently, while numerous studies have investigated the incidence of and risk factors for traumatic fractures in military populations, there is currently no systematic review synthesising this evidence. Further, existing research has typically examined military recruits or trainees separately from qualified defence personnel, leading to a lack of integrated understanding across different military sub-populations. The aim of this systematic review was therefore to identify and synthesise the existing evidence that reports on the incidence of traumatic fractures within military populations, including recruits and trainees, and qualified personnel. Further, evidence regarding factors associated with the risk of traumatic fracture events occurring or developing in these settings was also synthesised.

## 2. Methods

### 2.1. Study Design

This systematic review was conducted to identify and synthesise findings from published studies investigating the incidence of traumatic fractures and associated occupational tasks and risk factors in military populations. The review was reported in accordance with the Preferred Reporting Items for Systematic Reviews and Meta-Analyses (PRISMA) 2020 guidelines [11].

The protocol for this systematic review was registered with the Open Science Framework (OSF) on 27 July 2020 (https://osf.io/w6e7x/) [12]. The original protocol examined traumatic and stress fractures across multiple occupational groups as part of a grant-funded evidence synthesis for a government department. Owing to the volume and complexity of the evidence, the scope was subsequently restricted to military populations, and the overarching review was divided into two separate systematic reviews addressing traumatic fractures (the focus of the present review) and stress fractures (previously published). The methods used in this review were identical to those reported in the companion stress fracture review [13] and were applied to a distinct outcome domain, and are briefly summarised here. Amendments to the registered protocol are detailed in Supplementary Material S1.

### 2.2. Eligibility Criteria, Information Sources, and Search Terms

A comprehensive search of key databases was conducted in May 2024. The databases searched included PubMed, SPORTDiscus, CINAHL and ProQuest (including Consumer Health Databases, Health & Medical Collection, Military Databases, Nursing & Allied Health Database, and Public Health Database). Search terms were developed around three central themes: ‘fractures,’ ‘work,’ and ‘risk.’ An example search strategy for PubMed is shown in Table 1 (search strategies for all databases are included in Supplementary Material S2).

### 2.3. Inclusion and Exclusion Criteria

The inclusion and exclusion criteria applied during screening and selection processes are summarised in Table 2.

A further exclusion criterion, study reported on stress fractures only, was added post hoc to allow for separation of the findings into two separate reviews—one focused on traumatic fractures (this review) and one focused on stress fractures.

### 2.4. Study Selection, Data Collection Process, and Data Items

Identified articles were imported into EndNote X9 (version X9.3.3, Clarivate Analytics, Philadelphia, PA, USA) to facilitate duplicate removal and organisation. Titles and abstracts were screened for relevance (PC), and a full-text review was conducted on the remaining articles (PC and RP). Discrepancies were resolved through discussion, and excluded studies were documented along with reasons for exclusion (Supplementary Material S3). The search and selection process was illustrated using a PRISMA flow diagram [11].

Key data were extracted as per Campbell et al. [13]. Briefly, this included study authors, design, participant characteristics, method of diagnosis, incidence rate(s), and occupational exposures or risk factors. When reporting incidence rates, fracture incidence is referred to the occurrence of fracture events per unit time (or other exposure denominator) as reported by the original study. Case incidence is reported where the numerator reflects cases as defined by the source study, noting that in some administrative/encounter-based datasets, a single individual may contribute more than one case/event over follow-up. Where studies used inconsistent terminology, we retained the reported estimate but clarified whether it was event/encounter-based or person-based to avoid implying direct comparability. Where possible, risk-related ratios (e.g., odds ratios [ORs], relative risks [RRs], hazard ratios [HRs], and incidence rate ratios [IRRs]) with 95% confidence intervals (CIs) were extracted.

### 2.5. Levels of Evidence and Methodological Quality, Summary of Measures, and Synthesis of Results

Levels of evidence and assessment of methodological quality were classified using the framework described by Campbell et al. [13]. Briefly, the level of evidence provided by each study was ascertained based on the National Health and Medical Research Council (NHMRC) Evidence Hierarchy [14]. The Joanna Briggs Institute (JBI) critical appraisal tools for cohort studies, quasi-experimental studies and randomised controlled trials were used, as appropriate, to assess the trustworthiness, relevance and results of included studies by two reviewers (PC & EC) [15,16]. Responses to the appraisal questions were recorded as ‘yes,’ ‘no,’ or ‘unclear.’ A scoring system was used, assigning one point for ‘yes’ responses and zero for ‘no’ or ‘unclear’ responses. The overall quality was then described using the approach outlined by Orr et al. [17].

Findings from the included studies were synthesised using a critical narrative approach. A meta-analysis was not performed due to the heterogeneity across the included studies.

## 3. Results

**Figure 1:**
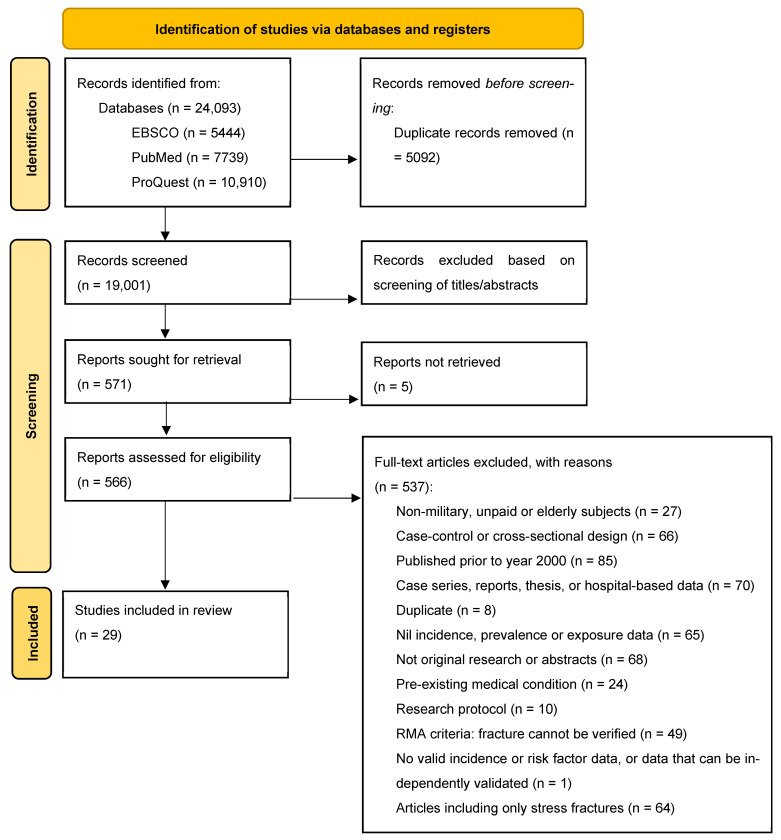
PRISMA diagram showing results of the search, screening, and selection processes [11].

The systematic search initially identified 24,093 studies (Figure 1). After removing duplicates and excluding articles deemed clearly irrelevant during the title and abstract screening, further assessment of full-text articles was conducted to determine eligibility. From this process, a total of 29 eligible articles were identified.

Among the eligible articles, there were 28 cohort studies, consisting of 24 that involved analysis of existing datasets [3,7,18,19,20,21,22,23,24,25,26,27,28,29,30,31,32,33,34,35,36,37,38,39] and 4 where data were collected prospectively [40,41,42,43]. These studies were considered to provide level III-2 and level II evidence, respectively [14]. Further, there was one study that was categorised as quasi-experimental [44]. Complete key data on incidence rates, risk factors, study design, methodological quality, and levels of evidence extracted from all included studies are summarised in Supplementary Materials S4 and S5.

### 3.1. Incidence of Traumatic Fractures in Military Recruits/Trainees

A comparative summary of traumatic fracture incidence in military recruits or trainees by branch of service and nation is provided in Table 3. Claassen et al. [19] reported an overall incidence of 21.8 fractures per 1000 person-years in U.S. recruits across all service branches, with higher rates in females (30.0) than males (20.3). A higher incidence was reported in six companies of U.S. Army recruits during basic training (105.3 per 1000 person-years), predominantly affecting the lower extremities (91.0 vs. 14.3 upper extremity fractures per 1000 person-years) [44]. In United States Military Academy (USMA) cadets from varying institutions, the lower extremity fracture incidence during the first two months of basic training was 36.7 per 1000 person-years [38]. In Israeli Defence Force (IDF) members serving ≥12 months, the incidence was 15.9 cases per 1000 personnel [18]. This figure will have included some new IDF recruits completing initial training, as well as qualified defence personnel, and the rate is not directly comparable to U.S. incidence rate data given the variable exposure times of the IDF members underpinning this IDF fracture rate (exposure times all equal to or greater than 12 months of military service). Finally, in Chinese Navy recruits, the traumatic fracture incidence was 127.9 fractures per 1000 person-years [31].

Traumatic fractures in military recruits predominantly affected the lower limbs, with the foot, ankle and leg bones being the most commonly observed fracture sites across the U.S Military [19,38]. Table 4 provides a synthesis of traumatic fracture incidence rates in military recruits/trainees by anatomical location and population.

### 3.2. Incidence of Traumatic Fractures in Qualified Military Personnel

An overall comparison of the traumatic fracture incidence of non-deployed qualified personnel by branch of service and location of fracture is provided in Table 5, with these data indicating the hand, ankle, forearm, shoulder, and foot [19,20,45] to be the locations most commonly affected by traumatic fractures.

#### 3.2.1. Military Personnel (Non-Deployed or Whole-of-Force)

The overall fracture incidence among qualified U.S. military personnel was higher than that reported in the New Zealand Defence Force (5.50 per 1000 person-years) [3] and the Australian Army (1.9 per 1000 person-years for Regular Army; 1.7 for Reserves) [7], but similar to the IDF (51.0 per 1000 person-years) [20]. Two U.S. studies reported an acute/traumatic fracture incidence of 16.7 and 15.8 per 1000 person-years in active-duty and deployed personnel, respectively [19], while Jones et al. reported a higher incidence of 49.0 per 1000 person-years, likely reflecting inclusion of recruits and potential differences in case definitions [28,45]. In the Greek Armed Forces, static line parachuting fracture rates were higher in recruits (5.3 per 1000 jumps) than officers (1.1 per 1000 jumps), with ankle and shoulder fractures being the most common (47.3% and 25.4%, respectively) [37]. U.S. Army personnel reported a higher overall incidence of fractures (57.6; 95% CI 46.8–69.6) per 1000 soldier-years [42] than the broader U.S. military average (16.7 fractures per 1000 person-years) [19]. In the Chinese Navy, active-duty marines had a higher fracture incidence (86.1 fractures per 1000 person-years) compared to crewmembers (27.0 fractures per 1000 person-years) [31]. While direct comparisons to the IDF are limited by methodological differences, sailors and submariners in the IDF reported incidence rates of 16.1 and 17.4 cases per 1000 person-years, respectively [32]. Anatomical locations most affected by fractures across the U.S. military and IDF include the hand, ankle, forearm, shoulder, and foot [19,20,45].

*Fractures at, or above, the clavicle*

Andreotti et al. [22] reported orbital floor fracture rates of 0.051 and 0.152 per 1000 person-years for hospitalised and ambulatory cases, respectively, which were lower than the overall rates for face fractures reported by Jones et al. [28] at 0.31 and 2.99 per 1000 person-years. Midfacial and orbital blowout fractures in U.S. Army soldiers were reported at rates of 0.34 to 1.00 and 0.07 to 0.61 per 1000 person-years from 1980 to 2000 [36]. Oral-maxillofacial fractures had an incidence rate of 1.2 to 1.4 per 1000 person-years from 2000 to 2005 in active-duty U.S. military personnel [29]. Hsiao et al. [27] reported clavicle fracture rates of 0.91 cases per 1000 person-years in active-duty U.S. military personnel, with higher risk observed in the Marine Corps (IRR 1.44) and Army (IRR 1.16) compared to the Navy (IRR 1.00). Clavicle fracture incidence also decreased with rank, from 0.53 cases per 1000 person-years among E1–E4 ranks to 0.36 cases per 1000 person-years among O1–O3 [27] ranks.

*Fractures of the spine or pelvis*

Lumbar fractures in U.S. personnel were higher compared to cervical fractures with reported rates of 0.38 cases versus 0.29 cases per 1000 person-years [34,35]. The overall incidence of pelvic fractures in U.S. personnel was 0.35 (95% CI, 0.34–0.36) fractures per 1000 person-years [30]. In a population of U.S. Army aviators, the incidence of thoracolumbar fractures was lower than the broader U.S. military rate for pelvic fractures, with a rate of 0.128 fractures per 1000 aviator person-years over a ten-year period [40], suggesting potential differences in fracture risk based on military role.

The pelvic fracture incidence was higher for the Army (IRR 2.45, 95% CI 2.25–2.68) and Marine Corps (IRR 2.22, 95% CI 2.00–2.46) than for the Navy (IRR 1.18, 95% CI 1.06–1.31) and Air Force (IRR 1.00; reference) [30]. Additionally, lumbar and cervical fractures were more common in enlisted personnel than in officers, with incidence rates decreasing as rank increased [34,35]. For example, junior enlisted personnel had incidence rate ratios of 1.63 (95% CI, 1.34–1.98) and 1.93 (95% CI, 1.65–2.26) for cervical and lumbar fractures, respectively, whereas junior officers had IRRs of 1.04 (95% CI, 0.84–1.28) and 1.23 (95% CI, 1.04–1.46), respectively, when each were compared to senior officers (reference group) [34,35].

#### 3.2.2. Military Personnel (Deployed Combat Fractures)

The total fracture incidence in deployed U.S. personnel was 15.8 fractures per 1000 person-years and 16.7 fractures per 1000 person-years in (non-deployed) active-duty personnel [19]. The anatomical locations of the hand (6.1 fractures per 1000 person-years) and foot (3.6 fractures per 1000 person-years) had the highest rates of fracture in deployed U.S. service personnel.

Belmont and colleagues reported a traumatic fracture incidence of 3.41 per 1000 person-years in U.S. active-duty personnel operating in Iraq and Afghanistan [24], compared with 5.0 and 6.4 open and closed fractures per 1000 combat years in U.S. Brigade Combat Team soldiers deployed in OIF [41]. Combat-related axial skeleton and thoracolumbar fracture incidence rates were similar across studies, at 0.57 and 0.21 per 1000 person-years, respectively [24,26], with the case-based combat-related spinal fracture incidence reported as 0.40 per 1000 person-years [33]. Among U.S. combat casualties, Army and Marine Corps personnel had a higher spinal fracture incidence than other branches, with IRRs of 15.45 (95% CI 7.69–31.05) and 12.65 (95% CI 6.18–25.89), respectively, compared with Navy (IRR 6.44, 95% CI 2.80–14.83) and Air Force (IRR 1.00) personnel [33].

### 3.3. Occupational Tasks and Injury Mechanisms Associated with Traumatic Fractures in Military Recruit or Trainee Populations

Eligible studies did not report occupational tasks or injury mechanisms associated with the incidence of traumatic fractures in military recruits/trainees.

### 3.4. Occupational Tasks and Injury Mechanisms Associated with Traumatic Fractures in Qualified Military Personnel

Three studies [23,32,36] evaluated fracture mechanisms in qualified personnel, and three studies [24,26,41] evaluated mechanisms in deployed personnel in active operational areas. In U.S. Army soldiers with midfacial and orbital fractures, violent assault (28.2% and 37.8%), motor vehicle accident (23.7% and 17.6%) and athletics activities (20.1% and 14.1%) were the most common mechanisms [36]. Combat sports were associated with 2.10 traumatic fractures per 1000 person-years, with the highest proportion occurring during martial arts training [23]. Among IDF submariners and sailors, fractures most frequently resulted from striking a person or object (73.7% and 54.2%) and falls (15.7% and 39.4%); sporting activities were the highest-risk context for submariners (47.4%), while working areas and sporting activities were highest risk for sailors on missile boats (30% each) [32].

In deployed U.S. personnel in Iraq and Afghanistan, explosive blasts and gunshots were the most common mechanisms of traumatic fractures, with blasts accounting for 5.5 times more fractures than gunshots [24]. Similar patterns were observed during OIF [41], and improvised explosive devices (IEDs) caused 64.6% of thoracolumbar burst fractures overall and 100% of combat-related thoracolumbar burst fractures [26]. These findings indicate explosive blasts and gunshots are the predominant mechanisms of traumatic fractures in active operational settings.

### 3.5. Other Factors Which Are Associated with Traumatic Fractures in Military Recruit or Trainee Populations

#### 3.5.1. Sex

The female: male incidence rate ratio for traumatic fractures in recruit populations across the entire U.S. military was reported in a single study to be 1.48 [19]. This finding is consistent with the reported female: male odds ratios for lower limb and overall frank fracture occurrence in U.S. Army recruits of 1.68 (95% CI 1.56–1.80) and 1.59 (95% CI 1.51–1.69), respectively [21].

#### 3.5.2. Other Factors

The relationship between traumatic fracture risk and diagnosed ADHD, including those treated using methylphenidate (MP) and untreated personnel, was examined in IDF personnel serving at least 12 months [18]. Higher adjusted odds of traumatic fracture were reported for male and female personnel with untreated ADHD compared with same-sex controls (OR 1.46 and 1.82, respectively) and those with MP-treated ADHD [18]. Among males, MP treatment was also associated with significantly lower odds of fracture compared with controls (adjusted ORs 0.48–0.74, depending on dose), whereas fracture risk did not differ significantly between MP-treated females and female controls [18]. Home of residence ultraviolet exposure was examined as a potential risk factor for traumatic fracture in U.S. Army trainees, based on its role in vitamin D synthesis and bone health; however, no significant association with fracture risk was observed overall or when stratified by sex [21].

### 3.6. Other Factors Which Are Associated with Traumatic Fractures in Qualified Military Personnel

#### 3.6.1. Sex

In U.S. Army personnel, overall traumatic fracture incidence rates were similar in males and females (4.8 [95% CI 3.9–5.9] vs. 5.0 [95% CI 2.1–11] per 1000 soldier-months, respectively) [42]. Separately, male personnel were reported to have significantly higher incidence of oral-maxillofacial fractures than females, with the highest rates observed in the 17–19-year age group (1.93 fractures per 1000 person-years) and decreasing linearly to ≥40 years (0.57 fractures per 1000 person-years) [29]. Claassen et al. reported a higher hand fracture incidence in males (6.32 vs. 3.04 per 1000 person-years) [19], with metacarpal injuries most frequently attributed to punching [28]. Shere et al. similarly identified violent assault as the most common mechanism for midfacial and orbital fractures, which were most frequent in the 20–24-year age group [36].

In the U.S. armed forces, male personnel also had higher incidence rates for lumbar spine fractures than female personnel (IRR 1.19, 95% CI 1.09–1.31) [34], and for cervical spine fractures (IRR 1.45, 95% CI 1.31–1.61) [35]. Conversely, the overall incidence of thoracolumbar fractures was higher in female personnel (0.334 cases per 1000 aviator person-years) than in male personnel (0.122 cases per 1000 aviator person-years), in a study of U.S. Army aviators [40]; however, the result did not reach statistical significance (RR = 2.7; 95% CI 0.7–11.4) [40].

#### 3.6.2. Age

In the U.S. armed forces, pelvic fracture incidence decreased with age, peaking in the youngest group and plateauing after 30–34 years [30]. Additionally, the 20–29-year age group had the highest incidence rates for lumbar fractures [34], cervical spine fractures [35], and clavicle bone fractures [27]. For example, lumbar fracture incidence was higher in the 20–24-year (IRR 1.41, 95% CI 1.21–1.64), 25–29-year (IRR 1.11, 95% CI 1.00–1.24) and >40-year (IRR 1.24, 95% CI 1.10–1.40) age groups when compared to the 35–39-year age bracket (IRR 1.00, reference group) [34]. Cervical fracture incidence was also higher in the 20–24-year (IRR 1.25, 95% CI 1.09–1.43) and 25–29-year (IRR 1.18, 95% CI 1.04–1.33) age groups compared to the 35–39-year age groups (IRR 1.00, reference group) [35]. Clavicle fracture incidence was highest in those aged <30 years, particularly 20–24 years (IRR 1.42, 95% CI 1.28–1.57) compared with >40 years [27].

#### 3.6.3. Race/Ethnicity

Included studies reported significantly higher incidence of pelvic [30], lumbar spine [34], cervical spine [35], and clavicle fractures [27] among U.S. military personnel categorised as ‘white’ compared with those categorised as ‘black’ or ‘other’ race.

## 4. Discussion

The aim of this systematic review was to identify and synthesise current evidence relating to the incidence of and potential risk factors for traumatic fractures in military populations. The available evidence suggested that younger age, male sex, enlisted rank, specific military training activities (e.g., martial arts training), and combat-related factors (e.g., exposure to explosive blasts) may be associated with increased fracture risk. While numerous studies evaluated fracture incidence, fewer employed higher-level study designs which assessed factors and interventions associated with the risk of traumatic fracture occurrence. Consequently, the potential for confounding and incomplete consideration of explanatory variables was substantial, and much of the evidence should be considered hypothesis-generating. Nevertheless, this synthesis provides useful insights into traumatic fracture rates and potential risk patterns that may inform future research and injury prevention efforts aimed at maintaining operational readiness and reducing medical discharge rates.

Evidence on traumatic fracture incidence was predominantly derived from qualified military personnel, with relatively limited data available for recruit or trainee populations. Only five studies reported incidence data in recruits or trainees [18,19,21,31,44], with reported rates in U.S. recruits ranging from 12.2 to 105.3 fractures per 1000 person-years [19,21,44]. The highest reported incidence may reflect differences between service branches or variation in training content and contextual factors during initial entry training. One study reported a higher incidence in the foot/ankle, hand, and leg among whole-of-force recruits [19]; however, this level of anatomical detail was not replicated elsewhere, with other studies reporting overall incidence or broader anatomical groupings. Despite this, available evidence indicated a predominance of lower extremity fractures relative to upper extremity fractures in recruit populations [21,44]. Importantly, the limited evidence base for the recruit subgroup constrains the strength and generalisability of conclusions drawn for this population relative to qualified personnel. Accordingly, findings relating to recruits should be interpreted with caution and may not fully capture variation across training environments, service branches, or nations.

The overall incidences of traumatic fractures in active-duty (but not deployed) qualified military personnel were similar to those for military recruits and trainees. Overall traumatic fracture incidence rates ranged from 16.7 to 57.6 fractures per 1000 person-years in U.S. and IDF personnel [19,20,28,42]. These findings suggest traumatic fracture incidence, in contrast to stress fracture incidence [13], is relatively similar in military recruits and qualified personnel in these nations’ services. However, substantially lower rates of traumatic fractures were observed among qualified personnel of the NZDF (5.50 fractures per 1000 person-years) and Australian Defence Forces (1.9 fractures per 1000 person-years) [3,7]. It is likely the lower reported fracture incidence rates in the NZDF and Australian Defence Forces are largely, if not entirely, due to the data sources used in these studies, when compared to other studies. The NZDF data source was the Accident Compensation Corporation (ACC) database, which records details of injury incidents for which claims are made for compensation or health care costs. It is possible that the ACC database also records only a subset of all injuries that occur to NZDF members who present for health care. The Australian data source was SENTINEL, which is the work health and safety incident reporting system of the Australian Department of Defence and known to record only a small proportion of all injury incidents that result in individuals presenting to ADF health care services [46]. In contrast, most other included studies sourced data from medical records or medical record databases, encompassing records of most or all health care encounters for injuries to individuals comprising the populations or cohorts observed in those studies. It could therefore be expected that incidence rates derived from the latter studies would be higher than incidence rates derived from the ACC and SENTINEL databases. Finally, traumatic fracture incidence among deployed military personnel was generally lower than that reported in non-deployed active-duty populations, suggesting potential differences in personnel characteristics, task profiles and operational contexts [19,24,40,41]. Direct comparisons between branches of service, or between specific sub-occupations within deployed forces, were not reported. Overall, the evidence suggests variation in traumatic fracture incidence between nations and between deployed and non-deployed military settings.

Several other risk factors for fractures at specific anatomical locations among active-duty military personnel were also identified. Active-duty and deployed U.S. Army and Marines personnel were at greater risk of clavicular [27], spinal (overall), pelvic, lumbar and cervical fractures [30,34,35] than active-duty and deployed Navy and Air Force personnel [33]. For clavicular fractures, incidence was highest among enlisted personnel and declined with increasing risk [27]. The increased incidence in lower ranked, younger personnel likely reflects the occupational demands and service roles generally applicable to enlisted ranks and younger personnel. Generally, personnel who were younger had the highest rates of pelvic fractures (<20), lumbar and cervical fractures (20–29 years) and clavicle fractures (20–29 years) [27,30,34,35]. The incidence rates for these fracture types trended down in successive age brackets [19,27,30,34,35].

Across studies, males and females typically had similar rates of traumatic fractures, although there were differences in incidence rates at specific anatomical locations [25,29,34,35]. There were consistent observations of male military recruits and qualified personnel sustaining higher incidence rates of oral, maxillofacial, and hand fractures when compared to their female counterparts [19,25,29,34,35,36]. Additionally, younger age (e.g., <25 years) was a risk factor for traumatic fractures, with risk observed to decrease in older age groups [29]. Enlisted personnel were also at greater risk of traumatic fractures than personnel of higher ranks [25]. The studies providing these findings suggested they were likely a product of violent physical confrontations or assault, which are more prevalent in younger, male, enlisted military personnel [25,29,36]. Male U.S. military servicemembers were also noted to be at higher risk of lumbar (IRR 1.19, 95% CI 1.09–1.31) and cervical spine fractures (IRR 1.45, 95% CI 1.31–1.61) than their female counterparts [35]. These findings are not unexpected given similar observations of greater fracture incidence rates within civilian populations in males when compared to females [1,47,48,49]; however, in civilian populations, this sex-based difference generally reverses once civilians reach around 50–55 years of age, in part due to higher prevalence of osteoporosis in older women than in older men [1]. The sex-related differences in fracture incidence rates observed in younger people, like those in the military, are likely to arise from the greater propensity for physical confrontations, risk taking, manual or more physically strenuous occupations, and/or alcohol use in males [1].

In *non-deployed* occupational settings of the U.S. Army and IDF sailors and submariners, fractures were observed to occur predominantly during athletic activities or in other sporting events and settings [3,32,36], during physical training, or as a result of impact with another individual [32], or motor vehicle accidents [36]. Conversely, within *deployed* U.S. military personnel operating in Iraq and Afghanistan, explosives were most commonly responsible for traumatic fractures, followed by gunshot wounds [24,41]. For thoracolumbar fractures, specifically, IEDs were almost exclusively responsible for these fractures occurring in combat, and for around 65% of all thoracolumbar fractures reported among deployed U.S. military personnel [26]. These context-specific differences suggest that fracture risk may be strongly influenced by operational environments, many aspects of which may be difficult to modify. However, detail regarding specific exposures relating to these environments and the associated activities undertaken by military personnel (i.e., movements, obstacles, etc.) that lead to traumatic fracture is generally lacking within the existing evidence base.

## 5. Limitations

Despite the substantial volume of evidence on fracture incidence rates and risk factors, high-quality data specifically examining occupational task exposures as fracture risk factors were generally lacking within the included studies. More broadly, there was also substantial heterogeneity across included studies in terms of population characteristics, fracture definitions, and exposure denominators, which limited comparability and precluded quantitative synthesis. As such, findings should be interpreted with caution, particularly when comparing incidence estimates across studies or populations.

In addition to these sources of heterogeneity, a substantial challenge was the difficulty in determining whether the reported incidence rates referred specifically to fracture incidence rates or case incidence rates. Many of the included studies provided limited detail on how incidence rates were calculated and used the term ‘case’ inconsistently. For instance, ‘case’ was used to describe an individual who experienced one or more fractures over the study period, an individual who sustained one or more fractures within a specific time frame (e.g., a week) during the study period, or a clinical case representing a single fracture episode or diagnosis—thereby allowing a single individual to contribute multiple clinical ‘cases’ to the overall incident count. These inconsistencies underscore the challenges of synthesising evidence from a broad and heterogeneous body of research, including variations in sample characteristics, study designs, and methodologies. Notably, few of the included studies provided a commentary or analysis on these potential sources of variability. Future primary research should aim to incorporate and, where possible, adjust for additional contextual details and explanatory variables to better account for this variability and identify underlying reasons or hypotheses. Authors should also provide precise definitions of terms like ‘case’ and offer detailed descriptions of the methods used to estimate incidence rates.

Additionally, restricting inclusion to longitudinal designs may have reduced the breadth of evidence available to identify risk factors for traumatic fractures. Accordingly, the risk factors summarised in this review should be interpreted as reflecting those evaluated in longitudinal studies and should not be considered an exhaustive catalogue of all hypothesised traumatic fracture risk factors reported in the wider epidemiological literature.

## 6. Conclusions

This review identified that incidence rates were broadly comparable between recruits and qualified personnel, and higher than among deployed personnel, particularly for lower limb fractures (excluding pelvic fractures). The hands, feet and lower legs exhibited the highest incidence. Identified risk factors included enlisted status, Army or Marine Corps service, combat occupations, younger age (18–29 years), and male sex. Common mechanisms involved sport, physical training, motor vehicle incidents, collisions, slips and occupational tasks, whereas fractures in active combat zones were predominantly due to explosives and gunshot wounds. However, evidence describing injury mechanisms in recruit or trainee populations was limited. This represents an important gap in the literature, as understanding the specific exposures and mechanisms underpinning fractures during initial training is critical for informing targeted prevention strategies. Future large-scale, prospective studies should be conducted to better understand fracture injury mechanisms to develop strategies which modify the exposure risk for military populations. Based on the findings of this review, these studies may be focused on Army and Marine Corps personnel, particularly during training and combat operations.

## Figures and Tables

**Table 1:** An example of the search string used in PubMed—a title/abstract filter was used.

Database	Search Terms	Filters (PubMed)
PubMed	(risk[Title/Abstract] OR predict*[Title/Abstract] OR prevalence[Title/Abstract] OR incidence[Title/Abstract] OR caus*[Title/Abstract] OR etiol*[Title/Abstract] OR frequenc*[Title/Abstract] OR rate*[Title/Abstract] OR mediat*[Title/Abstract] OR exposure*[Title/Abstract] OR likelihood[Title/Abstract] OR probability[Title/Abstract] OR factor[Title/Abstract] OR factors[Title/Abstract] OR hazard[Title/Abstract] OR hazards[Title/Abstract] OR predisposing[Title/Abstract]) AND (work*[Title/Abstract] OR occupation*[Title/Abstract] OR profession*[Title/Abstract] OR trade[Title/Abstract] OR employ*[Title/Abstract] OR military[Title/Abstract] OR Defence[Title/Abstract] OR Defense[Title/Abstract] OR airforce[Title/Abstract] OR “air force”[Title/Abstract] OR army[Title/Abstract] OR navy[Title/Abstract] OR recruit[Title/Abstract] OR soldier*[Title/Abstract] OR marines[Title/Abstract] OR “Military Personnel”[Title/Abstract]) AND (Fracture*[Title/Abstract] OR stress fracture*[Title/Abstract] OR overuse fracture*[Title/Abstract] OR bone stress*[Title/Abstract] OR bone strain*[Title/Abstract])	English, Portuguese, Italian, Spanish Languages, Humans

**Table 2:** Inclusion and exclusion criteria.

Inclusion	Exclusion
Studies reporting original quantitative research involving humans aged 16 years or older, in which cohorts of participants were followed over time in a longitudinal study design (for example, cohort studies, randomised controlled trials, and quasi-experimental studies).	Literature reviews of any type, cross-sectional studies, and case–control studies (except any nested within cohort studies).
Studies published in English, or translatable to English from Portuguese, Spanish, Italian, or French, by members of the research team.	Published abstracts; non-peer-reviewed articles and reports; not original research.
Studies investigating factors, or exposures, or hazards, or causes, or mediators associated with the development or prevention of fractures in personnel engaged in military occupations, or the incidence, or prevalence, or likelihood of the condition occurring in military occupational groups.	Studies of pharmacologic interventions or ergogenic aids.
Studies using diagnostic criteria consistent with the criteria proposed by the Repatriation Medical Authority’s Statements of Principles for fractures, as follows: (a) Means an acquired break or rupture of bone. (b) Excludes spondylolysis, pathological fractures, periostitis, stress fractures due to insufficiency of the bone, and bone stress injuries/bone marrow oedema not being a stress fracture.	Studies of unpaid elite athletes, volunteer occupations, or non-military occupations. Studies involving populations with pre-existing medical conditions or defined based on clinic attendance or hospital admission, studies in elderly populations, unpublished theses, research protocols, or studies published before 2000.

**Table 3:** Military recruit traumatic fracture incidence rate.

Military	Branch	Male Incidence Rate * (Fracture; Case-Based)	Female Incidence Rate * (Fracture; Case-Based)	Combined Incidence Rate *	Incidence Rate Ratio (Female: Male)
United States	Army	105.3 [44]; 11.1 [21]	N/A; 17.5 [21]	N/A; 12.2 [21]	
	United States Marine Academy Cadets			36.7 [38]; N/A	
	All	20.3 [19]; N/A	30.0 [19]; N/A	21.8 [19]; N/A	1.48 [19]
China	Navy	127.9 [31]; N/A			

* Per 1000 person-years.

**Table 4:** Fractures in military recruits by anatomical location.

Anatomical Location	Population	Fractures (Per 1000 Person-Years)
Foot/Ankle	U.S. Military ♂♀	8.1 fractures [19]
Hand	U.S. Military ♂♀	6.1 fractures [19]
Leg	U.S. Military ♂♀	2.6 fractures [19]
Arm	U.S. Military ♂♀	1.6 fractures [19]
Head	U.S. Military ♂♀	1 fracture [19]
Ribs	U.S. Military ♂♀	0.7 fractures [19]
Vertebra	U.S. Military ♂♀	0.5 fractures [19]
Pelvis	U.S. Military ♂♀	0.4 fractures [19]
Shoulder	U.S. Military ♂♀	0.4 fractures [19]
Hip	U.S. Military Cadets ♂♀	1.83 fractures [38]
Thigh	U.S. Military Cadets ♂♀	3.67 fractures [38]
Knee	U.S. Military Cadets ♂♀	0 fractures [38]
Lower Leg/Ankle	U.S. Military Cadets ♂♀	14.70 fractures [38]
Foot/Toes	U.S. Military Cadets ♂♀	15.90 fractures [38]

♂: Male recruits; ♀: female recruits; U.S.: United States.

**Table 5:** Qualified male and female non-deployed military personnel fracture incidence rate.

Military	Branch	Lower Extremity	Upper Extremity	Torso	Facial/Orbital Fracture	All Fracture
United States	Army	0.38 [30]; N/A	N/A; 0.45 [27]	0.128 [40]; 0.32–0.48 [34,35]	0.22–0.73 [36]; N/A	57.6 [42]; N/A
	Marine Corps	0.2 [30]; N/A	N/A; 0.55 [27]	N/A; 0.40–0.46 [34,35]		
	Air Force	0.15 [30]; N/A	N/A; 0.41 [27]	N/A; 0.22–0.28 [34,35]		
	Navy	0.19 [30]; N/A	N/A; 0.39 [27]	N/A; 0.27–0.29 [34,35]		
	All	0.35 [30]; N/A	N/A; 0.91 [27]	N/A; 0.29–0.38 [34,35]	0.152–2.99 [22,28,29]; N/A	16.7–46.6 [19,28]; N/A
Israel	Infantry & Army					51 [20]; N/A
	Navy					N/A; 16.1–17.4 [32] *
Australia	Army					1.9 [7]; N/A
New Zealand	All					5.50 [3] *; N/A
China	Navy					27 [31] *; N/A
	Marines					86.1 [31] *; N/A
Greece	All					1.1 [37]

* Male only; all fractures presented per 1000 Person-Years.

## Data Availability

No new data were created or analyzed in this study.

## Supplementary Materials

The following supporting information can be downloaded at: https://www.mdpi.com/article/10.3390/healthcare14101322/s1, S1: Amendment to protocol; S2: Full search strategy; S3: Full-text excluded articles with reasons; S4: Characteristics and key findings of studies of traumatic fractures in military recruit/trainee populations; S5: Characteristics and key findings of studies of traumatic fractures in qualified military personnel and studies where recruit and qualified military populations could not be separated due to the time-periods of observation; S6: PRISMA 2020 Checklist.
