# Supplementary material for: The Incidence and Risk Factors for the Development of Fractures in Military Populations: A Systematic Review
Source: Healthcare (Basel). 2026 May 13;14(10):1322. doi: 10.3390/healthcare14101322 (PMC13205265; doi:10.3390/healthcare14101322)
Supplement: Supplementary file 1 [file healthcare-14-01322-s001.zip › Suppl. Material S5 - Data Extraction Table (personnel).pdf]

Characteristics and key findings of studies of traumatic fractures in qualified military personnel

Study	Study Design	Participants	Methods (Diagnosis / Exposure to Risk Factors)	Occupations or occupational tasks: comparative levels of incidence or prevalence	Other contextual or risk factors	Study Quality Scores #
Andreotti, Lange & Brundage (2001) [25]<br><br>*Country of origin: United States of America*	Retrospective Cohort	All members of the U.S. Armed Forces between 1/01/1998 – 31/12/1998 (N = 1.6 million, 17 – 65 years)	Data sourced from the Defense Medical Surveillance System (DMED). ICD-9 codes relating to fracture included 802.6-802.7 (orbital floor fractures)	**Incidence of orbital floor fractures (fractures per 1,000 person-years)**<br>**Overall: 0.203**<br><br>**Category** / **Incidence (95% CI)**<br>Hospitalised: 0.051 (0.040-0.064)<br>Resulting in Ambulatory Visits: 0.152 (0.131-0.173)		78%<br><br>Level of Evidence: III-2
Armed Forces Health Surveillance Center (AFHSC) (2014) [26]<br><br>*Country of origin: United States of America*	Retrospective Cohort	Active components of U.S. Army, Air Force, Marine Corps, Coast Guard personnel	Incident cases and rates of injuries associated with contact sports (i.e. boxing, wrestling, martial arts, unspecified combat sport [e.g., wrestling, judo, unarmed combat training]) recorded between 2010-2013. Supplemental ICD 9-CM and STANAG codes were used to identify cases.	**Incidence rates for primary fractures sustained during combat sports**<br><br>**Category** / **Incidence rate (fractures per 1,000 person years)**<br>All Fractures except skull/face fractures: 0.21<br>Skull/face fractures: 0.04<br><br>**Fractures comprised 12% of all injuries reported in this population to have occurred during combat sports during the study time period; 9.9% of all injuries were fractures of body regions other than the skull/face; 2.1% of all injuries were fractures of the skull/face.**	**Combat sport at time of fracture (all types except skull/face fracture)**<br><br>**Sport** / **Proportion of total fractures (all types except skull/face fractures) (%)**<br>Wrestling: 15.5%<br>Boxing: 12.6%<br>Martial arts: 68.5%<br>Unspecified: 3.4%<br><br>**Combat sport at time of skull/face fracture**<br><br>**Sport** / **Proportion of total skull/face fractures (%)**<br>Wrestling: 14.3%	67%<br><br>Level of Evidence: III-2

Study	Study Design	Participants	Methods (Diagnosis / Exposure to Risk Factors)	Occupations or occupational tasks: comparative levels of incidence or prevalence	Other contextual or risk factors	Study Quality Scores [#]
					Boxing 23.1%<br>Martial arts 59.0%<br>Unspecified 3.6%	
Ben-Ami et al. 2018 [23]<br><br>*Country of origin: Israel*	Retrospective cohort	100,000 randomly selected Israeli Defence Force combat soldiers starting and finishing military service between 1/01/2005 and 31/12/2015.	Randomly sampled soldiers' medical records were queried for exposure information relating to ADHD diagnosis and use of methylphenidate.<br><br>Three cohorts were then formed: soldiers with treated (methylphenidate) ADHD (*n* = 689); soldiers with untreated ADHD (*n* = 762); and control group with no ADHD (*n* = 98,549). The treated ADHD group had a proportion of females almost double that of the other two cohorts (13.9% vs 7.5% and 5.9%).<br><br>ICD-9 codes relating to fracture diagnoses were used to calculate incidence of fractures in each group.	**Overall incidence of traumatic fractures in control group was 4.25 fractures per 1,000 person-months, equating to 51.0 fractures per 1,000 person-years**<br><br>**Control Group: Traumatic fracture incidence by anatomical location (fractures per 1,000 person-years)**<br>**Location — Control Group**<br>Skull & face — 4.8<br>Ribs — 0.4<br>Hand phalanges — 19.2<br>Carpal bones — 5.4<br>Forearm — 4.7<br>Metacarpals — 4.3<br>Ankle — 4.2<br>Foot phalanges — 4.6<br>Metatarsals — 2.0<br>Tibia & fibula — 1.7	**Incidence (fractures per 1,000 person-years) of traumatic fractures in treated and untreated ADHD groups**<br>**Treated ADHD — Untreated ADHD**<br>64.8 — 68.4<br><br>**Adjusted OR for traumatic fractures, by factor category**<br>**Factor - Category — OR**<br>Group - Treated ADHD — 1.03 (1.002-1.05)<br>Group - Untreated ADHD — 1.04 (1.02-1.07)<br>Group - Control — 1.00 (reference)<br>Sex - Female — 0.96 (0.95-0.97)<br>Sex - Male — 1.00 (reference)<br>Anaemia – Yes — 1.001 (0.99-1.01)<br>Anaemia – No — 1.00 (reference)<br>Weight (per additional kg) — 1.0005 (1.0004-1.0010)<br>Age (per additional year) — 0.993 (0.991-0.955)<br>Duration of service (per additional month) — 1.0004 (1.0002-1.0007)	67%<br><br>Level of Evidence: III-2
Belmont Jr et al. 2013 [27]<br><br>*Country of origin: United*	Retrospective Cohort	U.S. Active-duty personnel, including 6092 combat casualties (casualty mean ± SD age 25.8 ± 6.5 years), serving in Iraq and Afghanistan 2005-2009.	Musculoskeletal injuries resulting from combat sourced via the Joint Theater Trauma Registry (JTTR) from 1/01/2005 – 31/12/2009, using ICD-9 codes.	**Fracture incidence rates (fractures per 1,000 person-years) among deployed service members**<br>**Body Location — n — Incidence Rate**<br>Axial skeleton — 1142 — 0.57<br>Upper extremity — 2470 — 1.24	**Mechanism of facture**<br>**Mechanism — Fracture body location — *n***<br>**Explosives** — Axial skeleton — 841<br>Upper extremity — 1917	67%<br><br>Level of Evidence: III-2

Study	Study Design	Participants	Methods (Diagnosis / Exposure to Risk Factors)	Occupations or occupational tasks: comparative levels of incidence or prevalence	Other contextual or risk factors	Study Quality Scores #
States of America				Lower extremity 3182 1.60<br>**Total** **6794** **3.41**<br><br>**Classifications of fractures**<br>**Closed** / **Open** / **Total**<br>2864 (42.15%) / 3930 (57.85%) / *N* = 6794	Lower extremity 2662<br>Total 5420<br>**Gunshot**: Axial skeleton 187; Upper extremity 434; Lower extremity 370; Total 991<br>**Motor vehicle crash**: Axial skeleton 83; Upper extremity 74; Lower extremity 59; Total 216<br>**Other**: Axial skeleton 31; Upper extremity 45; Lower extremity 91; Total 167	
Belmont Jr et al. 2001 [43]<br><br>*Country of origin: United States of America*	Prospective Cohort	U.S. Army aviators participating in medical screening (1987 - 1997). *N* = 33,365 (male *n* = 32,497; female *n* = 868).	Medical history, examinations and aero-medical board documents were queried for ICD-9-CM coding 805.x and 806.x (thoracolumbar fractures).	Overall incidence of thoracolumbar fractures = 0.128 fractures per 1,000 aviator person-years.	Incidence of thoracolumbar fractures among female aviators = 0.334 fractures per 1,000 aviator person-years.<br>Incidence of thoracolumbar fractures among male aviators = 0.122 fractures per 1,000 aviator person-years.<br>Risk of thoracolumbar fractures was not significantly different between male and female aviators (RR = 2.7; 95% CI 0.7 – 11.4).	67%<br>Level of Evidence: II
Belmont Jr et al. 2011 [44]	Prospective Cohort	U.S. Brigade Combat Team (BCT) soldiers deployed in OIF over a 15-month period during the "surge"	Details of casualties from the area of operation were sourced via the Joint Theater Trauma Registry (JTTR) using ICD-9 codes.	Open fracture incidence among deployed BCT soldiers: 5.0 fractures per 1,000 combat-years	**Fracture mechanisms (n)**<br>**Open fracture** / **Closed fracture**<br>**Explosion** 15 / 29	89%<br><br>Level of Evidence: II

<table>
<tr><th>Study</th><th>Study Design</th><th>Participants</th><th>Methods (Diagnosis / Exposure to Risk Factors)</th><th>Occupations or occupational tasks: comparative levels of incidence or prevalence</th><th>Other contextual or risk factors</th><th>Study Quality Scores #</th></tr>
<tr><td>Country of origin: United States of America</td><td></td><td>portion of the war (N = 4,122; males = 3,797; females = 325; mean age 27.0 (range, 18-52) years.</td><td></td><td>Closed fracture incidence among BCT soldiers: 6.4 fractures per 1,000 combat-years</td><td><table>
<tr><td>Gun shot wound</td><td>11</td><td>1</td></tr>
<tr><td>Motor vehicle collision</td><td>0</td><td>0</td></tr>
<tr><td>Other</td><td>0</td><td>3</td></tr>
<tr><td>Total</td><td>26</td><td>33</td></tr>
</table></td><td></td></tr>
<tr><td>Claassen, Hu & Rohrbeck (2014) [10]

Country of origin: United States of America</td><td>Retrospective cohort</td><td>Active U.S. military service members (Army, Navy, Air Force, Marine Corps, Coast Guard), recruits, and deployed members to OEF, OIF or OND. Surveillance period for active and recruit cohorts was 1/01/2003 – 31/12/2012; deployed was 1/01/2008 – 31/12/2012. Coast guard periods were 2007 – 2012.</td><td>Data from the Defense Medical Surveillance System, Theater Medical Data Store, and Transportation Command Regulating and Command and Control Evacuation System were queried for incident ICD-9 codes for fracture types: head, vertebra, ribs, sternum, larynx and trachea, pelvis, arm, hand, leg, foot/ankle, unspecified.</td><td>Overall traumatic/frank fracture incidence rate for active-duty service members was 16.7 fractures per 1,000 person-years
Overall traumatic/frank fracture incidence rate for deployed personnel was 15.8 fractures per 1,000 person-years
Traumatic/frank fracture incidence (fractures per 1,000 person-years) in active and deployed service members, by anatomical location<table>
<tr><th>Location</th><th>Active</th><th>Deployed</th></tr>
<tr><td>Head</td><td>1.5</td><td>1.0</td></tr>
<tr><td>Skull</td><td>0.2</td><td>0.2</td></tr>
<tr><td>Nasal</td><td>0.6</td><td>0.4</td></tr>
<tr><td>Mandible</td><td>0.2</td><td>0.2</td></tr>
<tr><td>Multiple bone, other facial</td><td>0.4</td><td>0.3</td></tr>
<tr><td>Vertebra</td><td>0.6</td><td>0.6</td></tr>
<tr><td>Ribs</td><td>0.4</td><td>0.4</td></tr>
<tr><td>Sternum</td><td>0.0</td><td>0.0</td></tr>
<tr><td>Larynx and trachea</td><td>0.0</td><td>0.0</td></tr>
<tr><td>Pelvis</td><td>0.1</td><td>0.2</td></tr>
<tr><td>Shoulder</td><td>0.7</td><td>0.3</td></tr>
<tr><td>Clavicle</td><td>0.7</td><td>0.2</td></tr>
<tr><td>Scapula</td><td>0.1</td><td>0.1</td></tr>
<tr><td>Arm</td><td>1.8</td><td>1.5</td></tr>
<tr><td>Humerus</td><td>0.3</td><td>0.3</td></tr>
<tr><td>Radius and ulna</td><td>1.5</td><td>1.2</td></tr>
</table></td><td>Traumatic/frank fracture incidence (fractures per 1,000 person-years) in active service members, by location and sex<table>
<tr><th>Location</th><th>Male</th><th>Female</th></tr>
<tr><td>Hand</td><td>6.32</td><td>3.04</td></tr>
<tr><td>Foot</td><td>4.29</td><td>4.71</td></tr>
<tr><td>Arm</td><td>1.80</td><td>1.57</td></tr>
<tr><td>Head</td><td>1.67</td><td>0.67</td></tr>
<tr><td>Leg</td><td>1.16</td><td>0.86</td></tr>
<tr><td>Shoulder</td><td>0.82</td><td>0.28</td></tr>
<tr><td>Vertebra</td><td>0.65</td><td>0.52</td></tr>
<tr><td>Ribs</td><td>0.39</td><td>0.28</td></tr>
<tr><td>Pelvis</td><td>0.13</td><td>0.22</td></tr>
<tr><td>Unspecified</td><td>0.11</td><td>0.11</td></tr>
</table></td><td>67%

Level of Evidence: III-2</td></tr>
</table>

Study	Study Design	Participants	Methods (Diagnosis / Exposure to Risk Factors)	Occupations or occupational tasks: comparative levels of incidence or prevalence	Other contextual or risk factors	Study Quality Scores #
				**Hand** 5.8 6.1<br>Carpal 0.9 0.6<br>Metacarpal 2.4 1.4<br>Phalanges 2.5 4.0<br>Multiple fractures 0.0 0.1<br>**Leg** 1.1 1.4<br>Femur 0.2 0.3<br>Patella 0.1 0.1<br>Tibia & fibula 0.8 0.9<br>**Foot** 4.4 3.6<br>Ankle 1.6 1.2<br>Tarsal & metatarsals 1.8 1.4<br>Phalanges 1.0 1.0<br>**Unspecified** 0.1 0.5		
Davidson et al. 2008 [5]<br>*Country of origin: New Zealand*	Retrospective cohort	New Zealand Defence Force personnel in 2002 - 2003 (N = 10,500 [approx.])	Data for all lower limb injury claims were extracted from the Accident Compensation Corporation between 1/07/2002 – 31/05/2003 for members of the New Zealand Defence Force.	**Overall traumatic/frank fracture incidence rate was estimated from the reported data (excluding fractures with mechanisms suggesting they were stress fractures) to be 5.5 fractures per 1,000 person-years**		67%<br>Level of Evidence: III-2
Dichiera et al. 2016 [28]<br><br>*Country of origin: United States of America*	Retrospective cohort	1st Armored Division of the U.S. Military, fiscal years 2009-2013.	ICD-9 codes 814.0-814.09, 815.0-815.09, 816.0-816.9, 817.0 representing metacarpal fractures were identified in the Armed Forces Health Longitudinal Technology Application and Military Health System Mart systems.<br>The study cohort was identified from the William Beaumont Army Medical Center.<br>The Medical Operational Data System was also used to complete data on injured soldiers, including mechanism/activity of injury.<br>Combat military operational specialties (MOS) included infantry, cavalry, armour,	**Underlying population data were not reported and so incidence rates were not calculable**<br><br>**Distribution of metacarpal fractures, by affected metacarpal**<br>**Metacarpal** ***n* (%)** **Significance**<br>2 15 (4.1%)<br>3 15 (4.1%)<br>4 60 (16.3%)<br>5 277 (75.5%) *0.0005		78%<br><br>Level of Evidence: III-2

<table>
<tr><th>Study</th><th>Study Design</th><th>Participants</th><th>Methods (Diagnosis / Exposure to Risk Factors)</th><th>Occupations or occupational tasks: comparative levels of incidence or prevalence</th><th>Other contextual or risk factors</th><th>Study Quality Scores #</th></tr>
<tr><td></td><td></td><td></td><td>artillery, engineers; noncombat arms included all other categories.</td><td></td><td></td><td></td></tr>
<tr><td>Fraser et al., 2023

Country of Origin: United States</td><td>Retrospective Cohort Study</td><td>All male and female U.S military members, enlisted personnel and officers in each service branch (Army, Navy, Marine Corps and Air Force) between 2006-2015.</td><td>The Defense Medical Epidemiology Database was queried for ICD-9 codes relating to ankle-foot fractures (823 – 825.3).

Fractures: tibia-fibula (823), ankle (824), calcaneus closed (825), calcaneus open (825.1), talus closed (825.21), talus open (825.31), other tarsal and metatarsal bones closed (825.2), other tarsal and metatarsal bones open (825.3).</td><td>Across all occupations, enlisted women had an ankle-foot fracture incidence rate of 7.9 per 1,000 person-years.
Enlisted male service members had an ankle-foot fracture incidence rate of 8.4 per 1,000 person-years.
Overall female officers had an incidence rate of 6.9 per 1,000 person-years.
Overall male officers had an incidence rate of 5.5 per 1,000 person-years.
Incidence of Ankle-Foot Fractures Enlisted
Tibia-Fibula Fractures: Males
<table>
<tr><th></th><th>Army</th><th>Navy</th><th>Air Force</th><th>Marines</th></tr>
<tr><td>Special Operations Forces</td><td>6.94</td><td>7.55</td><td>-</td><td>-</td></tr>
<tr><td>Infantry</td><td>7.06</td><td>3.97</td><td>-</td><td>6.02</td></tr>
<tr><td>Mechanised/ Armor</td><td>4.94</td><td>5.06</td><td>-</td><td>3.74</td></tr>
<tr><td>Artillery/Gunnery</td><td>20.51</td><td>20.63</td><td>-</td><td>21.57</td></tr>
<tr><td>Aviation</td><td>-</td><td>20.24</td><td>26.29</td><td>25.38</td></tr>
<tr><td>Engineers</td><td>27.36</td><td>-</td><td>-</td><td>22.43</td></tr>
<tr><td>Maintenance</td><td>4.86</td><td>4.12</td><td>4.57</td><td>5.27</td></tr>
</table></td><td>Relative risk of fractures for female personnel (referenced to males) in occupations
<table>
<tr><th>Specialty</th><th>RR (95% CI)</th><th>P Value</th></tr>
<tr><td colspan="3">Enlisted</td></tr>
<tr><td>Artillery/Gunnery</td><td>1.10 (1.00–1.21)</td><td>.06</td></tr>
<tr><td>Aviation</td><td>0.84 (0.73–0.96)</td><td><.001</td></tr>
<tr><td>Engineers</td><td>0.84 (0.69-1.01)</td><td>.07</td></tr>
<tr><td>Maintenance</td><td>1.01 (0.97-1.05)</td><td>.66</td></tr>
<tr><td>Administration, Intelligence & Communication</td><td>0.90 (0.87-0.93)</td><td><.001</td></tr>
<tr><td>Logistics</td><td>0.96 (0.91-1.02)</td><td>.16</td></tr>
<tr><td>Maritime/Naval Specialties</td><td>1.00 (0.84-1.19)</td><td>.97</td></tr>
<tr><td>Enlisted Total</td><td>0.93 (0.91-0.95)</td><td><.001</td></tr>
<tr><td>Tibia-Fibula</td><td>0.78 (0.76-0.80)</td><td><.001</td></tr>
<tr><td>Rearfoot</td><td>0.82 (0.77-0.88)</td><td><.001</td></tr>
<tr><td>Forefoot</td><td>1.24 (1.20-1.27)</td><td><.001</td></tr>
<tr><td colspan="3">Officers</td></tr>
</table></td><td>82%
Level of Evidence: III-2</td></tr>
</table>

Study	Study Design	Participants	Methods (Diagnosis / Exposure to Risk Factors)	Occupations or occupational tasks: comparative levels of incidence or prevalence					Other contextual or risk factors			Study Quality Scores #
				Administration, Intelligence, Communication	4.96	3.60	4.21	5.11	Ground/Naval Gunfire	0.98 (0.79-1.22)	.85	
				Logistics	5.46	3.93	4.38	5.26	Aviation	1.20 (0.99-1.45)	.06	
				Maritime/Naval Specialties	42.50	13.39	-	-	Engineering & Maintenance	1.13 (0.99-1.30)	.07	
				**Tibia-Fibula Fractures: Females**					Administration	1.43 (1.25-1.64)	<.001	
				Special Operations Forces	No Incidence Data for these Occupational Categories				Operations & Intelligence	1.07 (0.93-1.24)	.35	
				Infantry					Logistics	1.25 (1.09-1.44)	.001	
				Mechanised/ Armor					Services	1.33 (1.24-1.44)	<.001	
				Artillery/Gunnery	27.47	5.70	-	-	Officer Total	1.25 (1.19-1.30)	<.001	
				Aviation	-	13.27	18.75	9.35	Tibia-Fibula	1.09 (1.03-1.16)	.003	
				Engineers	30.84	-	-	19.89	Rearfoot	1.06 (0.90-1.25)	.46	
				Maintenance	4.96	3.25	3.70	4.39	Forefoot	1.56 (1.46-1.67)	<.001	
				Administration, Intelligence, Comm	4.37	2.70	3.00	4.17	**Relative risk of occupational risk of fractures in tactical athletes contrasted to Enlisted Infantry & Ground and Naval Gunfire Officers**			
									Specialty	RR (95% CI)	*P* Value	
									**Enlisted**			

Study	Study Design	Participants	Methods (Diagnosis / Exposure to Risk Factors)	Occupations or occupational tasks: comparative levels of incidence or prevalence					Other contextual or risk factors			Study Quality Scores #
				unication					Special Operation Forces	0.86 (0.80–0.93)	<.001	
				Logistics	4.66	2.69	3.33	3.96	Mechanised /Armor	0.75 (0.70–0.81)	<.001	
				Maritime/Naval Specialties	3.86	1.70	-	-	Artillery/Gunnery	2.88 (2.80–2.96)	<.001	
				**Rearfoot Fractures: Males**					Aviation	3.11 (3.00–3.22)	<.001	
				Special Operations Forces	1.05	0.91	-	-	Engineers	3.67 (3.56–3.79)	<.001	
				Infantry	1.28	0.22	-	1.02	Maintenance	0.69 (0.68–0.71)	<.001	
				Mechanised/ Armor	1.10	0.63	-	1.05	Administration, Intelligence & Communication	0.67 (0.66–0.69)	<.001	
				Artillery/Gunnery	2.78	1.09	-	3.43	Logistics	0.73 (0.71–0.75)	<.001	
				Aviation	-	1.09	3.80	1.90	Maritime/Naval Specialties	0.61 (0.57–0.66)	<.001	
				Engineers	5.80	-	-	3.44	**Officers**			
				Maintenance	0.76	0.57	0.75	0.76	Aviation	0.71 (0.67–0.76)	<.001	
				Administration, Intelligence, Communication	0.75	0.50	0.76	0.61	Engineering & Maintenance	0.89 (0.84–0.95)	<.001	
									Administration	0.87 (0.81–0.94)	<.001	

Study	Study Design	Participants	Methods (Diagnosis / Exposure to Risk Factors)	Occupations or occupational tasks: comparative levels of incidence or prevalence	Other contextual or risk factors	Study Quality Scores #
				(see tables below)	(see tables below)	

Logistics	0.77	0.58	0.70	0.74
Maritime/Naval Specialties	1.39	0.80	-	-
**Rearfoot Fractures: Females**				
Special Operations Forces	No Incidence Data for these Occupational Categories			
Infantry				
Mechanised/ Armor				
Artillery/Gunnery	3.30	1.81	-	-
Aviation	-	2.65	1.63	0.00
Engineers	2.80	-	-	0.64
Maintenance	0.95	0.52	0.62	0.37
Administration, Intelligence, Communication	0.54	0.40	0.46	0.56

Operations & Intelligence	0.83 (0.78–0.89)		<.001			
Logistics	0.92 (0.86–0.99)		.02			
Services	0.90 (0.86–0.96)		<.001			
**Analysis of Sex (Female-Male), Service Branch (US Army), Rank (Enlisted) and Year (2006) on Fracture Risk**						
	Tibia-Fibula		Rearfoot		Forefoot	
	95% CI		95% CI		95% CI	
	RR	*P*	RR	*P*	RR	*P*
Female Sex	0.96	.13	0.96	.69	1.54	<.001
**Branch**						
Navy	0.75	<.001	0.85	0.87	0.87	.002
Marine Corps	1.03	.40	0.97	1.42	1.42	<.001
Air Force	0.83	<.001	0.86	0.94	0.94	.15
Officer Rank	0.76	<.001	0.64	0.77	0.77	<.001

Study	Study Design	Participants	Methods (Diagnosis / Exposure to Risk Factors)	Occupations or occupational tasks: comparative levels of incidence or prevalence					Other contextual or risk factors							Study Quality Scores [#]
									Year							
				Logistics	0.77	0.30	0.62	0.64	2007	0.89	.07	0.91	.67	0.93	.30	
				Maritime/Naval Specialties	3.86	0.88	-	-	2008	0.90	.08	0.86	.53	0.96	.54	
				**Forefoot Fractures: Males**					2009	0.88	.04	1.10	.68	0.94	.37	
				Special Operations Forces	1.43	1.51	-	-	2010	0.97	.63	1.07	.77	0.84	.02	
				Infantry	3.41	1.65	-	2.87	2011	0.82	.001	1.09	.71	0.91	.22	
				Mechanised/Armor	2.46	1.90	-	3.10	2012	0.89	.06	0.96	.87	0.89	.12	
				Artillery/Gunnery	8.21	6.97	-	10.60	2013	0.81	.001	0.95	.83	0.88	.08	
				Aviation	-	7.13	10.80	8.63	2014	0.91	.14	1.23	.37	0.83	.01	
				Engineers	9.89	-	-	8.46	2015	.86	.02	0.89	.63	0.83	.01	
				Maintenance	2.62	2.07	2.41	2.90								
				Administration, Intelligence, Communication	2.47	2.02	2.26	2.78								
				Logistics	2.61	1.92	2.40	2.80								
				Maritime/Naval	32.84	11.85	-	-								

Study	Study Design	Participants	Methods (Diagnosis / Exposure to Risk Factors)	Occupations or occupational tasks: comparative levels of incidence or prevalence					Other contextual or risk factors	Study Quality Scores #
				Specialties						
				**Forefoot Fractures: Females**						
				Special Operations Forces	No Incidence Data for these Occupational Categories					
				Infantry						
				Mechanised/ Armor						
				Artillery/Gunnery	12.45	9.58	-	-		
				Aviation	-	8.40	17.12	22.45		
				Engineers	1.40	-	-	12.83		
				Maintenance	3.69	3.03	2.98	4.12		
				Administration, Intelligence, Communication	2.82	2.36	2.95	3.47		
				Logistics	3.57	2.34	2.86	5.08		
				Maritime/Naval Specialties	1.93	2.38	-	-		
				**Total Ankle-Foot Fractures: Males**						
				Special Operations Forces	9.43	10.04	-	-		
				Infantry	11.75	5.84	-	9.91		

Study	Study Design	Participants	Methods (Diagnosis / Exposure to Risk Factors)	Occupations or occupational tasks: comparative levels of incidence or prevalence					Other contextual or risk factors	Study Quality Scores #
				Mechanised/ Armor	8.50	7.59	-	7.88		
				Artillery/Gunnery	31.51	28.68	-	35.59		
				Aviation	-	28.46	40.89	35.91		
				Engineers	43.05	-	-	34.32		
				Maintenance	8.24	6.76	7.73	8.92		
				Administration, Intelligence, Communication	8.19	6.12	7.22	8.51		
				Logistics	8.84	6.43	7.49	8.80		
				Maritime/Naval Specialties	10.41	6.62	-	-		
				**Total Ankle-Foot Fractures: Females**						
				Special Operations Forces	-	-	-	-		
				Infantry	-	-	-	-		
				Mechanised/ Armor	-	-	-	-		
				Artillery/Gunnery	43.22	17.09	-	-		
				Aviation	-	24.33	37.51	31.80		
				Engineers	35.04	-	-	33.37		

Study	Study Design	Participants	Methods (Diagnosis / Exposure to Risk Factors)	Occupations or occupational tasks: comparative levels of incidence or prevalence					Other contextual or risk factors	Study Quality Scores #
				Maintenance	9.59	6.80	7.31	8.88		
				Administration, Intelligence, Communication	7.73	5.47	6.41	8.20		
				Logistics	9.00	5.32	6.82	9.68		
				Maritime/Naval Specialties	13.52	6.65	-	-		
				**Officers**						
				**Tibia-Fibula Fractures: Males**						
				Ground and Naval Gunfire	4.66	2.79	3.83	3.43		
				Aviation	3.63	2.46	2.83	2.67		
				Engineering & Maintenance	3.51	3.09	3.37	3.60		
				Administration	3.09	2.56	3.09	3.51		
				Operations & Intelligence	3.26	2.67	3.00	3.25		
				Logistics	3.08	2.54	3.68	3.46		
				Services	3.17	2.53	2.96	1.60		
				**Tibia-Fibula Fractures: Females**						
				Ground and	3.39	2.50	1.43	11.72		

Study	Study Design	Participants	Methods (Diagnosis / Exposure to Risk Factors)	Occupations or occupational tasks: comparative levels of incidence or prevalence					Other contextual or risk factors	Study Quality Scores #
				Naval Gunfire						
				Aviation	2.73	2.53	3.64	2.42		
				Engineering & Maintenance	3.54	2.18	3.97	4.50		
				Administration	4.58	5.25	3.25	3.75		
				Operations & Intelligence	2.96	1.31	3.03	3.18		
				Logistics	4.14	3.43	4.03	2.98		
				Services	3.77	3.18	3.69	1.44		
				**Rearfoot Fractures: Males**						
				Ground and Naval Gunfire	0.72	0.35	0.18	0.41		
				Aviation	0.53	0.38	0.33	0.28		
				Engineering & Maintenance	0.42	0.48	0.41	0.32		
				Administration	0.26	0.30	0.21	0.80		
				Operations & Intelligence	0.38	0.21	0.61	0.37		
				Logistics	0.39	0.27	0.85	0.45		
				Services	0.47	0.45	0.49	0.60		
				**Rearfoot Fractures: Females**						

Study	Study Design	Participants	Methods (Diagnosis / Exposure to Risk Factors)	Occupations or occupational tasks: comparative levels of incidence or prevalence					Other contextual or risk factors	Study Quality Scores #
				Ground and Naval Gunfire	1.69	0.20	1.43	4.69		
				Aviation	0.21	0.34	0.36	0.00		
				Engineering & Maintenance	0.44	0.94	0.17	0.00		
				Administration	0.60	1.27	0.30	0.00		
				Operations & Intelligence	0.15	0.66	0.41	0.80		
				Logistics	0.60	0.86	0.39	0.00		
				Services	0.49	0.52	0.46	0.00		
				**Forefoot Fractures: Males**						
				Ground and Naval Gunfire	2.29	1.64	1.28	1.55		
				Aviation	1.42	1.55	1.26	1.04		
				Engineering & Maintenance	2.21	1.36	1.95	2.09		
				Administration	1.94	1.28	1.83	1.70		
				Operations & Intelligence	2.17	1.72	1.80	1.55		
				Logistics	1.95	1.81	2.32	2.28		

Study	Study Design	Participants	Methods (Diagnosis / Exposure to Risk Factors)	Occupations or occupational tasks: comparative levels of incidence or prevalence					Other contextual or risk factors	Study Quality Scores #
				Services	1.90	1.80	2.12	2.80		
				**Forefoot Fractures: Females**						
				Ground and Naval Gunfire	1.13	3.20	5.72	0.00		
				Aviation	1.89	2.03	2.31	4.03		
				Engineering & Maintenance	2.16	2.18	3.54	0.90		
				Administration	2.82	2.54	2.65	2.05		
				Operations & Intelligence	2.81	3.28	2.33	3.18		
				Logistics	2.39	2.00	3.84	2.65		
				Services	2.96	3.07	3.21	5.76		
				**Total Ankle-Foot Fractures: Males**						
				Ground and Naval Gunfire	7.68	4.78	5.28	5.38		
				Aviation	5.58	4.39	4.42	3.99		
				Engineering & Maintenance	6.14	4.93	5.73	6.01		
				Administration	5.29	4.14	5.14	6.01		
				Operations & Intelligence	5.80	4.60	5.41	5.18		

Study	Study Design	Participants	Methods (Diagnosis / Exposure to Risk Factors)	Occupations or occupational tasks: comparative levels of incidence or prevalence					Other contextual or risk factors			Study Quality Scores #
				Logistics	5.41	4.62	6.84	6.19				
				Services	5.55	4.78	5.57	4.99				
				**Total Ankle-Foot Fractures: Females**								
				Ground and Naval Gunfire	6.21	5.90	8.57	16.41				
				Aviation	4.83	4.90	6.31	6.45				
				Engineering & Maintenance	6.14	5.30	7.68	5.41				
				Administration	8.01	9.06	6.20	5.80				
				Operations & Intelligence	5.92	5.25	5.77	7.17				
				Logistics	7.13	6.29	8.26	5.64				
				Services	7.22	6.77	7.36	7.20				
Freedman et al. 2014 [29]<br><br>*Country of origin: United States of America*	Retrospective cohort	U.S. Military personnel deployed in Iraq and Afghanistan during the observational period (Aug 15 2009 – 2010). Historical cohorts for comparison were identified for the periods Aug 15 2007-2008 and Aug 15 2008-2009.	Department of Defense Trauma Registry was queried for ICD-9 codes indicating a thoracolumbar fracture (805.2-5, 806.2-5, 953, 344.1, 336.8, 344.6, 344.61).	Incidence of thoracolumbar fracture was 0.21 fractures per 1,000 soldier-years<br><br>Thoracolumbar fractures comprised 3.0% of combat-casualty admissions to Landstuhl Regional Medical Center					**Injury Mechanism for thoracolumbar burst fracture (n[%])**			55%<br><br>Level of Evidence: III-2
									**Mechanism**	**Among all mechanisms**	**Among combat mechanisms**	
									IED	42 (64.6%)	42 (100%)	
									All other	23 (35.4%)	0 (0%)	
Hsiao et al. 2012 [30]<br><br>*Country of origin:*	Retrospective cohort	Active-duty U.S. Military personnel from 1999 – 2008 (*n* = 12,514 clavicle fractures)	Defense Medical Epidemiological Database was queried for ICD-9 code 810.0 (clavicle fractures) between 1999 – 2008	Overall *case* incidence rate for *clavicle* fractures was 0.91 (95% CI, 0.90 – 0.91) cases per 1,000 person-years					**Clavicle fracture adjusted *case* incidence rates (cases per 1,000 person-years) by Rank Level**			89%<br><br>Level of Evidence: III-2
									**Rank**	**aIR**	**aIRR (95% CI)**	

<table>
<tr><th>Study</th><th>Study Design</th><th>Participants</th><th>Methods (Diagnosis / Exposure to Risk Factors)</th><th>Occupations or occupational tasks: comparative levels of incidence or prevalence</th><th>Other contextual or risk factors</th><th>Study Quality Scores #</th></tr>
<tr>
<td>United States of America</td>
<td></td>
<td></td>
<td></td>
<td>
Clavicle fracture adjusted case incidence rates (cases per 1,000 person-years) by Branch of Service
<table>
<tr><th>Service</th><th>aIR</th><th>aIRR (95% CI)</th></tr>
<tr><td>Marines</td><td>0.55</td><td>1.44 (1.35-1.53)</td></tr>
<tr><td>Army</td><td>0.45</td><td>1.16 (1.10-1.23)</td></tr>
<tr><td>Airforce</td><td>0.41</td><td>1.06 (1.00-1.13)</td></tr>
<tr><td>Navy</td><td>0.39</td><td>1.00 (Reference)</td></tr>
</table>
IR and IRR adjusted for sex, race, age, and rank
</td>
<td>
<table>
<tr><td>E1-E4</td><td>0.53</td><td>1.46 (1.34-1.60)</td></tr>
<tr><td>E5-E9</td><td>0.49</td><td>1.35 (1.24-1.47)</td></tr>
<tr><td>O4-O9</td><td>0.41</td><td>1.12 (0.99-1.28)</td></tr>
<tr><td>O1-O3</td><td>0.36</td><td>1.00 (Reference)</td></tr>
</table>
IR and IRR adjusted for sex, race, age and rank

Clavicle fracture adjusted case incidence rates (cases per 1,000 person-years) by Age category
<table>
<tr><th>Age</th><th>aIR</th><th>aIRR (95% CI)</th></tr>
<tr><td><20</td><td>0.52</td><td>1.38 (1.22-1.55)</td></tr>
<tr><td>20-24</td><td>0.54</td><td>1.42 (1.28-1.57)</td></tr>
<tr><td>25-29</td><td>0.45</td><td>1.18 (1.07-1.30)</td></tr>
<tr><td>30-34</td><td>0.41</td><td>1.08 (0.98-1.19)</td></tr>
<tr><td>35-39</td><td>0.39</td><td>1.01 (0.92-1.12)</td></tr>
<tr><td>>40</td><td>0.38</td><td>1.00 (Reference)</td></tr>
</table>
IR and IRR adjusted for sex, race, age and rank

Clavicle fracture adjusted case incidence rates (cases per 1,000 person-years) by race category
<table>
<tr><th>Race category</th><th>Race category</th><th>Race category</th></tr>
<tr><td>White</td><td>0.66</td><td>2.45 (2.29-2.63)</td></tr>
</table>
</td>
<td></td>
</tr>
</table>

Study	Study Design	Participants	Methods (Diagnosis / Exposure to Risk Factors)	Occupations or occupational tasks: comparative levels of incidence or prevalence	Other contextual or risk factors	Study Quality Scores #
					Other 0.49 1.81 (1.66-1.99)<br>Black 0.27 1.00 (Reference)<br>*IR and IRR adjusted for sex, race, age and rank*	
Jones et al. 2010 [31]<br><br>*Country of origin: United States of America*	Retrospective cohort	Non-deployed members of the U.S. Army (*n* = 358,524), Air Force (*n* = 318,312), Navy (*n* = 318,805), and Marines (*n* = 149,647), 1/01/2006 – 31/12/2006 (total N = 1,145,289)	The Defense Medical Surveillance System was queried for ICD-9 codes relating to fracture in the calendar year 2006 and hospitalisation data was also extracted from the database. Steps were taken to exclude follow-up visits in injury counts (60-day exclusion rule), so that only new instances of injury were counted.	Total combined hospitalised and ambulatory acute/traumatic fracture incidence was 49.0 fractures per 1,000 person-years<br>Hospitalised acute/traumatic fracture incidence was 2.40 fractures per 1,000 person-years<br>Ambulatory acute/traumatic fracture incidence was 46.6 fractures per 1,000 person-years<br><br>**Hospitalised acute/traumatic fracture incidence by body location (fractures per 1,000 person-years)**<br>**Location / Type** — **Fractures (n)** — **Incidence**<br>**Traumatic brain injury associated**<br>Type 1 — 117 — 0.10<br>Type 2 — 49 — 0.04<br>Type 3 — 11 — 0.01<br>**Other head**<br>Face — 357 — 0.31<br>Neck — 2 — 0.002<br><br>**Spine and back – spinal cord**<br>Cervical — 18 — 0.02<br>Thoracic — 21 — 0.02<br>Lumbar — 7 — 0.01<br>**Vertebral column**<br>Cervical — 50 — 0.04<br>Thoracic — 52 — 0.05<br>Lumbar — 89 — 0.08		56%<br><br>Level of Evidence: III-2

Study	Study Design	Participants	Methods (Diagnosis / Exposure to Risk Factors)	Occupations or occupational tasks: comparative levels of incidence or prevalence			Other contextual or risk factors	Study Quality Scores #
				Sacrum coccyx	18	0.02		
				Spine, back, unspecified	4	0.003		
				**Torso**				
				Chest/thorax	66	0.06		
				Pelvis	89	0.08		
				Trunk	3	0.003		
				**Upper extremities**				
				Shoulder, upper arm	182	0.16		
				Forearm, elbow	216	0.19		
				Wrist, hand, fingers	249	0.22		
				Other / unspecified	7	0.01		
				**Lower extremities**				
				Hip	57	0.05		
				Upper leg, thigh	120	0.10		
				Knee	34	0.03		
				Lower leg, ankle	768	0.67		
				Foot, toes	141	0.12		
				Other / unspecified	12	0.01		
				**Unclassified by site**				
				Other / multiple	7	0.01		
				Unspecified site	8	0.01		
				**Ambulatory acute/traumatic fracture incidence by body location (fractures per 1,000 person-years)**				

Study	Study Design	Participants	Methods (Diagnosis / Exposure to Risk Factors)	Occupations or occupational tasks: comparative levels of incidence or prevalence	Other contextual or risk factors	Study Quality Scores [#]
				(see table below)		

Location / Type	Fractures (n)	Incidence rate
**Traumatic brain injury associated**		
Type 1	225	0.20
Type 2	366	0.32
Type 3	77	0.07
**Other head**		
Face	3420	2.99
Neck	5	0.004
**Spine and Back – spinal cord**		
Cervical	75	0.07
Thoracic	291	0.25
Lumbar	53	0.05
Sacrum coccyx	7	0.01
Spine, back, unspecified	10	0.01
**Vertebral column**		
Cervical	361	0.32
Thoracic	335	0.29
Lumbar	609	0.53
Sacrum coccyx	223	0.19
Spine, back, unspecified	110	0.10
**Torso**		
Chest/thorax	1267	1.11
Pelvis	658	0.57
Trunk	11	0.01
**Upper extremities**		
Shoulder, upper arm	2830	2.47
Forearm, elbow	3926	3.43

Study	Study Design	Participants	Methods (Diagnosis / Exposure to Risk Factors)	Occupations or occupational tasks: comparative levels of incidence or prevalence	Other contextual or risk factors	Study Quality Scores #
				Wrist, hand, fingers 16934 14.79<br>Other / unspecified 134 0.12<br>**Lower extremities**<br>Hip 574 0.50<br>Upper leg, thigh 864 0.75<br>Knee 458 0.40<br>Lower leg, ankle 8914 7.78<br>Foot, toes 9161 8.00<br>Other / unspecified 473 0.41<br>**Unclassified by site**<br>Other / multiple 48 0.04<br>Unspecified site 956 0.83		
Mitchener & Canham-Chervak (2010) [32]<br><br>*Country of origin: United States of America*	Retrospective cohort	Active-duty U.S. military personnel, 1996-2005. Fracture incidence relates to the period 2000-2005.	Defense Medical Surveillance System (DMSS) was queried to identify injuries to the oral-facial region of personnel who underwent inpatient or outpatient treatments worldwide.<br><br>ICD-9 codes relating to fracture were used (802.20-802.29, 802.30-802.39, 802.4-0.5, 802.6-0.7, 802.8-0.9).	Overall annual incidence rates for oral-maxillofacial fractures ranged from 1.2 – 1.4 fractures per 1,000 person-years, between 2000 and 2005.	**Incidence rate for oral-maxillofacial fractures (fractures per 1,000 person-years), by sex**<br>**Sex** **IR (mean)** **IR (range)**<br>Male 1.38 1.2-1.5<br>Female 0.85 0.7-1.0*<br>*Males had a significantly higher incidence each year than females (p <0.001)*<br><br>**Incidence rate for oral-maxillofacial fractures (fractures per 1,000 person-years), by age**<br>**Age** **IR (mean)** **IR (range)**<br>17-19 1.93 1.78-2.09<br>20-24 1.85 1.58-1.97<br>25-29 1.18 1.09-1.26<br>30-34 0.78 0.71-0.88<br>35-39 0.65 0.56-0.77<br>≥ 40 0.57 0.47-0.66	67%<br><br>Level of Evidence: III-2
Pisquiy et al. 2020 [33]	Retrospective cohort	U.S. Armed Forces personnel, 2006 – 2015, with a total of	Defense Medical and Epidemiology Database was queried for ICD-9 codes relating	Overall incidence of pelvic fractures was 0.35 (95% CI, 0.34-0.36) pelvic fractures per 1,000 person-years	**Incidence rate for pelvic fractures (pelvic fractures per 1,000 person-years), by age category**	67%

Study	Study Design	Participants	Methods (Diagnosis / Exposure to Risk Factors)	Occupations or occupational tasks: comparative levels of incidence or prevalence	Other contextual or risk factors	Study Quality Scores #
Country of origin: United States of America		13,748,429 person-years of exposure to military service	to pelvic fractures (808). Only ambulatory patient and in-patient data were collected.	**Incidence rates for pelvic fractures (pelvic fractures per 1,000 person-years), by branch of service**<br>**Service Branch** | **Unadjusted IR** | **aIRR* (95% CI)**<br>Army | 0.38 | 2.45 (2.25-2.68)<br>Marines | 0.2 | 2.22 (2.00-2.46)<br>Navy | 0.19 | 1.18 (1.06-1.31)<br>Air Force | 0.15 | 1.00 (reference)<br>*IRR adjusted for sex, race, service and rank*	**Age** | **Unadjusted IR** | **aIRR* (95% CI)**<br>< 20 | 0.59 | 1.00 (reference)<br>20-24 | 0.43 | 0.82 (0.75-0.91)<br>25-29 | 0.32 | 0.74 (0.65-0.83)<br>30-34 | 0.25 | 0.63 (0.55-0.72)<br>35-39 | 0.26 | 0.72 (0.62-0.84)<br>≥ 40 | 0.26 | 0.77 (0.65-0.90)<br>*IRR adjusted for sex, race, service and rank*<br><br>**Incidence rate for pelvic fractures** (pelvic fractures **per 1,000 person-years), by race category**<br>**Race** | **Unadjusted IR** | **aIRR* (95% CI)**<br>White | 0.37 | 1.00 (reference)<br>Other | 0.29 | 0.79 (0.72-0.86)<br>Black | 0.31 | 0.71 (0.65-0.77)<br>*IRR adjusted for age, sex, service, and rank*	Level of Evidence: III-2
Potter et al. 2002 [45]<br><br>*Country of origin:*	Prospective cohort	U.S. Army soldiers of the 82nd Airborne Division, 1/04/1996 – 31/03/1997. Includes two infantry battalions	Medical records of activity duty soldiers were reviewed to identify fracture cases.	Overall traumatic fracture incidence was 4.8 (95% CI 3.9-5.8) fractures per 1,000 soldier-months, equating to 57.6 (95% CI 46.8-69.6) fractures per 1,000 soldier-years	Overall traumatic fracture incidence for male soldiers was 4.8 (95% CI 3.9-5.9) fractures per 1,000 soldier-months	89%<br><br>Level of Evidence: II

Study	Study Design	Participants	Methods (Diagnosis / Exposure to Risk Factors)	Occupations or occupational tasks: comparative levels of incidence or prevalence	Other contextual or risk factors	Study Quality Scores #
United States of America		(*n* = 669), and three combat support battalions (*n* = 1,180 men; *n* = 116 women). Total N = 1965.			Overall traumatic fracture incidence for female soldiers was 5.0 (95% CI 2.1-11) fractures per 1,000 soldier-months<br><br>These fracture incidence rates, by sex, did not differ significantly	
Qi et al. 2016 [34]<br><br>*Country of origin: China*	Retrospective cohort	Active-duty Chinese Navy crewmembers (n=1442), marines (n=956) and recruits (4371) who received training on surface ships deployed to Aden Gulf in the 12-week period	Medical records were reviewed to identify relevant ICD-10 codes. Diagnoses during military-training time-period were made using the Military Training Injury Classification and Diagnosis Criteria of the Chinese People's Liberation Army.	**Note:** There were errors in the IRs calculated for the three cohorts by the study authors. Specifically, the study authors calculated IRs based on 12 months of exposure to training rather than the actual 12 weeks of exposure to training they reported as the time period in which they observed reported fractures. The IRs reported below are therefore recalculated for this review using the primary data reported in the study and reflect the actual 12 weeks of training exposure.<br><br>**Traumatic fracture incidence (fractures per 1,000 person-years) during 12-week military training program**<br>**Crewmember** **Marines** **Recruits**<br>27.0 86.1 127.9<br><br>**Fracture distribution (*n*) during military training**<br>***Lower extremity***<br>**Crewmember** **Marines** **Recruits**<br>5 10 70<br>***Upper extremity***<br>**Crewmember** **Marines** **Recruits**<br>3 7 50<br>***Spine & back***<br>**Crewmember** **Marines** **Recruits**<br>1 2 9		78%<br><br>Level of Evidence: III-2

Study	Study Design	Participants	Methods (Diagnosis / Exposure to Risk Factors)	Occupations or occupational tasks: comparative levels of incidence or prevalence	Other contextual or risk factors	Study Quality Scores [#]
Reynolds et al., 2002 [46]<br><br>*Country of origin: United States of America*	Prospective cohort	Two battalions of the 10th Mountain Division including construction engineers (*n* = 125) and combat artillery soldiers (*n* = 188)	Medical record checks were conducted to identify cases and these consisted of physician- and assistant- documented records collected over a 12-month period.	**The odds ratio (OR) comparing odds of an engineer sustaining a traumatic fracture to odds of a combat artillery soldier sustaining a traumatic fracture was 6.5 (95% CI, 1.8-23.7)**		73%<br><br>Level of Evidence: II
Saad et al. 2015 [35]<br><br>*Country of origin: Israel*	Retrospective cohort	Israeli Defense Force submariners (*n* = 457) and sailors (*n* = 3219); all male, aged 18-42 years.	Computerised medical records from 2002 – 2012. Potential or recurrent fractures were excluded, as were suspected fractures without X-ray confirmation.	The *case* incidence rate for fractures in submariners was 19 cases in 1091.42 person-years of service, equating to 17.4 cases per 1,000 person-years of service.<br><br>The *case* incidence rate for fractures in sailors was 94 cases in 5845.04 person-years of service, equating to 16.1 cases per 1,000 person-years of service<br><br>The Hazard Ratio comparing rates of fracture occurrence in each group was 1.037 and not significant (p= 0.89)	**Cause of fracture (% of all fractures in group)**<br>**Mechanism** / **Submariners** / **Sailors (Missile boats)**<br>Fall / 15.79% / 39.36%<br>Striking person/ object / 73.68% / 54.26%<br>Other / 5.26% / 2.13%<br>Unknown / 5.26% / 4.26%<br>**Location**<br>Working area / 10.53% / 29.98%<br>Sport / 47.37% / 29.98%<br>Other / 31.58% / 18.08%<br>Unknown / 10.53% / 22.34%	73%<br><br>Level of Evidence: III-2
Schermann et al. 2018 [22]<br><br>*Country of origin: Israel*	Retrospective cohort	Israeli Defence Force members (N = 682,110; men = 409,175, women = 272,935) serving at least 12 months (average service period 56.6 months) between 2008-2017, or part thereof (aged 18-25).	Data regarding Methylphenidate (MP) use for ADHD treatment were extracted from prescription data.<br><br>ICD-9 codes were used to extract fracture diagnoses.	7.477% of the 682,110 personnel who served ≥ 12 months (average service period 56.6 months) experienced at least one fracture during the period of follow-up.<br><br>This equates to an estimated overall *case* incidence rate for fractures in these personnel of 15.9 cases per 1,000 person-years of exposure to military service from time of enlistment.	**Adjusted* odds ratios (OR) comparing risks of fracture occurrence in personnel without ADHD, with untreated ADHD, with ADHD treated with MP, and with and without other factors, stratified by sex**<br>**Factor/ Category** / **Men OR** / **Women OR**<br>Without ADHD / 1.00 (reference) / 1.00 (reference)<br>Untreated ADHD / 1.46 / 1.82<br>ADHD, 1-90 MP doses / 0.74 / 1.23 (NS)<br>ADHD, 90-180 MP doses / 0.53 / 0.88 (NS)	78%<br><br>Level of Evidence: III-2

Study	Study Design	Participants	Methods (Diagnosis / Exposure to Risk Factors)	Occupations or occupational tasks: comparative levels of incidence or prevalence	Other contextual or risk factors			Study Quality Scores #
					ADHD, 180+ MP doses	0.48	0.69 (NS)	
					Age (per year increase)	0.84	0.94	
					Weight (per kg increase)	1.004	1.01	
					Israeli origin	1.00 (reference)	1.00 (reference)	
					African, Asian, South American origin	0.99 (NS)	1.18	
					North American, European, former Soviet Union origin	0.87	0.99 (NS)	
					Education (12-years)	1.00 (reference)	1.00 (reference)	
					>12 years education	0.89	0.97 (NS)	
					Low socioeconomic level	1.00 (reference)	1.00 (reference)	
					Middle socioeconomic level	0.90	1.15 (NS)	
					High socioeconomic level	0.88	1.15 (NS)	
					Non-combat service	1.00 (reference)	1.00 (reference)	
					Combat service	1.16	2.41	
					Duration of follow-up (per year increase)	1.0004 (NS)	0.99	

Study	Study Design	Participants	Methods (Diagnosis / Exposure to Risk Factors)	Occupations or occupational tasks: comparative levels of incidence or prevalence	Other contextual or risk factors	Study Quality Scores #
					** adjusted for other included factors, type of military service, and ADHD and MP exposures*	
Schoenfeld et al. 2013a [36]<br><br>*Country of origin: United States of America*	Retrospective cohort	U.S. Military, 2005-2009. In this population, 7,877 combat casualties sustaining spinal injuries were recorded in the period 2005 – 2009.	Personnel with spinal injuries sustained in Iraq and Afghanistan were manually identified through a search of the Department of Defense Trauma Registry	**Overall *case* incidence rate for *spinal* fractures was 0.40 cases per 1,000 person-years**<br><br>***Case* incidence rate for spinal fractures (cases per 1,000 person-years), by branch of service**<br>**Service Branch / Unadjusted IR / aIRR* (95% CI)**<br>Army / 0.48 / 15.45 (7.69-31.05)<br>Marines / 0.37 / 12.65 (6.18-25.89)<br>Navy / 0.20 / 6.44 (2.80-14.83)<br>Air Force / 0.03 / 1.00 (Reference)<br>*IRR adjusted for theatre of operations and year*		78%<br><br>Level of Evidence: III-2
Schoenfeld et al. 2013b [37]<br><br>*Country of origin: United States of America*	Retrospective cohort	U.S. Armed Forces personnel, 1/01/2001 – 31/12/2010	Cases with ICD-9 codes relating to lumbar fracture (i.e. 805.4, 805.5, 806.4, 806.5) were identified in the Defense Medical Epidemiology Database.	**Overall *case* incidence rate for *lumbar* fractures was 0.38 cases per 1,000 person-years**<br>***Case* incidence rate for lumbar fractures (cases per 1,000 person-years), by branch of service**<br>**Service Branch / Unadjusted IR / aIRR* (95% CI)**<br>Army / 0.48 / 1.77 (1.64-1.91)<br>Marines / 0.46 / 1.55 (1.41-1.70)<br>Navy / 0.29 / 1.08 (0.99-1.18)<br>Air Force / 0.28 / 1.00 (Reference)<br><br>*IRR adjusted for age, sex, race, and rank*	***Case* incidence rate for lumbar fractures (cases per 1,000 person-years), by sex**<br>**Sex / Unadjusted IR / aIRR* (95% CI)**<br>Male / 0.40 / 1.19 (1.09-1.31)<br>Female / 0.28 / 1.00 (Reference)<br><br>*IRR adjusted for age, service, race, and rank*<br><br>***Case* incidence rate for lumbar fractures (cases per 1,000 person-years), by race category**<br>**Race Category / Unadjusted IR / aIRR* (95% CI)**<br>White / 0.45 / 2.62 (2.37-2.89)<br>Other / 0.30 / 1.76 (1.55-1.99)	78%<br><br>Level of Evidence: III-2

Study	Study Design	Participants	Methods (Diagnosis / Exposure to Risk Factors)	Occupations or occupational tasks: comparative levels of incidence or prevalence	Other contextual or risk factors	Study Quality Scores #
				***Case* incidence rate for lumbar fractures (cases per 1,000 person-years), by rank category**<br>**Rank** | **Unadjusted IR** | **aIRR* (95% CI)**<br>Junior enlisted | 0.44 | 1.93 (1.65-2.26)<br>Senior enlisted | 0.35 | 1.52 (1.32-1.75)<br>Junior officer | 0.30 | 1.23 (1.04-1.46)<br>Senior officer | 0.30 | 1.00 (Reference)<br>*IRR adjusted for age, sex, race, and service*	Black | 0.18 | 1.00 (Reference)<br>*IRR adjusted for age, sex, service, and rank*<br>***Case* incidence rate for lumbar fractures (cases per 1,000 person-years), by age group**<br>**Age group** | **Unadjusted IR** | **aIRR* (95% CI)**<br>< 20 | 0.36 | 1.10 (0.98-1.24)<br>20-24 | 0.43 | 1.41 (1.21-1.64)<br>25-29 | 0.36 | 1.11 (1.00-1.24)<br>30-34 | 0.34 | 0.94 (0.84-1.05)<br>35-39 | 0.33 | 1.00 (Reference)<br>≥ 40 | 0.38 | 1.24 (1.10-1.40)<br>*IRR adjusted for service, sex, race, and rank*	
Schoenfeld et al. 2012 [38]<br><br>*Country of origin: United States of America*	Retrospective cohort	U.S. Armed Forces personnel, 2000 – 2009	Cases with ICD-9 codes relating to open and closed cervical spine fracture (i.e. 805.0, 805.1) were identified in the Defense Medical Epidemiology Database.	**Overall *case* incidence rate for *cervical* spine fractures was 0.29 cases per 1,000 person-years**<br><br>***Case* incidence rate for cervical fractures (cases per 1,000 person-years), by branch of service**<br>**Service Branch** | **Unadjusted IR** | **aIRR* (95% CI)**<br>Marines | 0.40 | 1.61 (1.45-1.79)<br>Army | 0.32 | 1.45 (1.33-1.59)	***Case* incidence rate for cervical fractures (cases per 1,000 person-years), by sex**<br>**Sex** | **Unadjusted IR** | **aIRR* (95% CI)**<br>Male | 0.31 | 1.45 (1.31-1.61)<br>Female | 0.20 | 1.00 (Reference)<br>*IRR adjusted for age, service, race, and rank*	67%<br><br>Level of Evidence: III-2

Study	Study Design	Participants	Methods (Diagnosis / Exposure to Risk Factors)	Occupations or occupational tasks: comparative levels of incidence or prevalence	Other contextual or risk factors	Study Quality Scores #
				Navy — 0.27 — 1.23 (1.12-1.35)<br>Air Force — 0.22 — 1.00 (Reference)<br>*IRR adjusted for age, sex, race, and rank*<br><br>***Case* incidence rate for cervical fractures (cases per 1,000 person-years), by rank category**<br>**Rank category — Unadjusted IR — aIRR* (95% CI)**<br>Junior enlisted — 0.35 — 1.63 (1.34-1.98)<br>Senior enlisted — 0.27 — 1.42 (1.19-1.70)<br>Junior officer — 0.20 — 1.04 (0.84-1.28)<br>Senior officer — 0.18 — 1.00 (Reference)<br>*IRR adjusted for age, sex, race, and service*	***Case* incidence rate for cervical fractures (cases per 1,000 person-years), by race category**<br>**Race Category — Unadjusted IR — aIRR* (95% CI)**<br>White — 0.31 — 1.21 (1.11-1.32)<br>Other — 0.28 — 1.09 (0.97-1.22)<br>Black — 0.25 — 1.00 (Reference)<br>*IRR adjusted for age, sex, service, and rank*<br><br>***Case* incidence rate for cervical fractures (cases per 1,000 person-years), by age group**<br>**Age group — Unadjusted IR — aIRR* (95% CI)**<br>< 20 — 031 — 1.06 (0.89-1.25)<br>20-24 — 0.35 — 1.25 (1.09-1.43)<br>25-29 — 0.29 — 1.18 (1.04-1.33)<br>30-34 — 0.25 — 1.06 (0.93-1.21)<br>35-39 — 0.23 — 1.00 (Reference)<br>≥ 40 — 0.23 — 1.07 (0.92-1.25)<br>*IRR adjusted for service, sex, race, and rank*	
Schram et al. 2020 [11]	Retrospective cohort	Members of the Australian Regular Army (ARA) and Australian Army Reserves (ARES)	Incidence of serious personal injuries was calculated using data from the Australian Department of Defence's Work Health, Safety, Compensation and	**Fracture incidence (fractures per 1,000 person-years) – fractures classified as serious personal injuries**<br>**Combined — ARA — ARES — IRR (95% CI)**		67%<br><br>Level of Evidence: III-2

Study	Study Design	Participants	Methods (Diagnosis / Exposure to Risk Factors)	Occupations or occupational tasks: comparative levels of incidence or prevalence	Other contextual or risk factors	Study Quality Scores #
Country of origin: Australia			Reporting database (now SENTINEL), for the time-period 1/07/2012 – 30/06/2014.	1.9 1.9 1.7 1.11 (0.54-2.27)		
Shere et al. 2004 [39]<br><br>*Country of origin: United States of America*	Retrospective cohort	U.S. active-duty army soldiers, 1980 – 2000 (N = 4426)	Data from the Total Army Injury and Health Outcomes Database (TAIHOD) were queried to identify ICD-9 codes relating to midfacial and orbital fractures (i.e. 802.4, 802.5, 802.6, 802.7)	**Midfacial and orbital blowout fracture incidence (fractures per 1,000 person-years)**<br><br>| | **Mean IR (across the years 1980-2000)** | **IR range (across the years 1980-2000)** | | **Midfacial** | 0.73 | 0.34-1.00 | | **Orbital** | 0.22 | 0.07-0.61 |	**Mechanism of fracture (percentage of fractures, by body site)**<br><br>| **Mechanism** | **Mid-facial** | **Orbital** | | Violent assault | 28.2% | 37.8% | | Motor vehicle accident | 23.7% | 17.6% | | Athletics | 20.1% | 14.1% | | Falls | 7.1% | 6.7% | | Machinery | 4.2% | 5.7% | | Others | 6.7% | 7.4% | | Unknown | 9.9% | 10.8% |	78%<br><br>Level of Evidence: III-2
Zigras & Dellis, (2018) [40]<br><br>*Country of origin: Greece*	Retrospective cohort	Personnel in the Hellenic Armed Forces (Greece), 4/05/2015 – 4/04/2017 (*n* = 26, 429 recorded parachute jumps)	Local military hospital injury diagnosis data were queried to identify cases of fracture resulting from static line parachute jumps.	**Incidence of fractures (per 1,000 jumps) from static line parachuting**<br><br>| **Officers** | 1.1 | | **Recruits** | 5.3 | | **Overall** | 2.1 |	**Distribution of fracture sites for fractures resulting from static line parachuting**<br><br>| **Anatomical location** | **Percentage of all fractures** | | Ankle | 47.3% | | Fracture-dislocation shoulder | 14.5% | | Shoulder girdle | 10.9% | | Wrist-hand | 7.3% | | Heel | 5.5% | | Metatarsal bones | 5.5% | | Tibia | 3.6% | | Vertebra | 1.8% | | Coccyx | 1.8% | | Femur | 1.8% |	44%<br><br>Level of Evidence: III-2

Methodological quality percentage score is based on the critical appraisal tool specific to the study design, described in the methods section of this review. The levels of evidence are also described in the methods section of this review.

OR: Odds Ratio. Kg: Kilogram. DMED: Defense Medical Epidemiology Database. JTTR: Joint Theater Trauma Registry. RR: Relative Risk. BCT: Basic Combat Training. OEF: Operation Enduring Freedom. OIF: Operation Iraqi Freedom. OND: Operation New Dawn. DMSS: Defense Medical Surveillance System. AFHS: American Hospital Formulatory Service. MSK: Musculoskeletal. IRR: Incidence Rate Ratio. DMED: Defense Medical Epidemiology Database. NZ: New Zealand. NZDF: New Zealand Defence Forces. MOS: Military Operational Specialties. IED: Improved Explosive Device. aIR: Adjusted Incidence Rate. aIRR: Adjusted Incidence Rate Ratio. E1-E9:

Enlisted Rank. O1-O9: Officer Rank. IR: Incidence Rate. ARA: Australian Regular Army. ARES: Australian Army Reserves. TAIHOD: Total Army Injury and Health Outcomes Database. U.S.: United States. ADHD: Attention deficit hyperactivity disorder. STANAG: Standardisation agreement. SD: Standard deviation. ICD: International Classification of Diseases
