# Supplementary material for: The Incidence and Risk Factors for the Development of Fractures in Military Populations: A Systematic Review
Source: Healthcare (Basel). 2026 May 13;14(10):1322. doi: 10.3390/healthcare14101322 (PMC13205265; doi:10.3390/healthcare14101322)
Supplement: Supplementary file 1 [file healthcare-14-01322-s001.zip › Supplementary Material S1 - Note on Protocol.pdf]

Protocol and registration – Amendments

Subsequent to protocol registration, a decision was made to focus the review on military occupations only, rather than paid occupations more broadly, as originally planned. The review reflects this deviation from the registered protocol. The decision to change to a focus solely on military occupations was made for two key reasons:

1. review of initial literature search results indicated that the numbers of articles identified across all paid occupation types was too large to be manageable in this rapid review; and

2. the review was primarily conducted to inform activities relating to military personnel – hence, it was appropriate that evidence relevant to military occupations was the focus, given it was not feasible to consider evidence from all types of paid occupations.

In addition, as the literature search was finally completed in 2024, studies published from 2000 up the time of the search were included, rather than only those published between 2000 and 2020, as originally proposed. The original review commissioned by the funder was also inclusive of both stress and traumatic fractures. Due to restrictions in peer-reviewed journal publication word counts, the decision was made to split the larger review into two systematic reviews: one focussing on stress fractures and the other on traumatic fractures. Finally, given the very large volume of articles identified in the initial literature search, a subsequent decision was made to include only studies that would provide high levels of evidence to answer the review's research questions, relating to fracture aetiology. This meant that cohort studies and other study designs that involved longitudinal follow-up of cohorts (for example, randomised controlled trials and quasi-experimental studies) were included, while case-control studies (except any nested within cohort studies) and cross-sectional studies, which each provide lower levels of evidence, were excluded. The registered protocol for the review was otherwise followed in all respects.
