# Supplementary material for: The Incidence and Risk Factors for the Development of Fractures in Military Populations: A Systematic Review
Source: Healthcare (Basel). 2026 May 13;14(10):1322. doi: 10.3390/healthcare14101322 (PMC13205265; doi:10.3390/healthcare14101322)
Supplement: Supplementary file 1 [file healthcare-14-01322-s001.zip › Supplementary Material S2 - Search Strings.pdf]

SEARCH STRATEGIES

Database	Search Terms
PubMed	(risk[Title/Abstract] OR predict*[Title/Abstract] OR prevalence[Title/Abstract] OR incidence[Title/Abstract] OR caus*[Title/Abstract] OR etiol*[Title/Abstract] OR frequenc*[Title/Abstract] OR rate*[Title/Abstract] OR mediat*[Title/Abstract] OR exposure*[Title/Abstract] OR likelihood[Title/Abstract] OR probability[Title/Abstract] OR factor[Title/Abstract] OR factors[Title/Abstract] OR hazard[Title/Abstract] OR hazards[Title/Abstract] OR predisposing[Title/Abstract]) AND (work*[Title/Abstract] OR occupation*[Title/Abstract] OR profession*[Title/Abstract] OR trade[Title/Abstract] OR employ*[Title/Abstract] OR military[Title/Abstract] OR Defence[Title/Abstract] OR Defense[Title/Abstract] OR airforce[Title/Abstract] OR "air force"[Title/Abstract] OR army[Title/Abstract] OR navy[Title/Abstract] OR recruit[Title/Abstract] OR soldier*[Title/Abstract] OR marines[Title/Abstract] OR "Military Personnel"[Title/Abstract]) AND (Fracture*[Title/Abstract] OR stress fracture*[Title/Abstract] OR overuse fracture*[Title/Abstract] OR bone stress*[Title/Abstract] OR bone strain*[Title/Abstract])
EBSCO<br>(CINAHL and SPORTDiscus)	((TI risk OR AB risk OR TI predict* OR AB predict* OR TI prevalence OR AB prevalence OR TI incidence OR AB incidence OR TI caus* OR AB caus* OR TI etiol* OR AB etiol* OR TI frequenc* OR AB frequenc* OR TI rate* OR AB rate* OR TI mediat* OR AB mediat* OR TI exposure* OR AB exposure* OR TI likelihood OR AB likelihood OR TI probability OR AB probability OR TI factor OR AB factor OR TI factors OR AB factors OR TI hazard OR AB hazard OR TI hazards OR AB hazards OR TI predisposing OR AB predisposing)) AND ((TI work* OR AB work* OR TI occupation* OR AB occupation* OR TI profession* OR AB profession* OR TI trade OR AB trade OR TI employ* OR AB employ* OR TI military OR AB military OR TI Defence OR AB Defence OR TI Defense OR AB Defense OR TI airforce OR AB airforce OR TI "air force" OR AB "air force" OR TI army OR AB army OR TI navy OR AB navy OR TI recruit OR AB recruit OR TI soldier* OR AB soldier* OR TI marines OR AB marines OR TI "Military Personnel" OR AB "Military Personnel"))) AND ((TI Fracture* OR AB Fracture*) OR (TI "stress fracture*" OR AB "stress fracture*") OR (TI "overuse fracture*" OR AB "overuse fracture*") OR (TI "bone stress*" OR AB "bone stress*") OR (TI "bone strain*" OR AB "bone strain*"))
ProQuest	(TI,AB(risk) OR TI,AB(predict*) OR TI,AB(prevalence) OR TI,AB(incidence) OR TI,AB(caus*) OR TI,AB(etiol*) OR TI,AB(frequenc*) OR TI,AB(rate*) OR TI,AB(mediat*) OR TI,AB(exposure*) OR TI,AB(likelihood) OR TI,AB(probability) OR TI,AB(factor) OR TI,AB(factors) OR TI,AB(hazard) OR TI,AB(hazards) OR TI,AB(predisposing)) AND (TI,AB(work*) OR TI,AB(occupation*) OR TI,AB(profession*) OR TI,AB(trade) OR TI,AB(employ*) OR TI,AB(military) OR TI,AB(Defence) OR TI,AB(Defense) OR TI,AB(airforce) OR TI,AB("air force") OR TI,AB(army) OR TI,AB(navy) OR TI,AB(recruit) OR TI,AB(soldier*) OR TI,AB(marines) OR TI,AB("Military Personnel")) AND (TI,AB(Fracture*) OR TI,AB("stress fracture*") OR TI,AB("overuse fracture*") OR TI,AB("bone stress*") OR TI,AB("bone strain*"))
