# Supplementary material for: The Incidence and Risk Factors for the Development of Fractures in Military Populations: A Systematic Review
Source: Healthcare (Basel). 2026 May 13;14(10):1322. doi: 10.3390/healthcare14101322 (PMC13205265; doi:10.3390/healthcare14101322)
Supplement: Supplementary file 1 [file healthcare-14-01322-s001.zip › Supplementary Material S3 - Excluded Articles with Reasons.pdf]

EXCLUDED ARTICLES WITH REASONS

Reason for exclusion	References
Nil incidence/ prevalence/ risk factors or exposures reported	[1-65]
Published prior to 2000	[66-150]
Did not meet required level of evidence (i.e. case-control, cross-sectional design, theses, case series, or hospital-based data)	[151-286]
Duplicate	[287-294]
No access to full text	[295-299]
Not original research or abstract	[300-367]
Unpaid/non-occupational/non-military/elderly/non-human populations	[368-394]
Research protocol or conference abstract	[395-404]
Pre-existing medical condition	[405-428]
Fracture cannot be verified	[429-477]
No valid incidence or risk factor data, or data that can be independently validated	[478]
Articles including only stress fractures	[479-537]

1. Baker, B.S., et al., *Bone, Biomarker, Body Composition, and Performance Responses to 8 Weeks of Reserve Officers' Training Corps Training.* Journal of Athletic Training, 2022. **57**(6): p. 571-580.
2. Barnes, D.R., et al., *Quantifying the Effect of Pelvis Fracture on Lumbar Spine Compression during High-rate Vertical Loading.* Stapp Car Crash J, 2021. **65**: p. 189-216.
3. Chung, B.H., et al., *Consensus and Equipose in the Management of Military Trainee Femoral Neck Stress Fractures: A Survey of Military Surgeons.* Mil Med, 2024. **189**(1-2): p. e82-e89.
4. Coombs, C.V., et al., *Hormonal contraceptive use, bone density and biochemical markers of bone metabolism in British Army recruits.* BMJ Mil Health, 2023. **169**(1): p. 9-16.
5. Fagnant, H.S., et al., *Breakfast Skipping Is Associated with Vitamin D Deficiency among Young Adults entering Initial Military Training.* J Acad Nutr Diet, 2022. **122**(6): p. 1114-1128.e1.
6. Gaffney-Stomberg, E., et al., *Once daily calcium (1000 mg) and vitamin D (1000 IU) supplementation during military training prevents increases in biochemical markers of bone resorption but does not affect tibial microarchitecture in Army recruits.* Bone, 2022. **155**: p. 116269.
7. Gill, N., et al., *Enforcing walking speed and step-length affects joint kinematics and kinetics in male and female healthy adults.* Gait Posture, 2023. **103**: p. 223-228.

8. Hennigar, S.R., et al., *Divergent effects of sex and calcium/vitamin D supplementation on serum magnesium and markers of bone structure and function during initial military training.* Br J Nutr, 2022. **128**(9): p. 1730-1737.
9. Leggit, J.C., et al., *Non-Operative Shoulder Dysfunction in the United States Military.* Mil Med, 2023. **188**(5-6): p. e1003-e1009.
10. Milgrom, C., et al., *Significance of tibial MRI findings of special forces recruits at the onset of their training.* BMJ Mil Health, 2024. **170**(1): p. 9-14.
11. O'Leary, T.J., et al., *The effect of sex and protein supplementation on bone metabolism during a 36-h military field exercise in energy deficit.* J Appl Physiol (1985), 2023. **134**(6): p. 1481-1495.
12. Pietsch, H., et al., *A comparison of fracture response in female and male lumbar spine in simulated under body blast component tests.* J Mech Behav Biomed Mater, 2024. **150**: p. 106303.
13. Rubio, J.E., et al., *Differences in running biomechanics between young, healthy men and women carrying external loads.* Front Bioeng Biotechnol, 2023. **11**: p. 1250937.
14. Shaw, K.A., et al., *Surgically Treated Femoral Neck Stress Fractures Are Likely to Result in Military Separation During Basic Combat Training.* Clin Orthop Relat Res, 2022. **480**(9): p. 1684-1691.
15. Steinmann, N., et al., *Quantification of Behind Shield Blunt Impacts Using a Modified Upper Extremity Anthropomorphic Test Device.* J Biomech Eng, 2022. **144**(9).
16. Sundaramurthy, A., et al., *Effect of stride length on the running biomechanics of healthy women of different statures.* BMC Musculoskelet Disord, 2023. **24**(1): p. 604.
17. Taddei, L., et al., *Effect of blast loading on the risk of rib fractures: a preliminary 3D numerical investigation.* Forensic Sci Int, 2021. **326**: p. 110930.
18. Talbot, M., et al., *Risk assessment of aviators with a total hip arthroplasty.* BMJ Military Health, 2023: p. e002557.
19. Thelen, M., et al., *Demonstration and Evaluation of Physical Examination Techniques Intended to Identify Proximal Femoral Bone Stress Injuries.* Med J (Ft Sam Houst Tex), 2022(Per 22-01/02/03): p. 66-73.
20. Tong, J., et al., *Effects of Stature and Load Carriage on the Running Biomechanics of Healthy Men.* IEEE Trans Biomed Eng, 2023. **70**(8): p. 2445-2453.
21. Beltran, M.J., et al., *Fate of combat nerve injury.* J Orthop Trauma, 2012. **26**(11): p. e198-203.
22. Brown, K.V., et al., *Complications of extremity vascular injuries in conflict.* J Trauma, 2009. **66**(4 Suppl): p. S145-9.
23. Burns, T.C., et al., *Microbiology and injury characteristics in severe open tibia fractures from combat.* J Trauma Acute Care Surg, 2012. **72**(4): p. 1062-7.
24. Caubere, A., et al., *Use of intramedullary nailing in poor sanitary conditions: French Military Medical Service experience.* Orthop Traumatol Surg Res, 2019. **105**(1): p. 173-177.
25. Chalupa, R.L., et al., *Correlation Between Femoral Neck Shaft Angle and Surgical Management in Trainees With Femoral Neck Stress Fractures.* US Army Med Dep J, 2016: p. 1-5.
26. Chun, X., et al., *A cross-sectional study of the effects of load carriage on running characteristics and tibial mechanical stress: implications for stress-fracture injuries in women.* BMC Musculoskeletal Disorders, 2017. **18**: p. 1-12.
27. Claire, E.W.S.L., et al., *Characterization of Lower Extremity Blast Injury.* Military Medicine, 2018. **183**(9-10): p. e448-e453.
28. Cross, J.D., et al., *Return to duty after type III open tibia fracture.* J Orthop Trauma, 2012. **26**(1): p. 43-7.
29. Danelson, K., et al., *Analysis of the Frequency and Mechanism of Injury to Warfighters in the Under-body Blast Environment.* Stapp Car Crash J, 2018. **62**: p. 489-513.

30. de Kruijff, L.G.M., et al., *Combat-related foot injuries: impact on gait and functional outcome.* J R Army Med Corps, 2018. **164**(5): p. 322-327.
31. Dunn, J.C., et al., *US service member tourniquet use on the battlefield: Iraq and Afghanistan 2003–2011.* Trauma, 2016. **18**(3): p. 216-220.
32. Dutton, J., et al., *Clinical value of grading the scintigraphic appearances of tibial stress fractures in military recruits.* Clin Nucl Med, 2002. **27**(1): p. 18-21.
33. Earl-Boehm, J.E., et al., *The effects of military style ruck marching on lower extremity loading and muscular, physiological and perceived exertion in ROTC cadets.* Ergonomics, 2020. **63**(5): p. 629-638.
34. Fox, C.J., et al., *Contemporary management of wartime vascular trauma.* J Vasc Surg, 2005. **41**(4): p. 638-44.
35. Gaffney-Stomberg, E., et al., *Calcium and vitamin D supplementation maintains parathyroid hormone and improves bone density during initial military training: a randomized, double-blind, placebo controlled trial.* Bone, 2014. **68**: p. 46-56.
36. Gaffney-Stomberg, E., et al., *Association Between Single Gene Polymorphisms and Bone Biomarkers and Response to Calcium and Vitamin D Supplementation in Young Adults Undergoing Military Training.* J Bone Miner Res, 2017. **32**(3): p. 498-507.
37. Havenetidis, K., D. Kardaris, and T. Paxinos, *Profiles of musculoskeletal injuries among Greek Army officer cadets during basic combat training.* Mil Med, 2011. **176**(3): p. 297-303.
38. Hoyt, B.W., et al., *Definitive External Fixation for Anterior Stabilization of Combat-related Pelvic Ring Injuries, With or Without Sacroiliac Fixation.* Clin Orthop Relat Res, 2020. **478**(4): p. 779-789.
39. Lake, N., et al., *Improved Functional Outcomes Following Operative Treatment of Midshaft Clavicle Fractures in an Active Duty Population.* Cureus, 2020. **12**(3).
40. Lee, C., et al., *No Difference in Risk of Implant Removal Between Orthogonal Mini-fragment and Single Small-fragment Plating of Midshaft Clavicle Fractures in a Military Population: A Preliminary Study.* Clin Orthop Relat Res, 2020. **478**(4): p. 741-749.
41. Leisey, J., *Prospective validation of the Ottawa Ankle Rules in a deployed military population.* Mil Med, 2004. **169**(10): p. 804-6.
42. Lewandowski, L.R., et al., *Osteomyelitis Risk Factors Related to Combat Trauma Open Femur Fractures: A Case-Control Analysis.* J Orthop Trauma, 2019. **33**(4): p. e110-e119.
43. Loes, G.M.d.K., et al., *Combat-related foot injuries: impact on gait and functional outcome.* Journal of the Royal Army Medical Corps, 2018. **164**(5): p. 322.
44. Lovalekar, M., et al., *Accuracy of recall of musculoskeletal injuries in elite military personnel: a cross-sectional study.* BMJ Open, 2017. **7**(12): p. e017434.
45. Mabry, R.L., et al., *Fatal airway injuries during Operation Enduring Freedom and Operation Iraqi Freedom.* Prehosp Emerg Care, 2010. **14**(2): p. 272-7.
46. May, T., et al., *Accuracy of the Tuning Fork Test for Determination of Presence and Location of Tibial Stress Fractures in a Military Training Population.* Mil Med, 2021. **186**(7-8): p. 733-736.
47. McKay, B.J. and C.A. Bir, *Lower extremity injury criteria for evaluating military vehicle occupant injury in underbelly blast events.* Stapp Car Crash J, 2009. **53**: p. 229-49.
48. Milgrom, C., et al., *Medial tibial stress fracture diagnosis and treatment guidelines.* J Sci Med Sport, 2021. **24**(6): p. 526-530.
49. Motamedi, M.H., S.M. Khatami, and P. Tarighi, *Assessment of severity, causes, and outcomes of hospitalized trauma patients at a major trauma center.* J Trauma, 2009. **66**(2): p. 516-8.
50. Nappo, K.E., et al., *Union Rates and Reported Range of Motion Are Acceptable After Open Forearm Fractures in Military Combatants.* Clin Orthop Relat Res, 2019. **477**(4): p. 813-820.
51. Nunns, M., et al., *Estimated third metatarsal bending stresses are highly susceptible to variations in bone geometry.* Footwear Science, 2017. **9**(3): p. 127-137.

52. Orr, J.D., et al., *Occupational Outcomes and Return to Running Following Internal Fixation of Ankle Fractures in a High-Demand Population.* Foot & Ankle International, 2015. **36**(7): p. 780-786.
53. Pehlivan, O., et al., *Humeral shaft fractures secondary to throwing.* Orthopedics, 2003. **26**(11): p. 1139-41.
54. Petfield, J.L., et al., *Virtual stress testing of fracture stability in soldiers with severely comminuted tibial fractures.* J Orthop Res, 2017. **35**(4): p. 805-811.
55. Plavian, L., *EVALUATION OF STRESS FRACTURE RISK FACTORS FOR RECRUITS.* Papers on Anthropology, 2004. **13**: p. 185-192.
56. Ramasamy, A., et al., *The modern "deck-slap" injury--calcaneal blast fractures from vehicle explosions.* J Trauma, 2011. **71**(6): p. 1694-8.
57. Rice, H., et al., *Altered forefoot function following a military training activity.* Gait Posture, 2019. **74**: p. 182-186.
58. Rice, H.M., et al., *Estimates of Tibial Shock Magnitude in Men and Women at the Start and End of a Military Drill Training Program.* Mil Med, 2018. **183**(9-10): p. e392-e398.
59. Richards, T. and C. Wright, *British Army recruits with low serum vitamin D take longer to recover from stress fractures.* BMJ Mil Health, 2020. **166**(4): p. 240-242.
60. Richardson, P.S., *Dental morbidity in United Kingdom Armed Forces, Iraq 2003.* Mil Med, 2005. **170**(6): p. 536-41.
61. Schechtman, D.W., T.J. Walters, and D.S. Kauvar, *Utility of the Mangled Extremity Severity Score in Predicting Amputation in Military Lower Extremity Arterial Injury.* Ann Vasc Surg, 2021. **70**: p. 95-100.
62. Stewart, S.K., A.P. Pearce, and J.C. Clasper, *Fatal head and neck injuries in military underbody blast casualties.* J R Army Med Corps, 2019. **165**(1): p. 18-21.
63. Xu, C., et al., *A cross-sectional study of the effects of load carriage on running characteristics and tibial mechanical stress: implications for stress-fracture injuries in women.* BMC Musculoskelet Disord, 2017. **18**(1): p. 125.
64. Gaffney-Stomberg, E., et al., *Calcium and vitamin D supplementation and bone health in Marine recruits: Effect of season.* Bone, 2019. **123**: p. 224-233.
65. Barnes, K.R., et al., *The effects of prenatal vitamin supplementation on operationally significant health outcomes in female air force trainees.* Mil Med, 2015. **180**(5): p. 554-8.
66. Almeida, S.A., et al., *Epidemiological patterns of musculoskeletal injuries and physical training.* Med Sci Sports Exerc, 1999. **31**(8): p. 1176-82.
67. Amoroso, P.J., N.S. Bell, and B.H. Jones, *Injury among female and male army parachutists.* Aviat Space Environ Med, 1997. **68**(11): p. 1006-11.
68. Bar-Dayan, Y., Y. Bar-Dayan, and J. Shemer, *Parachuting injuries: a retrospective study of 43,542 military jumps.* Mil Med, 1998. **163**(1): p. 1-2.
69. Bennell, K.L. and P.D. Brukner, *Epidemiology and site specificity of stress fractures.* Clin Sports Med, 1997. **16**(2): p. 179-96.
70. Bijur, P.E., et al., *Comparison of injury during cadet basic training by gender.* Archives of Pediatrics & Adolescent Medicine, 1997. **151**(5): p. 456-61.
71. Cernak, I., et al., *Recognizing, scoring, and predicting blast injuries.* World J Surg, 1999. **23**(1): p. 44-53.
72. Cline, A.D., G.R. Jansen, and C.L. Melby, *Stress fractures in female army recruits: implications of bone density, calcium intake, and exercise.* J Am Coll Nutr, 1998. **17**(2): p. 128-35.
73. Cowan, D.N., et al., *Lower limb morphology and risk of overuse injury among male infantry trainees.* Med Sci Sports Exerc, 1996. **28**(8): p. 945-52.
74. Craig, S.C., et al., *Parachuting injuries during Operation Royal Dragon, Big Drop III, Fort Bragg, North Carolina, May 15/16, 1996.* Mil Med, 1999. **164**(1): p. 41-3.
75. Ekeland, A., *Injuries in military parachuting: a prospective study of 4499 jumps.* Injury, 1997. **28**(3): p. 219-22.

76. Farrow, G.B., *Military static line parachute injuries.* Aust N Z J Surg, 1992. **62**(3): p. 209-14.
77. Finestone, A., et al., *Prevention of stress fractures using custom biomechanical shoe orthoses.* Clinical Orthopaedics & Related Research®, 1999. **360**: p. 182-190.
78. Finestone, A., et al., *Risk factors for stress fractures among Israeli infantry recruits.* Mil Med, 1991. **156**(10): p. 528-30.
79. Friberg, O., *Leg length asymmetry in stress fractures: a clinical and radiological study.* Journal of Sports Medicine & Physical Fitness, 1982. **22**(4): p. 485-488.
80. Friedl, K.E., et al., *Factors associated with stress fracture in young army women: indications for further research.* Mil Med, 1992. **157**(7): p. 334-8.
81. Gant, T.D. and L.I. Epstein, *Low-velocity gunshot wounds to the maxillofacial complex.* J Trauma, 1979. **19**(9): p. 674-7.
82. Garcia, J.E., L.L. Grabhorn, and K.J. Franklin, *Factors associated with stress fractures in military recruits.* Mil Med, 1987. **152**(1): p. 45-8.
83. Giladi, M., et al., *Recurrent stress fractures in military recruits. One-year follow-up of 66 recruits.* J Bone Joint Surg Br, 1986. **68**(3): p. 439-41.
84. Giladi, M., et al., *Stress fractures. Identifiable risk factors.* Am J Sports Med, 1991. **19**(6): p. 647-52.
85. Giladi, M., et al., *External rotation of the hip. A predictor of risk for stress fractures.* Clin Orthop Relat Res, 1987(216): p. 131-4.
86. Gill, R.M. and G.O. Hopkins, *Stress fracture in parachute regiment recruits.* J R Army Med Corps, 1988. **134**(2): p. 91-3.
87. Gordon, N.F., E.P. Hugo, and J.F. Cilliers, *The South African Defence Force physical training programme. Part III. Exertion-related injuries sustained at an SADF basic training centre.* S Afr Med J, 1986. **69**(8): p. 491-4.
88. Gradner Jr, L.I., et al., *Prevention of Lower Extremity Stress Fractures: A Controlled Trial of a Shock Absorbent Insole.* American Journal of Public Health, 1988. **78**(12): p. 1563-1567.
89. Greaney, R.B., et al., *Distribution and natural history of stress fractures in U.S. Marine recruits.* Radiology, 1983. **146**(2): p. 339-46.
90. Grimston, S.K. and R.F. Zernicke, *Exercise-Related Stress Responses in Bone.* Journal of Applied Biomechanics, 1993. **9**(1): p. 2-14.
91. Hansen, M.O., et al., *A prospective evaluation of orthopedic patients evacuated from Operations Desert Shield and Desert Storm: the Walter Reed experience.* Mil Med, 1994. **159**(5): p. 376-80.
92. Hill, P.F., et al., *Stress fracture of the pubic ramus in female recruits.* J Bone Joint Surg Br, 1996. **78**(3): p. 383-6.
93. Hodalić, Z., et al., *Surgical treatment of 1,211 patients at the Vinkovci General Hospital, Vinkovci, Croatia, during the 1991-1992 Serbian offensive in east Slavonia.* Mil Med, 1999. **164**(11): p. 803-8.
94. Hoffman, J.R., et al., *The effect of leg strength on the incidence of lower extremity overuse injuries during military training.* Mil Med, 1999. **164**(2): p. 153-6.
95. Hopson, C.N. and D.R. Perry, *Stress fractures of the calcaneus in women marine recruits.* Clin Orthop Relat Res, 1977(128): p. 159-62.
96. Islinger, R.B., T.R. Kuklo, and D.W. Polly, Jr., *Spine fractures in active duty soldiers and their return to duty rate.* Mil Med, 1998. **163**(8): p. 536-9.
97. Ivanović, A., N. Jović, and S. Vukelić-Marković, *Frontoethmoidal fractures as a result of war injuries.* J Trauma, 1996. **40**(3 Suppl): p. S177-9.
98. Jacob, E., J.M. Erpelding, and K.P. Murphy, *A retrospective analysis of open fractures sustained by U.S. military personnel during Operation Just Cause.* Mil Med, 1992. **157**(10): p. 552-6.
99. Johnson, B.A., T. Neylon, and R. Laroche, *Lesser metatarsal stress fractures.* Clinics in Podiatric Medicine & Surgery, 1999. **16**(4): p. 631-642.

100. Jones, B.H. and J.J. Knapik, *Physical training and exercise-related injuries. Surveillance, research and injury prevention in military populations.* Sports Med, 1999. **27**(2): p. 111-25.
101. Jordaan, G. and M.P. Schwellnus, *The incidence of overuse injuries in military recruits during basic military training.* Mil Med, 1994. **159**(6): p. 421-6.
102. Jovanovic, S., et al., *Wartime amputations.* Mil Med, 1999. **164**(1): p. 44-7.
103. Kazarian, L.E., *Identification and classification of vertebral fractures following emergency capsule egress from military aircraft.* Aviat Space Environ Med, 1978. **49**(1 Pt. 2): p. 150-7.
104. Korzinek, K., *War injuries of the extremities.* Unfallchirurg, 1993. **96**(5): p. 242-7.
105. Kuusela, T.V., *Incidence of bone lesions in the lower extremities during endurance training.* Annals of Clinical Research, 1984. **16**(Suppl. 40): p. 17-19.
106. Lacombe, J., et al., *Epidemiologie des accidents traumatiques en aile planante en milieu militaire. / Epidemiology of taumatic accidents in military hang-gliding.* Journal de Traumatologie du Sport, 1993. **10**(3): p. 170-174.
107. Leedham, C.S., C.G. Blood, and C. Newland, *A descriptive analysis of wounds among U.S. Marines treated at second-echelon facilities in the Kuwaiti theater of operations.* Mil Med, 1993. **158**(8): p. 508-12.
108. Lesho, E.P., *Can tuning forks replace bone scans for identification of tibial stress fractures?* Mil Med, 1997. **162**(12): p. 802-3.
109. Levi, L., et al., *Wartime neurosurgical experience in Lebanon, 1982-85. II: Closed craniocerebral injuries.* Isr J Med Sci, 1990. **26**(10): p. 555-8.
110. Linenger, J.M. and A.F. Shwayhat, *Epidemiology of podiatric injuries in US Marine recruits undergoing basic training.* J Am Podiatr Med Assoc, 1992. **82**(5): p. 269-71.
111. Lowdon, I.M. and M.H. Wetherill, *Parachuting injuries during training descents.* Injury, 1989. **20**(5): p. 257-8.
112. Macleod, M.A., et al., *Incidence of trauma related stress fractures and shin splints in male and female army recruits: retrospective case study.* Bmj, 1999. **318**(7175): p. 29.
113. McCarroll, J.E. and C. Gunderson, *5-year study of incidence rates of hospitalized cases of head injuries in the US Army.* Neuroepidemiology, 1990. **9**(6): p. 296-305.
114. Meurman, K.O., *Stress fracture of the pubic arch in military recruits.* Br J Radiol, 1980. **53**(630): p. 521-4.
115. Milgrom, C., et al., *The effect of pre-induction sports participation on the incidence of stress fractures in Israeli infantry recruits*, in *In The 14th International Jerusalem Symposium on Sports Medicine : program and book of abstracts, Jerusalem, Israel Society of Sports Medicine, 1998, p.20.* 1998: ;.
116. Milgrom, C., et al., *A prospective study of the effect of a shock-absorbing orthotic device on the incidence of stress fractures in military recruits.* Foot Ankle, 1985. **6**(2): p. 101-4.
117. Milgrom, C., et al., *An analysis of the biomechanical mechanism of tibial stress fractures among Israeli infantry recruits. A prospective study.* Clin Orthop Relat Res, 1988(231): p. 216-21.
118. Milgrom, C., et al., *Medial tibial pain. A prospective study of its cause among military recruits.* Clin Orthop Relat Res, 1986(213): p. 167-71.
119. Milgrom, C., et al., *Stress fractures in military recruits. A prospective study showing an unusually high incidence.* J Bone Joint Surg Br, 1985. **67**(5): p. 732-5.
120. Montgomery, L.C., et al., *Orthopedic history and examination in the etiology of overuse injuries.* Med Sci Sports Exerc, 1989. **21**(3): p. 237-43.
121. Murray-Leslie, C.F., D.J. Lintott, and V. Wright, *The knees and ankles in sport and veteran military parachutists.* Ann Rheum Dis, 1977. **36**(4): p. 327-31.
122. Murray-Leslie, C.F., D.J. Lintott, and V. Wright, *The spine in sport and veteran military parachutists.* Ann Rheum Dis, 1977. **36**(4): p. 332-42.
123. Newman, D.G., *The ejection experience of the Royal Australian Air Force: 1951-92.* Aviat Space Environ Med, 1995. **66**(1): p. 45-9.

124. Novak, W.A., *Use of Ottawa ankle decision rules to evaluate blunt ankle trauma case studies by United States Air Force health care providers*. 1999, Uniformed Services University of the Health Sciences. p. 82 p-82 p.
125. Osborne, R.G. and A.A. Cook, *Vertebral fracture after aircraft ejection during Operation Desert Storm.* Aviat Space Environ Med, 1997. **68**(4): p. 337-41.
126. Parsons, T.W., 3rd, et al., *Spine injuries in combat troops--Panama, 1989.* Mil Med, 1993. **158**(7): p. 501-2.
127. Pester, S. and P.C. Smith, *Stress fractures in the lower extremities of soldiers in basic training.* Orthop Rev, 1992. **21**(3): p. 297-303.
128. Pope, R.P., *Prevention of pelvic stress fractures in female army recruits.* Mil Med, 1999. **164**(5): p. 370-3.
129. Pope, R.P., et al., *Predicting attrition in basic military training.* Mil Med, 1999. **164**(10): p. 710-4.
130. Pouilles, J.M., et al., *Femoral bone density in young male adults with stress fractures.* Bone, 1989. **10**(2): p. 105-8.
131. Protzman, R.R., *Physiologic performance of women compared to men. Observations of cadets at the United States Military Academy.* Am J Sports Med, 1979. **7**(3): p. 191-4.
132. Reynolds, K.L., et al., *Cigarette smoking, physical fitness, and injuries in infantry soldiers.* Am J Prev Med, 1994. **10**(3): p. 145-50.
133. Rodden, J.W. and J.W. Simecek, *Dental emergency visits of Marine Corps personnel.* Mil Med, 1995. **160**(11): p. 555-7.
134. Rosin, A., *Impact of the Ottawa Ankle Rules in a U.S. Army troop medical clinic in South Korea.* Military Medicine, 1999. **164**(11): p. 793-4.
135. Ross, J., *A review of lower limb overuse injuries during basic military training. Part 1: Types of overuse injuries.* Mil Med, 1993. **158**(6): p. 410-5.
136. Rudzki, S.J., *Injuries in Australian Army recruits. Part II: Location and cause of injuries seen in recruits.* Mil Med, 1997. **162**(7): p. 477-80.
137. Sahi, T., et al., *Epidemiology, etiology and prevention of stress fractures in the Finnish defence forces and the frontier guard*, in *In, Mann, G. (ed.), Sports injuries : proceedings of the Third Jerusalem Symposium, London, Freund Publishing House Ltd., c1988, p. 113-126.* 1988: ;.
138. Schissel, D.J., *Effort-related chronic compartment syndrome of the lower extremity.* Military Medicine, 1999. **164**(11): p. 830-2.
139. Schwellnus, M.P., G. Jordaan, and T.D. Noakes, *Prevention of common overuse injuries by the use of shock absorbing insoles. A prospective study.* Am J Sports Med, 1990. **18**(6): p. 636-41.
140. Shaffer, R.A., et al., *Use of simple measures of physical activity to predict stress fractures in young men undergoing a rigorous physical training program.* Am J Epidemiol, 1999. **149**(3): p. 236-42.
141. Shanahan, D.F., *Basilar skull fracture in U.S. Army aircraft accidents.* Aviat Space Environ Med, 1983. **54**(7): p. 628-31.
142. Shappell, S.A., *Naval flight deck injuries: a review of Naval Safety Center data, 1977-91.* Aviat Space Environ Med, 1995. **66**(6): p. 590-5.
143. Simchen, E., et al., *Risk factors for infection in fracture war wounds (1973 and 1982 wars, Israel).* Mil Med, 1991. **156**(10): p. 520-7.
144. Simkin, A., et al., *Combined effect of foot arch structure and an orthotic device on stress fractures.* Foot & Ankle International, 1989. **10**(1): p. 25-29.
145. Stoneham, M.D. and N.V. Morgan, *Stress fractures of the hip in Royal Marine recruits under training: a retrospective analysis.* Br J Sports Med, 1991. **25**(3): p. 145-8.
146. Swissa, A., et al., *The effect of pretraining sports activity on the incidence of stress fractures among military recruits. A prospective study.* Clin Orthop Relat Res, 1989(245): p. 256-60.
147. Taimela, S., U.M. Kujala, and K. Osterman, *Stress injury proneness: a prospective study during a physical training program.* Int J Sports Med, 1990. **11**(2): p. 162-5.

148. Truax, A.L., et al., *Incidence and methods of diagnosis of musculoskeletal injuries incurred in Operations Desert Shield and Desert Storm.* Invest Radiol, 1997. **32**(3): p. 169-73.
149. Werner, U., *Ejection associated injuries within the German Air Force from 1981-1997.* Aviat Space Environ Med, 1999. **70**(12): p. 1230-4.
150. Winfield, A.C., et al., *Risk factors associated with stress reactions in female Marines.* Mil Med, 1997. **162**(10): p. 698-702.
151. Alhawas, A., et al., *Completely Displaced Femoral Neck Stress Fracture in a Young Male Soldier With Almost No Functional Impact: A Case Report.* Cureus, 2023. **15**(1): p. e33629.
152. Zalneraitis, B.H., et al., *Relation of Vitamin D Level, BMI, and Location of Lower Extremity Stress Fractures in Military Trainees.* Mil Med, 2022.
153. Min-Yu Tu Col Md, P., et al., *A Rare Case of Rib Fractures During Centrifuge Training.* Military Medicine, 2022. **187**(1-2): p. e242-e245.
154. Parker, W., et al., *Military experience in the management of pelvic fractures from OIF/OEF.* BMJ Mil Health, 2023. **169**(2): p. 108-111.
155. Petfield, J.L., et al., *IDCRP Combat-Related Extremity Wound Infection Research.* Mil Med, 2022. **187**(Suppl 2): p. 25-33.
156. Ran, Y., et al., *Isolated limb fractures - the underestimated injury in the Israeli Defence Forces (IDF).* Injury, 2023. **54**(2): p. 490-496.
157. Schulte, S.S., et al., *Factors associated with return to duty and need for subsequent procedures after calcaneus open reduction internal fixation in the military.* Injury, 2022. **53**(2): p. 771-776.
158. Sommer, F., P.S. Gadjradj, and T. Pippig, *Spinal injuries after ejection seat evacuation in fighter aircraft of the German Armed Forces between 1975 and 2021.* J Neurosurg Spine, 2023. **38**(2): p. 271-278.
159. Tsur, N., et al., *Traumatic maxillofacial injuries: Patterns, outcomes, and long-term follow-up of a military cohort.* Dent Traumatol, 2023. **39**(2): p. 147-156.
160. Yang, J.Z., et al., *Subchondral fatigue fracture of the femoral head in young military recruits: Potential risk factors.* World J Clin Cases, 2023. **11**(28): p. 6733-6743.
161. Bandyopadhyay, K., et al., *Risk factors of osteoporosis in soldiers of the Armed Forces: A cross-sectional study from Western India.* Med J Armed Forces India, 2023. **79**(2): p. 194-200.
162. Gardner, C.L., et al., *A Case-Control Analysis of Bone Stress Injury on Advancement and Health Care Utilization in US Air Force Basic Military Trainees.* Mil Med, 2023. **188**(Suppl 6): p. 690-697.
163. Nesterovica, D., N. Vaivads, and A. Stepens, *Relationship of footwear comfort, selected size, and lower leg overuse injuries among infantry soldiers.* BMC Musculoskelet Disord, 2021. **22**(1): p. 952.
164. Peterson Shian, L., et al., *Military Service Members with Major Lower Extremity Fractures Return to Running with a Passive-dynamic Ankle-foot Orthosis: Comparison with a Normative Population.* Clinical Orthopaedics and Related Research, 2021. **479**(11): p. 2375-2384.
165. Saerah Iffat, Z., et al., *Patterns of Musculoskeletal Injuries Secondary to Strenuous Physical Training, Assessed on Magnetic Resonance Imaging.* Pakistan Armed Forces Medical Journal, 2024. **74**(2): p. 282.
166. Armstrong, D.W., 3rd, et al., *Stress fracture injury in young military men and women.* Bone, 2004. **35**(3): p. 806-16.
167. Beck, T.J., et al., *Stress fracture in military recruits: gender differences in muscle and bone susceptibility factors.* Bone, 2000. **27**(3): p. 437-44.
168. Burgi, A.A., et al., *High serum 25-hydroxyvitamin D is associated with a low incidence of stress fractures.* J Bone Miner Res, 2011. **26**(10): p. 2371-7.
169. Chatzipapas, C.N., et al., *Stress Fractures in Military. Men and Bone duality Related Factors.* International Journal of Sports Medicine, 2008. **29**(11): p. 922-926.

170. Davey, T., et al., *Fundamental differences in axial and appendicular bone density in stress fractured and uninjured Royal Marine recruits--a matched case-control study.* Bone, 2015. **73**: p. 120-6.
171. Dixon, S.J., M.W. Creaby, and A.J. Allsopp, *Comparison of static and dynamic biomechanical measures in military recruits with and without a history of third metatarsal stress fracture.* Clin Biomech (Bristol, Avon), 2006. **21**(4): p. 412-9.
172. Finestone, A., et al., *Overuse injuries in female infantry recruits during low-intensity basic training.* Med Sci Sports Exerc, 2008. **40**(11 Suppl): p. S630-5.
173. Givon, U., et al., *Stress fractures in the Israeli defense forces from 1995 to 1996.* Clin Orthop Relat Res, 2000(373): p. 227-32.
174. Kelly, E.W., *Stress fractures of the pelvis in female Navy recruits: An analysis of possible mechanisms of injury.* Military Medicine, 2000. **165**(2): p. 142-6.
175. Korvala, J., et al., *Genetic predisposition for femoral neck stress fractures in military conscripts.* BMC Genetics, 2010. **11**: p. 95.
176. Kuhn, K.M., et al., *Acetabular retroversion in military recruits with femoral neck stress fractures.* Clin Orthop Relat Res, 2010. **468**(3): p. 846-51.
177. Kupferer, K.R.U.B.S.C., et al., *Femoral Neck Stress Fracture in Air Force Basic Trainees.* Military Medicine, 2014. **179**(1): p. 56-61.
178. Lauder, T.D., et al., *The relation between stress fractures and bone mineral density: evidence from active-duty Army women.* Arch Phys Med Rehabil, 2000. **81**(1): p. 73-9.
179. Nunns, M., et al., *Four biomechanical and anthropometric measures predict tibial stress fracture: a prospective study of 1065 Royal Marines.* Br J Sports Med, 2016. **50**(19): p. 1206-10.
180. Oren Schwartz, M.A.J., et al., *The Association Between History of an Ankle Sprain and Traumatic Meniscal Injury Among Infantry Combat Soldiers in the Israeli Defense Forces: A Historical Cohort Study.* Military Medicine, 2020. **185**(5-6): p. e748-e754.
181. Sanchez-Santos, M.T., et al., *Development of a Prediction Model for Stress Fracture During an Intensive Physical Training Program: The Royal Marines Commandos.* Orthopaedic Journal of Sports Medicine, 2017. **5**(7): p. 1-12.
182. Scheinowitz, M., et al., *Effect of cardiovascular and muscular endurance is not associated with stress fracture incidence in female military recruits: a 12-month follow up study.* J Basic Clin Physiol Pharmacol, 2017. **28**(3): p. 219-224.
183. Schermann, H., et al., *Past Methylphenidate Exposure and Stress Fractures in Combat Soldiers: A Case-Control Study.* Am J Sports Med, 2018. **46**(3): p. 728-733.
184. Strohbach, C.A., et al., *Female recruits sustaining stress fractures during military basic training demonstrate differential concentrations of circulating IGF-I system components: a preliminary study.* Growth Horm IGF Res, 2012. **22**(5): p. 151-7.
185. Yanovich, R., et al., *Bone turnover markers do not predict stress fracture in elite combat recruits.* Clin Orthop Relat Res, 2013. **471**(4): p. 1365-72.
186. Yanovich, R., et al., *Candidate gene analysis in Israeli soldiers with stress fractures.* Journal of Sports Science & Medicine, 2012. **11**(1): p. 147-155.
187. Lauder, T.D., et al., *Sports and physical training injury hospitalizations in the army.* Am J Prev Med, 2000. **18**(3 Suppl): p. 118-28.
188. Amako, M., et al., *Epidemiological patterns of traumatic musculoskeletal injuries and non-traumatic disorders in Japan Self-Defense Forces.* Injury Epidemiology, 2018. **5**(1): p. 1-1.
189. Bergman, B.P. and S.A. Miller, *Equal opportunities, equal risks? Overuse injuries in female military recruits.* J Public Health Med, 2001. **23**(1): p. 35-9.
190. Lovalekar, M., et al., *Using the capture-recapture method to estimate the incidence of musculoskeletal injuries among U.S. Army soldiers.* Journal of Science & Medicine in Sport, 2017. **20**: p. S23-S27.

191. Lovalekar, M., et al., *Epidemiology of musculoskeletal injuries sustained by Naval Special Forces Operators and students.* J Sci Med Sport, 2017. **20 Suppl 4**: p. S51-s56.
192. Oren Schwartz, M.A.J., et al., *Overuse Injuries in the IDF's Combat Training Units: Rates, Types, and Mechanisms of Injury.* Military Medicine, 2018. **183**(3-4): p. e196-e200.
193. Oren Schwartz, M.A.J., et al., *Overuse Injuries Among Female Combat Warriors in the Israeli Defense Forces: A Cross-sectional Study.* Military Medicine, 2018. **183**(11-12): p. e610-e616.
194. Blair, J.A., et al., *Spinal column injuries among Americans in the global war on terrorism.* J Bone Joint Surg Am, 2012. **94**(18): p. e135(1-9).
195. Blair, J.A., et al., *Are spine injuries sustained in battle truly different?* Spine J, 2012. **12**(9): p. 824-9.
196. Blair, J.A., et al., *Military penetrating spine injuries compared with blunt.* Spine J, 2012. **12**(9): p. 762-8.
197. Breeze, J., et al., *Mandibular fractures in British military personnel secondary to blast trauma sustained in Iraq and Afghanistan.* Br J Oral Maxillofac Surg, 2011. **49**(8): p. 607-11.
198. Breeze, J., et al., *Maxillofacial injuries in military personnel treated at the Royal Centre for Defence Medicine June 2001 to December 2007.* Br J Oral Maxillofac Surg, 2010. **48**(8): p. 613-6.
199. Burtis, M.T., et al., *Scaphoid fracture detection in a military population: a standardized approach for medical referral.* Mil Med, 2006. **171**(5): p. 404-8.
200. Carey, T., et al., *Prevalence of radiographic findings consistent with femoroacetabular impingement in military personnel with femoral neck stress fractures.* J Surg Orthop Adv, 2013. **22**(1): p. 54-8.
201. Comstock, S., et al., *Spinal injuries after improvised explosive device incidents: implications for Tactical Combat Casualty Care.* J Trauma, 2011. **71**(5 Suppl 1): p. S413-7.
202. Doucet, J.J., et al., *Combat versus civilian open tibia fractures: the effect of blast mechanism on limb salvage.* J Trauma, 2011. **70**(5): p. 1241-7.
203. Hauret, K.G., et al., *Frequency and causes of nonbattle injuries air evacuated from operations iraqi freedom and enduring freedom, u.s. Army, 2001-2006.* Am J Prev Med, 2010. **38**(1 Suppl): p. S94-107.
204. Hauschild, V.D., et al., *Identification of specific activities associated with fall-related injuries, active component, U.S. Army, 2011.* Msmr, 2016. **23**(6): p. 2-9.
205. Hayton, J., *Reducing Medical Downgrading In A High Readiness Royal Marine Unit.* Journal of the Royal Army Medical Corps, 2004. **150**(3): p. 164.
206. Jensen, A.E., et al., *Prevalence of Musculoskeletal Injuries Sustained During Marine Corps Recruit Training.* Military Medicine, 2019. **184**: p. 511-520.
207. Junge, T., et al., *Outcomes of Talus Fractures Associated With High-Energy Combat Trauma.* Foot Ankle Int, 2017. **38**(12): p. 1357-1361.
208. Kim, K.-E., et al., *Humeral shaft fracture and radial nerve palsy in Korean soldiers: focus on arm wrestling related injury.* BMJ Military Health, 2021. **167**(2): p. 80-83.
209. Lew, T.A., et al., *Characterization of craniomaxillofacial battle injuries sustained by United States service members in the current conflicts of Iraq and Afghanistan.* J Oral Maxillofac Surg, 2010. **68**(1): p. 3-7.
210. Lin, D.L., et al., *Orthopedic injuries during Operation Enduring Freedom.* Mil Med, 2004. **169**(10): p. 807-9.
211. Madson, A.Q., et al., *Non-battle craniomaxillofacial injuries from U.S. military operations.* J Craniomaxillofac Surg, 2013. **41**(8): p. 816-20.
212. Mattila, V.M., et al., *Hospitalisation for injuries among Finnish conscripts in 1990-1999.* Accid Anal Prev, 2006. **38**(1): p. 99-104.
213. Mitchener, T.A. and K.G. Hauret, *Air medical evacuations of soldiers for oral-facial disease and injuries, 2005, Operations Enduring Freedom/Iraqi Freedom.* Mil Med, 2009. **174**(4): p. 376-81.

214. Motamedi, M.H., M. Sagafinia, and M. Famouri-Hosseinizadeh, *Oral and maxillofacial injuries in civilians during training at military garrisons: prevalence and causes.* Oral Surg Oral Med Oral Pathol Oral Radiol, 2012. **114**(1): p. 49-51.
215. Possley, D.R., et al., *The effect of vehicle protection on spine injuries in military conflict.* Spine J, 2012. **12**(9): p. 843-8.
216. Ragel, B.T., et al., *Fractures of the thoracolumbar spine sustained by soldiers in vehicles attacked by improvised explosive devices.* Spine (Phila Pa 1976), 2009. **34**(22): p. 2400-5.
217. Ramasamy, A., et al., *A review of casualties during the Iraqi insurgency 2006--a British field hospital experience.* Injury, 2009. **40**(5): p. 493-7.
218. Roberts, D.C., D.M. Power, and S.A. Stapley, *A review of 10 years of scapula injuries sustained by UK military personnel on operations.* J R Army Med Corps, 2018. **164**(1): p. 30-34.
219. Rohena-Quinquilla, I.R., et al., *Femoral Neck Stress Injuries: Analysis of 156 Cases in a U.S. Military Population and Proposal of a New MRI Classification System.* AJR Am J Roentgenol, 2018. **210**(3): p. 601-607.
220. Schoenfeld, A.J., J.C. Dunn, and P.J. Belmont, *Pelvic, spinal and extremity wounds among combat-specific personnel serving in Iraq and Afghanistan (2003-2011): A new paradigm in military musculoskeletal medicine.* Injury, 2013. **44**(12): p. 1866-70.
221. Schoenfeld, A.J., G.P. Goodman, and P.J. Belmont, Jr., *Characterization of combat-related spinal injuries sustained by a US Army Brigade Combat Team during Operation Iraqi Freedom.* Spine J, 2012. **12**(9): p. 771-6.
222. Taanila, H., et al., *Musculoskeletal disorders in physically active conscripts: a one-year follow-up study in the Finnish Defence Forces.* BMC Musculoskelet Disord, 2009. **10**: p. 89.
223. Webster, C.E., et al., *Environment at the time of injury determines injury patterns in pelvic blast.* J R Army Med Corps, 2019. **165**(1): p. 15-17.
224. Wordsworth, M., et al., *The surgical management of facial trauma in British soldiers during combat operations in Afghanistan.* Injury, 2017. **48**(1): p. 70-74.
225. Zachar, M.R., et al., *Characterization of mandibular fractures incurred from battle injuries in Iraq and Afghanistan from 2001-2010.* J Oral Maxillofac Surg, 2013. **71**(4): p. 734-42.
226. Zakowski, B., I. Wagner, and M. Domzalski, *Analysis of a Military Parachutist Injury - A Retrospective Review of Over 37,000 Landings‡.* Military Medicine, 2019. **184**(1/2): p. e261-e265.
227. Andersen, R.C., et al., *Open, combat-related loss, or disruption of the knee extensor mechanism: treatment strategies, classification, and outcomes.* J Orthop Trauma, 2014. **28**(11): p. e250-7.
228. Ball, V.L., et al., *Traumatic injury patterns associated with static line parachuting.* Wilderness Environ Med, 2014. **25**(1): p. 89-93.
229. Bennett, P.M., et al., *Salvage of Combat Hindfoot Fractures in 2003-2014 UK Military.* Foot Ankle Int, 2017. **38**(7): p. 745-751.
230. Burns, T.C., et al., *Does the zone of injury in combat-related Type III open tibia fractures preclude the use of local soft tissue coverage?* J Orthop Trauma, 2010. **24**(11): p. 697-703.
231. Carmont, M.R., et al., *Sequential metatarsal fatigue fractures secondary to abnormal foot biomechanics.* Mil Med, 2006. **171**(4): p. 292-7.
232. Carow, S.D. and J.D. Houser, *Trainees With Displaced Hip Fractures Present to Physical Therapy With Primary Complaint of Knee Pain.* Mil Med, 2017. **182**(11): p. e2095-e2098.
233. Chalupa, R.L., C. Aberle, and A.E. Johnson, *Observed Rates of Lower Extremity Stress Fractures After Implementation of the Army Physical Readiness Training Program at JBSA Fort Sam Houston.* US Army Med Dep J, 2016: p. 6-9.
234. Cho, R.I., et al., *Concomitant cranial and ocular combat injuries during Operation Iraqi Freedom.* J Trauma, 2009. **67**(3): p. 516-20; discussion 519-20.
235. Clasper, J.C. and S.L. Phillips, *Early failure of external fixation in the management of war injuries.* J R Army Med Corps, 2005. **151**(2): p. 81-6.

236. Comat, G., O. Barbier, and D. Ollat, *The posterior malleolar fracture: a parachute injury not to be overlooked.* Orthop Traumatol Surg Res, 2014. **100**(4): p. 419-22.
237. Commandeur, J., et al., *Identical fracture patterns in combat vehicle blast injuries due to improvised explosive devices; a case series.* BMC Emerg Med, 2012. **12**: p. 12.
238. Covey, D.C., R.B. Lurate, and C.T. Hatton, *Field hospital treatment of blast wounds of the musculoskeletal system during the Yugoslav civil war.* J Orthop Trauma, 2000. **14**(4): p. 278-86; discussion 277.
239. Dong-Kyu, K. and H. Kim Tae, *Femoral neck shaft angle in relation to the location of femoral stress fracture in young military recruits: femoral head versus femoral neck stress fracture.* Skeletal Radiology, 2021. **50**(6): p. 1163-1168.
240. Dunn, J.C., et al., *Radial Head Arthroplasty in the Active Duty Military Service Member With Minimum 2-Year Follow-Up.* J Hand Surg Am, 2017. **42**(8): p. 660.e1-660.e7.
241. Duran-Stanton, A.M. and K.L. Kirk, *"March fractures" on a female military recruit.* Mil Med, 2011. **176**(1): p. 53-5.
242. Finestone, A., et al., *Epidemiology of metatarsal stress fractures versus tibial and femoral stress fractures during elite training.* Foot Ankle Int, 2011. **32**(1): p. 16-20.
243. Fisher, R.A., et al., *Athletic Trainer Integration in US Air Force Basic Training.* Journal of Athletic Training, 2017. **52**(6): p. 1.
244. Formby, P.M., et al., *Outcomes After Operative Management of Combat-Related Low Lumbar Burst Fractures.* Spine (03622436), 2015. **40**(18): p. E1019-E1024.
245. Galvin, J.W., et al., *Infection Rate of Intramedullary Nailing in Closed Fractures of the Femoral Diaphysis After Temporizing External Fixation in an Austere Environment.* Journal of Orthopaedic Trauma, 2015. **29**(9): p. e316-20.
246. Garvin, D., B. Thomson, and C. Mudge, *Predicting femoral neck stress fracture extent with bone scintigraphy.* The Journal of Nuclear Medicine, 2018. **59**: p. 1623.
247. Gordon, W.T., et al., *Outcomes associated with the internal fixation of long-bone fractures proximal to traumatic amputations.* J Bone Joint Surg Am, 2010. **92**(13): p. 2312-8.
248. Greer, M.A., *Incidence of metacarpal fractures in U.S. soldiers stationed in South Korea.* J Hand Ther, 2008. **21**(2): p. 137-41; quiz 142.
249. Griffis, C.E., et al., *Return to Duty in Military Members Following Surgical Treatment of Incomplete Femoral Neck Fractures.* J Surg Orthop Adv, 2018. **27**(4): p. 312-316.
250. Gwinn, D.E., et al., *Blast-induced lower extremity fractures with arterial injury: prevalence and risk factors for amputation after initial limb-preserving treatment.* J Orthop Trauma, 2011. **25**(9): p. 543-8.
251. Hamdi, M., et al., *[Stress fracture locations in military personnel].* Tunis Med, 2007. **85**(2): p. 137-42.
252. Hinsley, D.E., S.L. Phillips, and J.C. Clasper, *Ballistic Fractures During The 2003 Gulf Conflict - Early Prognosis And High Complication Rate.* Journal of the Royal Army Medical Corps, 2006. **152**(2): p. 96.
253. Hoencamp, R., et al., *Challenges in the training of military surgeons: experiences from Dutch combat operations in southern Afghanistan.* European Journal of Trauma & Emergency Surgery, 2014. **40**(4): p. 421-428.
254. Holmgaard, R., et al., *Danish experience with free flaps in war wounds.* Dan Med J, 2016. **63**(1): p. A5180.
255. Inklebarger, J., et al., *Femoral and tibial stress fractures associated with vitamin D insufficiency.* J R Army Med Corps, 2014. **160**(1): p. 61-3.
256. Islinger, R.B., *A review of orthopedic injuries in three recent U.S. military conflicts.* Military Medicine, 2000. **165**(6): p. 463-5.
257. Jacobs, N., et al., *Lower limb injuries caused by improvised explosive devices: proposed 'Bastion classification' and prospective validation.* Injury, 2014. **45**(9): p. 1422-8.

258. John Breeze Lt, C., M. William Gensheimer, and J.J. DuBose Col, *Combat Facial Fractures Sustained During Operation Resolute Support and Operation Freedom's Sentinel in Afghanistan.* Military Medicine, 2020. **185**(9-10): p. 414-416.
259. Lichtenberger, J.P., et al., *Imaging of Combat-Related Thoracic Trauma - Blunt Trauma and Blast Lung Injury.* Mil Med, 2018. **183**(3-4): p. e89-e96.
260. Johnson, J.D., et al., *Return to Duty Following Open Reduction and Internal Fixation of Unstable Ankle Fractures in the Active Duty Population.* Mil Med, 2019. **184**(5-6): p. e381-e384.
261. Joshi, A., et al., *Femoral neck stress fractures in military personnel.* JNMA J Nepal Med Assoc, 2009. **48**(174): p. 99-102.
262. Khan, S.U., et al., *Etiology and pattern of maxillofacial injuries in the Armed Forces of Pakistan.* J Coll Physicians Surg Pak, 2007. **17**(2): p. 94-7.
263. Kumar, A.R., et al., *Lessons from the modern battlefield: successful upper extremity injury reconstruction in the subacute period.* J Trauma, 2009. **67**(4): p. 752-7.
264. Lanier, P.J., et al., *Predictors of Persistent Pain After Fixation of Distal Clavicle Fractures in an Active Military Population.* Orthopedics, 2018. **41**(1): p. e117-e126.
265. Lee, C.H., et al., *Surgical treatment of displaced stress fractures of the femoral neck in military recruits: a report of 42 cases.* Arch Orthop Trauma Surg, 2003. **123**(10): p. 527-33.
266. Lehman, R.A., Jr., et al., *Low lumbar burst fractures: a unique fracture mechanism sustained in our current overseas conflicts.* Spine J, 2012. **12**(9): p. 784-90.
267. Lo, M.C., et al., *High-Mobility Multipurpose Wheeled Vehicle Rollover Accidents and Injuries to U.S. Army Soldiers by Reported Occupant Restraint Use, 1992-2013.* Military Medicine, 2017. **182**(5): p. e1782-e1791.
268. Mody, R.M., et al., *Infectious complications of damage control orthopedics in war trauma.* J Trauma, 2009. **67**(4): p. 758-61.
269. Mossadegh, S., M. Midwinter, and P. Parker, *Developing a cumulative anatomic scoring system for military perineal and pelvic blast injuries.* J R Army Med Corps, 2013. **159 Suppl 1**: p. i40-4.
270. Mossadegh, S., et al., *Improvised explosive device related pelvi-perineal trauma: anatomic injuries and surgical management.* J Trauma Acute Care Surg, 2012. **73**(2 Suppl 1): p. S24-31.
271. Nelson, T.J., et al., *Predictors of mortality in close proximity blast injuries during Operation Iraqi Freedom.* J Am Coll Surg, 2006. **202**(3): p. 418-22.
272. Pavlović, M., et al., *Ejection experience in Serbian Air Force, 1990-2010.* Vojnosanit Pregl, 2014. **71**(6): p. 531-3.
273. Phillips, B.N., D.W. Chun, and M. Colyer, *Closed globe macular injuries after blasts in combat.* Retina, 2013. **33**(2): p. 371-9.
274. Polacek, M. and A. Småbrekke, *Displaced stress fracture of the femoral neck in young active adults.* BMJ Case Rep, 2010. **2010**.
275. Poopitaya, S. and K. Kanchanaroek, *Injuries of the thoracolumbar spine from tertiary blast injury in Thai military personnel during conflict in southern Thailand.* J Med Assoc Thai, 2009. **92 Suppl 1**: p. S129-34.
276. Pope, R.P., *Injury surveillance and systematic investigation identify a rubber matting hazard for anterior cruciate ligament rupture on an obstacle course.* Mil Med, 2002. **167**(4): p. 359-62.
277. Salminen, S.T., et al., *Bilateral femoral fatigue fracture: an unusual fracture in a military recruit.* Clin Orthop Relat Res, 2007. **456**: p. 259-63.
278. Stewart, L., et al., *Combat-Related Extremity Wounds: Injury Factors Predicting Early Onset Infections.* Mil Med, 2019. **184**(Suppl 1): p. 83-91.
279. Thomas, R., et al., *Delay in diagnosis of neck of femur stress fracture in a female military recruit.* J R Nav Med Serv, 2012. **98**(2): p. 27-9.

280. Ucak, M., *Incidence and Severity of Maxillofacial Injuries During the Syrian Civil War in Syrian Soldiers and Civilians.* J Craniofac Surg, 2019. **30**(4): p. 992-995.
281. Ucak, M., *Shrapnel Injuries on Regions of Head and Neck in Syrian War.* J Craniofac Surg, 2020. **31**(5): p. 1191-1195.
282. Williams, T.R., et al., *Acetabular stress fractures in military endurance athletes and recruits: incidence and MRI and scintigraphic findings.* Skeletal Radiol, 2002. **31**(5): p. 277-81.
283. Yanovich, R., et al., *Androgen receptor CAG repeat size is associated with stress fracture risk: a pilot study.* Clin Orthop Relat Res, 2011. **469**(10): p. 2925-31.
284. Clark, H.D., *Military load carriage during prolonged marches on lower extremity mechanics: Influence of gender*. 2013, State University of New York at Buffalo: Ann Arbor. p. 57.
285. Mauntel, T.C., *The influence of lower extremity biomechanics on biochemical markers of bone turnover during Army Cadet Basic Training*. 2016, The University of North Carolina at Chapel Hill: Ann Arbor. p. 174.
286. Trone, D.W., *Assessment of Data Systems, Smoking and Injury, and Poor Training Outcomes in U.S. Military Recruit Populations*. 2011, University of California, San Diego: Ann Arbor. p. 127.
287. CPT, B.H.Z., et al., *Relation of Vitamin D Level, BMI, and Location of Lower Extremity Stress Fractures in Military Trainees.* Military Medicine, 2023. **188**(7-8): p. e1970-e1974.
288. Eckard, T.G., et al., *Association Between Automated Landing Error Scoring System Performance and Bone Stress Injury Risk in Military Trainees.* Journal of Athletic Training, 2021.
289. Kang, L., *CORR Insights®: Proportion of Navy Recruits Diagnosed With Symptomatic Stress Fractures During Training and Monetary Impact of These Injuries.* Clinical Orthopaedics & Related Research®, 2022. **480**(11): p. 2120-2121.
290. Faillace, J., et al., *Scaphoid fracture detection in a military population: a standardized approach for medical referral.* Military Medicine, 2006. **171**(5): p. 404-408.
291. Gam, A., et al., *Comparison of Stress Fractures of Male and Female Recruits during Basic Training in the Israeli Anti-Aircraft Forces.* Military Medicine, 2005. **170**(8): p. 710-2.
292. Schwartz, O., et al., *The Association Between History of an Ankle Sprain and Traumatic Meniscal Injury Among Infantry Combat Soldiers in the Israeli Defense Forces: A Historical Cohort Study.* Mil Med, 2020. **185**(5-6): p. e748-e754.
293. Schaffer, R.A., et al., *Predictors of stress fracture susceptibility in young female recruits.* American Journal of Sports Medicine, 2006. **34**(1): p. 108-115.
294. Trone, D.W., et al., *Factors Associated with Discharge during Marine Corps Basic Training.* Military Medicine, 2007. **172**(9): p. 936-41.
295. Brenner, A.K., *Clinical prediction rule for those soldiers most likely to develop lower extremity stress fractures during initial entry training...2008 Combined Sections Meeting...Nashville, Tennessee, February 6-9, 2008.* Journal of Orthopaedic & Sports Physical Therapy, 2008. **38**(1): p. A76-A76.
296. Kyoung-Eun, K., et al., *Humeral shaft fracture and radial nerve palsy in Korean soldiers: focus on arm wrestling related injury.* BMJ Military Health, 2021. **167**(2): p. 80-83.
297. Ramponi, D.R., V. Hedderick, and S.C. Maloney, *Metatarsal Stress Fractures.* Advanced Emergency Nursing Journal, 2017. **39**(3): p. 168-175.
298. Wang, X., P.S. Wang, and W. Zhou, *Risk factors of military training-related injuries in recruits of Chinese People's Armed Police Forces.* Chin J Traumatol, 2003. **6**(1): p. 12-7.
299. Prouteau, S., C.L. Benhamou, and D. Courteix, *La fracture de fatigue: facteurs de risque et perspectives d'identification. / Stress fracture: Risk factors and identification prospects.* Science & Sports, 2005. **20**(2): p. 59-64.
300. Abbott, A., et al., *Part I: Background and Clinical Considerations for Stress Fractures in Female Military Recruits.* Mil Med, 2023. **188**(1-2): p. 86-92.
301. Abbott, A., et al., *Part II: Risk Factors for Stress Fractures in Female Military Recruits.* Military Medicine, 2023. **188**(1/2): p. 93-99.

302. Barbeau, P., et al., *Musculoskeletal Injuries Among Females in the Military: A Scoping Review.* Mil Med, 2021. **186**(9-10): p. e903-e931.
303. Hamstra-Wright, K.L., E. Djelovic, and J. Payette, *The Relationship Between Stress Fractures and Bone Turnover Markers Is Unclear in Athletic and Military Populations: A Critically Appraised Topic.* International Journal of Athletic Therapy & Training, 2023. **28**(3): p. 144-150.
304. Hughes, J.M., et al., *Promoting adaptive bone formation to prevent stress fractures in military personnel.* Eur J Sport Sci, 2022. **22**(1): p. 4-15.
305. Lovalekar, M., et al., *Musculoskeletal injuries in military personnel-Descriptive epidemiology, risk factor identification, and prevention.* J Sci Med Sport, 2021. **24**(10): p. 963-969.
306. Nindl, B.C. and H. Kyröläinen, *Editorial: Military human performance optimization: Contemporary issues for sustained and improved readiness.* Eur J Sport Sci, 2022. **22**(1): p. 1-3.
307. Potter, B.K., *CORR Insights®: Surgically Treated Femoral Neck Stress Fractures Are Likely to Result in Military Separation During Basic Combat Training.* Clinical Orthopaedics & Related Research®, 2022. **480**(9): p. 1692-1693.
308. Ring, M., et al., *[Stress fractures in the military context].* Unfallchirurgie (Heidelb), 2023. **126**(11): p. 856-862.
309. Shaw, K.A., et al., *Femoral Neck Stress Fractures in Athletes and the Military.* J Bone Joint Surg Am, 2022. **104**(5): p. 473-482.
310. Wardle, S.L., et al., *Feeding female soldiers: Consideration of sex-specific nutrition recommendations to optimise the health and performance of military personnel.* J Sci Med Sport, 2021. **24**(10): p. 995-1001.
311. Greeves, J.P., et al., *Current risks factors and emerging biomarkers for bone stress injuries in military personnel.* J Sci Med Sport, 2023. **26 Suppl 1**: p. S14-s21.
312. Aweid, B., et al., *Stress fractures.* Trauma, 2013. **15**(4): p. 308-321.
313. *Sex-specific considerations in stress fracture risk of military personnel.* Journal of Science and Medicine in Sport, 2017. **20**.
314. Balazs, G.C., et al., *High seas to high explosives: the evolution of calcaneus fracture management in the military.* Mil Med, 2014. **179**(11): p. 1228-35.
315. Balthrop, P.M., J. Nyland, and C.S. Roberts, *Risk factors and musculoskeletal injuries associated with all-terrain vehicle accidents.* J Emerg Med, 2009. **36**(2): p. 121-31.
316. Belmont, P.J., B.D. Owens, and A.J. Schoenfeld, *Musculoskeletal Injuries in Iraq and Afghanistan: Epidemiology and Outcomes Following a Decade of War.* J Am Acad Orthop Surg, 2016. **24**(6): p. 341-8.
317. Beranger, F., et al., *Management of war-related vascular wounds in French role 3 hospital during the Afghan campaign.* Injury, 2017. **48**(9): p. 1906-1910.
318. Boden, B.P. and D.C. Osbahr, *High-risk stress fractures: evaluation and treatment.* J Am Acad Orthop Surg, 2000. **8**(6): p. 344-53.
319. Breeze, J., et al., *Skill sets required for the management of military head, face and neck trauma: a multidisciplinary consensus statement.* J R Army Med Corps, 2018. **164**(2): p. 133-138.
320. Brenner, A., *Stress Fracture Implications within the IET Environment.* Armor, 2007. **116**(6): p. 40-43.
321. Bustos, A.O., et al., *Overuse-Related Injuries of the Musculoskeletal System: Systematic Review and Quantitative Synthesis of Injuries, Locations, Risk Factors and Assessment Techniques.* Sensors, 2021. **21**(7): p. 2438.
322. Clasper, J., *The interaction of projectiles with tissues and the management of ballistic fractures.* J R Army Med Corps, 2001. **147**(1): p. 52-61.
323. Connolly, M., Z.R. Ibrahim, and O.N. Johnson, *Changing paradigms in lower extremity reconstruction in war-related injuries.* Military Medical Research, 2016. **3**.

324. Constantini, N., et al., *RISK FACTORS FOR STRESS FRACTURES AND OTHER ORTHOPEDIC INJURIES IN FEMALE INFANTRY RECRUITS. (Abstract).* Clinical Journal of Sport Medicine, 2004. **14**(6): p. 375-375.
325. DeFroda, S.F., et al., *Bone Stress Injuries in the Military: Diagnosis, Management, and Prevention.* Am J Orthop (Belle Mead NJ), 2017. **46**(4): p. 176-183.
326. Dembowski Maj, S.C., et al., *Injury Surveillance and Reporting for Trainees with Bone Stress Injury: Current Practices and Recommendations.* Military Medicine, 2018. **183**(11-12): p. e455-e461.
327. Dixon, S., et al., *Ankle joint kinematics influence risk of third metatarsal stress fracture in military recruits.* Footwear Science, 2013. **5**(sup1): p. 122.
328. Epstein, Y., et al., *Physiological employment standards IV: integration of women in combat units physiological and medical considerations.* European Journal of Applied Physiology, 2013. **113**(11): p. 2673-2690.
329. Epstein, D., et al., *Injuries associated with the use of ejection seats: a systematic review, meta-analysis and the experience of the Israeli Air Force, 1990-2019.* Injury, 2020. **51**(7): p. 1489-1496.
330. Finestone, A. and C. Milgrom, *How stress fracture incidence was lowered in the Israeli army: a 25-yr struggle.* Med Sci Sports Exerc, 2008. **40**(11 Suppl): p. S623-9.
331. Flinn, S.D., *Changes in stress fracture distribution and current treatment.* Curr Sports Med Rep, 2002. **1**(5): p. 272-7.
332. Friedl, K.E., *Biomedical research on health and performance of military women: accomplishments of the Defense Women's Health Research Program (DWHRP).* J Womens Health (Larchmt), 2005. **14**(9): p. 764-802.
333. Friedl, K.E., R.K. Evans, and D.S. Moran, *Stress fracture and military medical readiness: bridging basic and applied research.* Med Sci Sports Exerc, 2008. **40**(11 Suppl): p. S609-22.
334. Green, N.M. and J.J. Matthews, *The management of acute hip pain in the military: femoral neck stress fractures and tears of the acetabular labrum.* J R Nav Med Serv, 2016. **102**(2): p. 124-9.
335. Hauret, K.G., et al., *Musculoskeletal injuries description of an under-recognized injury problem among military personnel.* Am J Prev Med, 2010. **38**(1 Suppl): p. S61-70.
336. Hosey, R.G.M.D., M.M.F.M.D. Fernandez, and D.L.M.D. Johnson, *Evaluation and Management of Stress Fractures of the Pelvis and Sacrum.* Orthopedics (Online), 2008. **31**(4): p. 383-385.
337. Houston, M.N., et al., *O1 The incidence of ankle injuries in intramural and club sports at the united states military academy.* British Journal of Sports Medicine, 2017. **51**.
338. Jacobs, J.M., K.L. Cameron, and J.A. Bojescul, *Lower extremity stress fractures in the military.* Clin Sports Med, 2014. **33**(4): p. 591-613.
339. Jones, B. and K. Hauret, *The incidence and risk factors for stress fractures and other injuries among U.S. Army trainees.* Journal of Science and Medicine in Sport, 2017. **20**: p. S84-S85.
340. Joy, S.M., *Predicting Lower Extremity Stress Fractures in Young Women Recruited into the Marine Corps. (Abstract).* Clinical Journal of Sport Medicine, 2007. **17**(1): p. 80-81.
341. Kessler, D.F., *Running FASTER: Changing Running Technique to Reduce Stress Injuries.* International Journal of Athletic Therapy & Training, 2020. **25**(2): p. 49-53.
342. Knapik, J.J., *United States Military Parachute Injuries: Part 2: Interventions Reducing Military Parachute Injuries in Training and Operations.* J Spec Oper Med, 2019. **19**(4): p. 109-113.
343. Knapik, J.J. and S.A. Bedno, *Epidemiological Evidence and Possible Mechanisms for the Association Between Cigarette Smoking and Injuries (Part 1).* J Spec Oper Med, 2018. **18**(1): p. 108-112.
344. Knapik, J.J., K. Reynolds, and K.L. Hoedebecke, *Stress Fractures: Etiology, Epidemiology, Diagnosis, Treatment, and Prevention.* J Spec Oper Med, 2017. **17**(2): p. 120-130.
345. Knapik, J.J., K.L. Reynolds, and E. Harman, *Soldier load carriage: historical, physiological, biomechanical, and medical aspects.* Mil Med, 2004. **169**(1): p. 45-56.

346. Martin, N., et al., *Iron status and associations with aerobic performance and stress fracture risk during initial military training.* Journal of Science and Medicine in Sport, 2017. **20**: p. S164-S165.
347. McHale, C., *CORR Insights®: Union Rates and Reported Range of Motion Are Acceptable After Open Forearm Fractures in Military Combatants.* Clin Orthop Relat Res, 2019. **477**(10): p. 2329-2331.
348. Moran, D., R. Evans, and E. Hadad, *Imaging of Lower Extremity Stress Fracture Injuries.* Sports Medicine, 2013. **43**(2): p. 345-356.
349. Moran, D.S., R.K. Evans, and E. Hadad, *Imaging of lower extremity stress fracture injuries.* Sports Med, 2008. **38**(4): p. 345-56.
350. Murray, C.K., et al., *Prevention of infections associated with combat-related extremity injuries.* J Trauma, 2011. **71**(2 Suppl 2): p. S235-57.
351. Nunns, M., et al., *A prospective study identifying risk factors for tibial stress fracture in Royal Marine recruits: initial findings.* Footwear Science, 2013. **5**(sup1): p. 123.
352. Nye, N.S., et al., *Improving Diagnostic Accuracy and Efficiency of Suspected Bone Stress Injuries.* Sports Health, 2016. **8**(3): p. 278-283.
353. Orejel Bustos, A., et al., *Overuse-Related Injuries of the Musculoskeletal System: Systematic Review and Quantitative Synthesis of Injuries, Locations, Risk Factors and Assessment Techniques.* Sensors (Basel), 2021. **21**(7).
354. Orr, R., et al., *Soldier Load Carriage, Injuries, Rehabilitation and Physical Conditioning: An International Approach.* Int J Environ Res Public Health, 2021. **18**(8).
355. Patel, D.S., M. Roth, and N. Kapil, *Stress fractures: diagnosis, treatment, and prevention.* Am Fam Physician, 2011. **83**(1): p. 39-46.
356. Pegrum, J., T. Crisp, and N. Padhiar, *Diagnosis and management of bone stress injuries of the lower limb in athletes.* BMJ : British Medical Journal (Online), 2012. **344**.
357. Peris, P., *Stress fractures.* Best Pract Res Clin Rheumatol, 2003. **17**(6): p. 1043-61.
358. Ramasamy, A., et al., *Blast mines: physics, injury mechanisms and vehicle protection.* J R Army Med Corps, 2009. **155**(4): p. 258-64.
359. Rauh, M.J., et al., *PREDICTORS OF STRESS FRACTURE YOUNG WOMEN RECRUITS. (Abstract).* Journal of Orthopaedic & Sports Physical Therapy, 2005. **35**(1): p. A73-a74.
360. Riley, D.J., *Predictors of fitness test performance in young men*. 2004, University of California, San Diego and San Diego State University: Ann Arbor. p. 107.
361. Rome, K., H.H. Handoll, and R. Ashford, *Interventions for preventing and treating stress fractures and stress reactions of bone of the lower limbs in young adults.* Cochrane Database Syst Rev, 2005. **2005**(2): p. Cd000450.
362. Schaffer, R., *Stress fracture risk varies by race, sex.* Endocrine Today, 2017. **15**(5): p. 7.
363. Schoenfeld, A.J., R.A. Lehman, Jr., and J.R. Hsu, *Evaluation and management of combat-related spinal injuries: a review based on recent experiences.* Spine J, 2012. **12**(9): p. 817-23.
364. Shaffer, S.W. and T.L. Uhl, *Preventing and Treating Lower Extremity Stress Reactions and Fractures in Adults.* Journal of Athletic Training, 2006. **41**(4): p. 466-9.
365. Shenoy, K. and Y.H. Kim, *The Military Medical System and Wartime Injuries to the Spine.* Bull Hosp Jt Dis (2013), 2020. **78**(1): p. 42-45.
366. Weber, J.M., et al., *Calcaneal stress fractures.* Clin Podiatr Med Surg, 2005. **22**(1): p. 45-54.
367. Welch, G.L.M.S., *Risk Factors for Bone Stress Injuries: A Follow-up Study of 102,515 Person-Years.* American Fitness, 2008. **26**(3): p. 27.
368. Alfort, H., K. Johanna Von, and M. Wilcke, *Finger fractures: Epidemiology and treatment based on 21341 fractures from the Swedish Fracture register.* PLoS One, 2023. **18**(7): p. e0288506.
369. Baker, H.P., et al., *A comparison of the incidence of concomitant ipsilateral femoral neck fractures in ballistic versus blunt femur fractures.* Eur J Orthop Surg Traumatol, 2023. **33**(4): p. 843-850.

370. Critchley, M., et al., *Differences in Bone Mineral Density and Associated Factors in Dancers and Other Female Athletes.* Appl Physiol Nutr Metab, 2024.
371. Olson, A., et al., *Low energy gunshot injuries: Does removal of retained bullet fragmentation at the time of internal fixation reduce the risk of fracture related infection?* Injury, 2024. **55**(4): p. 111423.
372. Rice, H., O. Seynnes, and A. Werkhausen, *Effect of increased running speed and weight carriage on peak and cumulative tibial loading.* Scand J Med Sci Sports, 2023. **33**(12): p. 2516-2523.
373. Bass, C.R., et al., *Injury risk in behind armor blunt thoracic trauma.* Int J Occup Saf Ergon, 2006. **12**(4): p. 429-42.
374. Ekenman, I., et al., *The role of biomechanical shoe orthoses in tibial stress fracture prevention.* The American Journal of Sports Medicine, 2002. **30**(6): p. 866-70.
375. Kizaki, K., et al., *Ankle Structures of Professional Soccer (Football) Players With Proximal Diaphyseal Stress Fractures of the Fifth Metatarsal.* J Foot Ankle Surg, 2019. **58**(3): p. 489-491.
376. Saita, Y., et al., *Range limitation in hip internal rotation and fifth metatarsal stress fractures (Jones fracture) in professional football players.* Knee Surg Sports Traumatol Arthrosc, 2018. **26**(7): p. 1943-1949.
377. Al-Hilli, A.B. and D.S. Salih, *Early or delayed surgical treatment in compound limb fractures due to high velocity missile injuries: a 5-year retrospective study from Medical City in Baghdad.* Iowa Orthop J, 2010. **30**: p. 94-8.
378. Bass, E., et al., *Risk-adjusted mortality rates of elderly veterans with hip fractures.* Ann Epidemiol, 2007. **17**(7): p. 514-9.
379. Breeze, J., W. Gensheimer, and J.J. DuBose, *Combat Facial Fractures Sustained During Operation Resolute Support and Operation Freedom's Sentinel in Afghanistan.* Mil Med, 2020. **185**(9-10): p. 414-416.
380. Choi, H.-j. and H.-m. Cho, *Multiple stress fractures of the lower extremity in healthy young men.* Journal of Orthopaedics and Traumatology, 2012. **13**(2): p. 105-110.
381. Dussault, M.C., M. Smith, and I. Hanson, *Evaluation of trauma patterns in blast injuries using multiple correspondence analysis.* Forensic Sci Int, 2016. **267**: p. 66-72.
382. Eardley, W., et al., *Spinal Fractures in Current Military Deployments.* Journal of the Royal Army Medical Corps, 2012. **158**(2): p. 101.
383. He, W., et al., *LOAD CARRIAGE INCREASES MECHANICAL LOADING RATES DURING WALKING.* Conference Proceedings of the Annual Meeting of the American Society of Biomechanics, 2010: p. 920-921.
384. Hughes, J.M., D.C. Dickin, and H. Wang, *The relationships between multiaxial loading history and tibial strains during load carriage.* J Sci Med Sport, 2019. **22**(1): p. 48-53.
385. Ireland, A.W., P.J. Kelly, and R.G. Cumming, *Risk factor profiles for early and delayed mortality after hip fracture: Analyses of linked Australian Department of Veterans' Affairs databases.* Injury, 2015. **46**(6): p. 1028-35.
386. Justin, G.A., et al., *Orbital Fractures and Associated Ocular Injuries in Operation Iraqi Freedom and Operation Enduring Freedom Referred to a Tertiary Care Military Hospital and the Effect on Final Visual Acuity.* Ophthalmic Plast Reconstr Surg, 2020. **36**(1): p. 55-60.
387. Mathieu, L., et al., *Wartime upper extremity injuries: experience from the Kabul International Airport combat support hospital.* Chir Main, 2014. **33**(3): p. 183-8.
388. Milgrom, C., et al., *The effect of muscle fatigue on in vivo tibial strains.* J Biomech, 2007. **40**(4): p. 845-50.
389. Nair, R., A.T. Abdool-Carrim, and J.V. Robbs, *Gunshot injuries of the popliteal artery.* Br J Surg, 2000. **87**(5): p. 602-7.
390. Norozy, A., et al., *Maxillofacial Fracture Patterns in Military Casualties.* J Oral Maxillofac Surg, 2020. **78**(4): p. 611.e1-611.e6.

391. Stern, C.A., et al., *An Analysis of Orthopedic Surgical Procedures Performed During U.S. Combat Operations from 2002 to 2016.* Mil Med, 2019. **184**(11-12): p. 813-819.
392. Wang, H., M. Kia, and D.C. Dickin, *Influences of load carriage and physical activity history on tibia bone strain.* Journal of Sport & Health Science, 2019. **8**(5): p. 478-485.
393. Webster, C.E., et al., *Characterization of Lower Extremity Blast Injury.* Mil Med, 2018. **183**(9-10): p. e448-e453.
394. Weidauer, L.A., et al., *Greater Polar Moment of Inertia at the Tibia in Athletes Who Develop Stress Fractures.* Orthopaedic Journal of Sports Medicine, 2014. **2**(7).
395. Critchley, M., et al., *124 Bone mineral density and associated factors: do young female dancers and other recreational sport athletes differ?* British Journal of Sports Medicine, 2021. **55**(Suppl 1): p. A50-A50.
396. Dallagi, A., et al., *P-226 Military occupational accidents.* Occupational and Environmental Medicine, 2023. **80**(Suppl 1): p. A47-A47.
397. Double, R., et al., *Self-reported Hormonal Contraceptive Use In The British Armed Forces.* Medicine & Science in Sports & Exercise, 2021. **53**(8S): p. 361-361.
398. Greeves, J.P., *Reproductive health and stress fracture risk: a wearables solution.* Journal of Science and Medicine in Sport, 2022. **25**: p. S3.
399. Guerriere, K.I., et al., *Associations Between Physical Fitness Test Scores And Tibial Bone Microarchitecture In Young Adults Entering Military Training.* Medicine & Science in Sports & Exercise, 2021. **53**(8S): p. 127-127.
400. Nindl, B.C., *Role of the insulin-like growth factor system in bone health and military relevance during military training.* Journal of Science and Medicine in Sport, 2022. **25**: p. S3.
401. Popp, K.L., et al., *Prior Physical Activity Influences Changes In Tibial Bone Microarchitecture During U.S. Army Basic Combat Training.* Medicine & Science in Sports & Exercise, 2021. **53**(8S): p. 112-113.
402. Sekel, N.M., et al., *Association Between DXA And HR-pQCT Measurements Of BMD In Active, Recruit-aged Men And Women.* Medicine & Science in Sports & Exercise, 2021. **53**(8S): p. 129-129.
403. Hughes, J.M., et al., *A prospective field study of U.S. Army trainees to identify the physiological bases and key factors influencing musculoskeletal injuries: a study protocol.* BMC Musculoskelet Disord, 2019. **20**(1): p. 282.
404. O'Leary, T.J., *Bone and calcium metabolism markers as an indicator of skeletal adaptation and stress fracture risk in military personnel.* Journal of Science & Medicine in Sport, 2022. **25**: p. S4-S4.
405. Aaron, S.K., et al., *Surgically Treated Femoral Neck Stress Fractures Are Likely to Result in Military Separation During Basic Combat Training.* Clinical Orthopaedics and Related Research, 2022. **480**(9): p. 1684-1691.
406. Alhabeeb, A.Y., et al., *Returning to Work After Traumatic Spine Fractures: Current Status in a Military Hospital.* Mil Med, 2024.
407. Barkley, C., et al., *The Presence of Hip Joint Effusion on MRI Is Predictive of a Grade 4 Femoral Neck Stress Injury.* Mil Med, 2023.
408. Carlson, R.J., Jr., *MRI Predictive Model's Utility in a Recruit Training Environment for Tibia Stress Fractures.* Med J (Ft Sam Houst Tex), 2023(Per 23-4/5/6): p. 10-16.
409. Greenlee, T.A., et al., *Can a Psychologic Profile Predict Successful Return to Full Duty After a Musculoskeletal Injury?* Clin Orthop Relat Res, 2024. **482**(4): p. 617-629.
410. Mueller, C., et al., *Pectoralis Major Tendon Tears During Airborne Operations: Are These Injuries Isolated?* Mil Med, 2022.
411. Perez, K.G., et al., *A Focus on Non-Amputation Combat Extremity Injury: 2001-2018.* Mil Med, 2022. **187**(5-6): p. e638-e643.

412. Rhon, D.I., et al., *Fractures and Chronic Recurrence are Commonly Associated with Ankle Sprains: a 5-year Population-level Cohort of Patients Seen in the U.S. Military Health System.* Int J Sports Phys Ther, 2021. **16**(5): p. 1313-1322.
413. Sinnott, B., et al., *Risk Factors and Consequences of Lower Extremity Fracture Nonunions in Veterans With Spinal Cord Injury.* JBMR Plus, 2022. **6**(3): p. e10595.
414. Vuoncino, M., J. Scheidt, and D.S. Kauvar, *Association between time to revascularization and limb loss in military femoropopliteal arterial injuries.* J Vasc Surg, 2023. **78**(5): p. 1198-1203.
415. Ayan, A. and A. Örsçelik, *Bilateral Tibial Stress Injuries in Recreational Athletes of Army. / Rekreasyonel Asker Sporcularda Bilateral Tibia Stres Yaralanmaları.* Spor Hekimligi Dergisi/Turkish Journal of Sports Medicine, 2020. **55**(1): p. 14-20.
416. Celtikci, E., et al., *Relationship between individual payload weight and spondylolysis incidence in Turkish land forces.* Neurosurg Focus, 2018. **45**(6): p. E12.
417. Cross, A.M., et al., *The incidence of pelvic fractures with traumatic lower limb amputation in modern warfare due to improvised explosive devices.* J R Nav Med Serv, 2014. **100**(2): p. 152-6.
418. Dussault, M.C., I. Hanson, and M.J. Smith, *Blast injury prevalence in skeletal remains: Are there differences between Bosnian war samples and documented combat-related deaths?* Sci Justice, 2017. **57**(6): p. 439-447.
419. Hawkinson, M. and P. Osborn, *Outcomes of Lisfranc Injuries in an Active Duty Military Population.* Foot & Ankle Orthopaedics, 2017. **2**(3).
420. Lee, C.H., et al., *Posttraumatic stress disorder associated with orthopaedic trauma: a study in patients with extremity fractures.* J Orthop Trauma, 2015. **29**(6): p. e198-202.
421. Mbelu, M., et al., *[Frequency of hyperlipasemia in patients with bone fractures on follow up at the Military General Hospital in camp Kokolo].* Pan Afr Med J, 2020. **37**: p. 314.
422. Oh, J.S., et al., *Dismounted Blast Injuries in Patients Treated at a Role 3 Military Hospital in Afghanistan: Patterns of Injury and Mortality.* Mil Med, 2016. **181**(9): p. 1069-74.
423. Penn-Barwell, J.G. and I.D. Sargeant, *Gun-shot injuries in UK military casualties - Features associated with wound severity.* Injury, 2016. **47**(5): p. 1067-71.
424. Petfield, J.L., et al., *Is Bone Loss or Devascularization Associated With Recurrence of Osteomyelitis in Wartime Open Tibia Fractures?* Clin Orthop Relat Res, 2019. **477**(4): p. 789-801.
425. Schoenfeld, A.J., et al., *Characterization of spinal injuries sustained by American service members killed in Iraq and Afghanistan: A study of 2,089 instances of spine trauma.* Journal of Trauma & Acute Care Surgery, 2013. **74**(4): p. 1112-1118.
426. Siddique, M.K. and A.M. Bhatti, *A two-year experience of treating vascular trauma in the extremities in a military hospital.* J Pak Med Assoc, 2013. **63**(3): p. 327-30.
427. Stannard, A., et al., *The epidemiology of noncompressible torso hemorrhage in the wars in Iraq and Afghanistan.* J Trauma Acute Care Surg, 2013. **74**(3): p. 830-4.
428. Nye, N.S., et al., *Evaluating an Algorithm and Clinical Prediction Rule for Diagnosis of Bone Stress Injuries.* Sports Health, 2020. **12**(5): p. 449-455.
429. Alowais, F.A., et al., *Patterns and Characteristics of Intentional Self-inflicted Hand Injuries among Military Personnel: A Retrospective Study and Proposal of Treatment Algorithm.* Plast Reconstr Surg Glob Open, 2022. **10**(11): p. e4648.
430. Garcia, A., et al., *Health Conditions Among Special Operations Forces Versus Conventional Military Service Members: A VA TBI Model Systems Study.* J Head Trauma Rehabil, 2022. **37**(4): p. E292-e298.
431. Gu, W., L.L. Groves, and S.F. McClellan, *Patterns of concomitant traumatic brain injury and ocular trauma in US service members.* Trauma Surg Acute Care Open, 2024. **9**(1): p. e001313.
432. O'Leary, T.J., et al., *Understanding the musculoskeletal injury risk of women in combat: the effect of infantry training and sex on musculoskeletal injury incidence during British Army basic training.* BMJ Mil Health, 2023. **169**(1): p. 57-61.

433. Tsur, N., et al., *A retrospective study of oral pathoses in Israeli military divers and non-divers: 2011-2020.* Dent Traumatol, 2022. **38**(1): p. 48-52.
434. Whittle, R.S., *Distance travelled by military recruits during basic training is a significant risk factor for lower limb overuse injury.* BMJ Mil Health, 2022. **168**(5): p. 343-348.
435. O'Leary, T.J., et al., *Tibial Macrostructure and Microarchitecture Adaptations in Women During 44 Weeks of Arduous Military Training.* J Bone Miner Res, 2021. **36**(7): p. 1300-1315.
436. Ahmed, S.I., et al., *Heterotopic ossification in high-grade open fractures sustained in combat: risk factors and prevalence.* J Orthop Trauma, 2013. **27**(3): p. 162-9.
437. Antikainen, A., et al., *The types and management of dental trauma during military service in Finland.* Dent Traumatol, 2018. **34**(2): p. 87-92.
438. Azevedo, L., et al., *Dental Injuries in a Sample of Portuguese Militaries - A Preliminary Research.* Mil Med, 2018. **183**(11-12): p. e591-e595.
439. Baggaley, M., et al., *Effects of load carriage on biomechanical variables associated with tibial stress fractures in running.* Gait Posture, 2020. **77**: p. 190-194.
440. Banti, M., et al., *Improvised explosive device-related lower genitourinary trauma in current overseas combat operations.* J Trauma Acute Care Surg, 2016. **80**(1): p. 131-4.
441. Bar-Dayan, Y., et al., *Diverse Influences on Parachuting Injuries in Israel.* Journal of the Royal Army Medical Corps, 2000. **146**(2): p. 81.
442. Becker, T., et al., *Incidence of reported dental trauma among soldiers during basic training.* Military Medicine, 2009. **174**(2): p. 190-192.
443. Becker, T. and M. Ashkenazi, *A rubber-covered ceramic weapon reduces the incidence of dental trauma in recruits during combat basic training.* Mil Med, 2011. **176**(10): p. 1117-9.
444. Bennett, P.M., et al., *The management and outcome of open fractures of the femur sustained on the battlefield over a ten-year period.* Bone Joint J, 2015. **97-b**(6): p. 842-6.
445. Dua, A., et al., *Comparison of military and civilian popliteal artery trauma outcomes.* J Vasc Surg, 2014. **59**(6): p. 1628-32.
446. Dutton, J.R., et al., *The Success of Hip Arthroscopy in an Active Duty Population.* Arthroscopy: The Journal of Arthroscopy & Related Surgery, 2016. **32**(11): p. 2251-2258.
447. Evans, R.K., et al., *Effects of a 4-month recruit training program on markers of bone metabolism.* Med Sci Sports Exerc, 2008. **40**(11 Suppl): p. S660-70.
448. Forsberg, J.A., et al., *Heterotopic ossification in high-energy wartime extremity injuries: prevalence and risk factors.* J Bone Joint Surg Am, 2009. **91**(5): p. 1084-91.
449. Fox, C.J., et al., *Popliteal artery repair in massively transfused military trauma casualties: a pursuit to save life and limb.* J Trauma, 2010. **69 Suppl 1**: p. S123-34.
450. Galarneau, M.R., et al., *Traumatic brain injury during Operation Iraqi Freedom: findings from the United States Navy-Marine Corps Combat Trauma Registry.* J Neurosurg, 2008. **108**(5): p. 950-7.
451. Ge, W., J. Mu, and C. Huang, *The GDF5 SNP is associated with meniscus injury and function recovery in male Chinese soldiers.* Int J Sports Med, 2014. **35**(7): p. 625-8.
452. Gifford, S.M., et al., *Effect of temporary shunting on extremity vascular injury: an outcome analysis from the Global War on Terror vascular injury initiative.* J Vasc Surg, 2009. **50**(3): p. 549-55; discussion 555-6.
453. Gray, G.C., et al., *Are Gulf War veterans suffering war-related illnesses? Federal and civilian hospitalizations examined, June 1991 to December 1994.* Am J Epidemiol, 2000. **151**(1): p. 63-71.
454. Hadid, A., et al., *Biomechanical Model for Stress Fracture-related Factors in Athletes and Soldiers.* Med Sci Sports Exerc, 2018. **50**(9): p. 1827-1836.
455. Kok-Yong, S., *Training incidents in armored vehicles in the Singapore Armed Forces.* Military Medicine, 2003. **168**(2): p. 165-71.

456. Mauntel, T.C., et al., *Trunk and Lower Extremity Movement Patterns, Stress Fracture Risk Factors, and Biomarkers of Bone Turnover in Military Trainees.* J Athl Train, 2020. **55**(7): p. 724-732.
457. Muendermann, A., D.J. Stefanyshyn, and B.M. Nigg, *Relationship between footwear comfort of shoe inserts and anthropometric and sensory factors. / Etude du confort de chaussures par rapport aux ajouts (a l ' interieur des chaussures), aux facteursanthropometriques et aux facteurs sensoriels.* Medicine & Science in Sports & Exercise, 2001. **33**(11): p. 1939-1945.
458. Murray, C.K., et al., *Prevention and management of infections associated with combat-related extremity injuries.* J Trauma, 2008. **64**(3 Suppl): p. S239-51.
459. Newman, T., et al., *The effects of prophylactic ankle bracing on dynamic reach distance and obstacle course performance in military cadets.* Mil Med, 2012. **177**(5): p. 567-72.
460. Nunns, M., V. Stiles, and S. Dixon, *The effects of standard issue Royal Marine recruit footwear on risk factors associated with third metatarsal stress fractures.* Footwear Science, 2012. **4**(1): p. 59-70.
461. O'Leary, T.J., et al., *Supplementary Energy Increases Bone Formation during Arduous Military Training.* Med Sci Sports Exerc, 2021. **53**(2): p. 394-403.
462. Patzkowski, J.C., et al., *Multiple associated injuries are common with spine fractures during war.* Spine J, 2012. **12**(9): p. 791-7.
463. Penn-Barwell, J.G., et al., *Acute bilateral leg amputation following combat injury in UK servicemen.* Injury, 2014. **45**(7): p. 1105-10.
464. Schermann, H., et al., *Estimation of Dog-Bite Risk and Related Morbidity Among Personnel Working With Military Dogs.* J Spec Oper Med, 2017. **17**(3): p. 51-54.
465. Seng, K., et al., *Training incidents in armored vehicles in the Singapore Armed Forces.* Military Medicine, 2003. **168**(2): p. 165-171.
466. Sormaala, M.J., et al., *Stress injuries of the calcaneus detected with magnetic resonance imaging in military recruits.* J Bone Joint Surg Am, 2006. **88**(10): p. 2237-42.
467. Stark, D.B., et al., *Human Response and Injury Resulting from Head Impacts with Unmanned Aircraft Systems.* Stapp Car Crash Journal, 2019. **63**: p. 29-64.
468. Vasquez, K.B., et al., *Retrospective Analysis of Injuries in Underbody Blast Events: 2007-2010.* Mil Med, 2018. **183**(suppl_1): p. 347-352.
469. Williams, S., et al., *A comparison of electronic and manual fracture risk assessment tools in screening elderly male US veterans at risk for osteoporosis.* Osteoporosis International, 2017. **28**(11): p. 3107-3111.
470. Yang, J., et al., *Peak BMD assessment in a Chinese infantry recruit group.* Int J Sports Med, 2011. **32**(12): p. 970-4.
471. Young, K.W., et al., *Paratrooper's ankle fracture: posterior malleolar fracture.* Clin Orthop Surg, 2015. **7**(1): p. 15-21.
472. Zadik, Y. and L. Levin, *Orofacial injuries and mouth guard use in elite commando fighters.* Mil Med, 2008. **173**(12): p. 1185-7.
473. Zadik, Y. and L. Levin, *Oral and facial trauma among paratroopers in the Israel Defense Forces.* Dent Traumatol, 2009. **25**(1): p. 100-2.
474. Devlin, J.D., et al., *Incidence of admission to the Physical Training and Rehabilitation Programs in Initial Entry Training during fiscal year 2011.* Military Medicine, 2014. **179**(5): p. 547-552.
475. Little, J.V., et al., *Association of Dynamic Knee Valgus and Bone Stress Injury in US Military Academy Cadets.* J Sport Rehabil, 2023. **32**(7): p. 797-801.
476. Eckard, T.G., et al., *Automated Landing Error Scoring System Performance and the Risk of Bone Stress Injury in Military Trainees.* J Athl Train, 2022. **57**(4): p. 334-340.
477. Koltun, K.J., et al., *Tibial Bone Geometry Is Associated With Bone Stress Injury During Military Training in Men and Women.* Front Physiol, 2022. **13**: p. 803219.

478. Baxter, M.L., C. Baycroft, and G.D. Baxter, *Lower limb injuries in soldiers: feasibility of reduction through implementation of a novel orthotic screening protocol.* Mil Med, 2011. **176**(3): p. 291-6.
479. Bar-Dayan, Y., et al., *Comparison of stress fractures of male and female recruits during basic training in the Israeli anti-aircraft forces.* Military Medicine, 2005. **170**(8): p. 710-712.
480. Constantini, N., et al., *Equipment Modification Is Associated With Fewer Stress Fractures in Female Israel Border Police Recruits.* Military Medicine, 2010. **175**(10): p. 799-804.
481. Carswell, A.T., et al., *Vitamin D Metabolites Are Associated With Musculoskeletal Injury in Young Adults: A Prospective Cohort Study.* J Bone Miner Res, 2023. **38**(10): p. 1453-1464.
482. Cosman, F., et al., *Determinants of stress fracture risk in United States Military Academy cadets.* Bone, 2013. **55**(2): p. 359-66.
483. Cowan, D.N., et al., *Step test performance and risk of stress fractures among female army trainees.* Am J Prev Med, 2012. **42**(6): p. 620-4.
484. Cowan, D.N., et al., *Musculoskeletal injuries among overweight army trainees: incidence and health care utilization.* Occupational Medicine, 2011. **61**(4): p. 247-252.
485. Dash, N. and A.S. Kushwaha, *Stress fractures—a prospective study amongst recruits.* Medical Journal Armed Forces India, 2012. **68**(2): p. 118-122.
486. Davey, T., et al., *Low serum 25-hydroxyvitamin D is associated with increased risk of stress fracture during Royal Marine recruit training.* Osteoporosis International, 2016. **27**(1): p. 171-179.
487. Dixon, S., et al., *Prospective study of biomechanical risk factors for second and third metatarsal stress fractures in military recruits.* J Sci Med Sport, 2019. **22**(2): p. 135-139.
488. Eastman, K., et al., *Distal Tibial Bone Properties and Bone Stress Injury Risk in Young Men Undergoing Arduous Physical Training.* Calcified Tissue International, 2023. **113**(3): p. 317-328.
489. Evans, J.T., et al., *Displaced femoral neck stress fractures in Royal Marine recruits--management and results of operative treatment.* J R Nav Med Serv, 2012. **98**(2): p. 3-5.
490. Finestone, A., et al., *A prospective study of the effect of foot orthoses composition and fabrication on comfort and the incidence of overuse injuries.* Foot & Ankle International, 2004. **25**(7): p. 462-466.
491. Fisher, R., et al., *Outcomes of Embedded Athletic Training Services Within United States Air Force Basic Military Training.* Journal of Athletic Training (Allen Press), 2021. **56**(2): p. 134-140.
492. Griffis, C.E., et al., *Proportion of Navy Recruits Diagnosed With Symptomatic Stress Fractures During Training and Monetary Impact of These Injuries.* Clin Orthop Relat Res, 2022. **480**(11): p. 2111-2119.
493. Hauret, K.G., *The Physical Training and Rehabilitation Program: Duration of rehabilitation and final outcome of injuries in basic combat training.* Military Medicine, 2001. **166**(9): p. 820-6.
494. Heagerty, R., et al., *Retrospective analysis of four-year injury data from the Infantry Training Centre, Catterick.* J R Army Med Corps, 2018. **164**(1): p. 35-40.
495. Hetsroni, I., et al., *The role of foot pronation in the development of femoral and tibial stress fractures: a prospective biomechanical study.* Clinical Journal of Sport Medicine, 2008. **18**(1): p. 18-23.
496. House, C., A. Reece, and D. Roiz de Sa, *Shock-absorbing insoles reduce the incidence of lower limb overuse injuries sustained during Royal Marine training.* Mil Med, 2013. **178**(6): p. 683-9.
497. Hughes, J.M., et al., *Nonsteroidal Anti-Inflammatory Drug Prescriptions Are Associated With Increased Stress Fracture Diagnosis in the US Army Population.* J Bone Miner Res, 2019. **34**(3): p. 429-436.

498. Itskoviz, D., T. Marom, and I. Ostfeld, *Trends of stress fracture prevalence among Israel Defense Forces basic trainees.* Mil Med, 2011. **176**(1): p. 56-9.
499. Kardouni, J.R., et al., *Timing of stress fracture in soldiers during the first 6 career months: a retrospective cohort study.* Journal of Athletic Training, 2021.
500. Kelly, K., et al., *Profiling injuries sustained following implementation of a progressive load carriage program in United States marine corps recruit training.* Work, 2024. **77**(4): p. 1391-1399.
501. Knapik, J., et al., *Stress fracture risk factors in basic combat training.* Int J Sports Med, 2012. **33**(11): p. 940-6.
502. Knapik, J.J., M.A. Sharp, and S.J. Montain, *Association between stress fracture incidence and predicted body fat in United States Army Basic Combat Training recruits.* BMC Musculoskelet Disord, 2018. **19**(1): p. 161.
503. Krauss, M.R., et al., *Excess Stress Fractures, Musculoskeletal Injuries, and Health Care Utilization Among Unfit and Overweight Female Army Trainees.* Am J Sports Med, 2017. **45**(2): p. 311-316.
504. Lappe, J., et al., *Calcium and vitamin d supplementation decreases incidence of stress fractures in female navy recruits.* J Bone Miner Res, 2008. **23**(5): p. 741-9.
505. Lappe, J., et al., *Quantitative ultrasound: use in screening for susceptibility to stress fractures in female army recruits.* J Bone Miner Res, 2005. **20**(4): p. 571-8.
506. Lappe, J.M., M.R. Stegman, and R.R. Recker, *The impact of lifestyle factors on stress fractures in female Army recruits.* Osteoporos Int, 2001. **12**(1): p. 35-42.
507. Mattila, V.M., et al., *Risk factors for bone stress injuries: a follow-up study of 102,515 person-years.* Med Sci Sports Exerc, 2007. **39**(7): p. 1061-6.
508. Merkel, D., et al., *The association between hematological and inflammatory factors and stress fractures among female military recruits.* Med Sci Sports Exerc, 2008. **40**(11 Suppl): p. S691-7.
509. Milgrom, C. and A.S. Finestone, *The effect of stress fracture interventions in a single elite infantry training unit (1983-2015).* Bone, 2017. **103**: p. 125-130.
510. Milgrom, C., et al., *Using bone's adaptation ability to lower the incidence of stress fractures.* The American Journal of Sports Medicine, 2000. **28**(2): p. 245-51.
511. Moran, D.S., et al., *Physical and psychological stressors linked with stress fractures in recruit training.* Scand J Med Sci Sports, 2013. **23**(4): p. 443-50.
512. Moran, D.S., et al., *A simplified model to predict stress fracture in young elite combat recruits.* J Strength Cond Res, 2012. **26**(9): p. 2585-92.
513. Moran, D.S., et al., *Prediction model for stress fracture in young female recruits during basic training.* Med Sci Sports Exerc, 2008. **40**(11 Suppl): p. S636-44.
514. Oetting, A.A., et al., *Non-Cognitive Personality Assessment and Risk of Injuries Among Army Trainees.* Am J Prev Med, 2017. **52**(3): p. 324-330.
515. Orr, R.M., et al., *Models to predict injury, physical fitness failure and attrition in recruit training: a retrospective cohort study.* Mil Med Res, 2020. **7**(1): p. 26.
516. Palmanovich, E., et al., *The effect of army vest design on the occurrence of stress fractures and overuse injuries in female military recruits.* Journal of the Royal Army Medical Corps, 2017. **163**(4): p. 251.
517. Piantanida, N.A., et al., *Injuries during Marine Corps officer basic training.* Mil Med, 2000. **165**(7): p. 515-20.
518. Pihlajamäki, H., et al., *Regular physical exercise before entering military service may protect young adult men from fatigue fractures.* BMC Musculoskelet Disord, 2019. **20**(1): p. 126.
519. Pihlajamäki, H.K., et al., *Long-term outcome of undisplaced fatigue fractures of the femoral neck in young male adults.* Journal of Bone & Joint Surgery, British Volume, 2006. **88B**(12): p. 1574-1579.

520. Pihlajamäki, H.K., et al., *Displaced femoral neck fatigue fractures in military recruits.* J Bone Joint Surg Am, 2006. **88**(9): p. 1989-97.
521. Rauh, M.J., et al., *Epidemiology of stress fracture and lower-extremity overuse injury in female recruits.* Med Sci Sports Exerc, 2006. **38**(9): p. 1571-7.
522. Reis, J.P., et al., *Factors associated with discharge during marine corps basic training.* Mil Med, 2007. **172**(9): p. 936-41.
523. Ross, R.A. and A. Allsopp, *Stress fractures in Royal Marines recruits.* Mil Med, 2002. **167**(7): p. 560-5.
524. Ruohola, J.P., et al., *Association between serum 25(OH)D concentrations and bone stress fractures in Finnish young men.* J Bone Miner Res, 2006. **21**(9): p. 1483-8.
525. Ruohola, J.P., et al., *Can elevated serum TRACP-5b levels predict stress fractures? A cohort study.* Scand J Surg, 2009. **98**(4): p. 239-43.
526. Salminen, S.T., et al., *Displaced fatigue fractures of the femoral shaft.* Clin Orthop Relat Res, 2003(409): p. 250-9.
527. Shapiro, M., K. Zubkov, and R. Landau, *Diagnosis of Stress fractures in military trainees: a large-scale cohort.* BMJ Mil Health, 2022. **168**(5): p. 382-385.
528. Sharma, J., et al., *Musculoskeletal injuries in British Army recruits: a prospective study of diagnosis-specific incidence and rehabilitation times.* BMC Musculoskelet Disord, 2015. **16**: p. 106.
529. Sheehan, K.M., et al., *The response of a bone resorption marker to Marine recruit training.* Military Medicine, 2003. **168**(10): p. 797-801.
530. Sormaala, M.J., et al., *Bone stress injuries of the talus in military recruits.* Bone, 2006. **39**(1): p. 199-204.
531. Välimäki, V.V., et al., *Risk factors for clinical stress fractures in male military recruits: a prospective cohort study.* Bone, 2005. **37**(2): p. 267-73.
532. Wood, A.M., et al., *Incidence and Time to Return to Training for Stress Fractures during Military Basic Training.* Journal of Sports Medicine, 2014. **2014**: p. 282980.
533. Wood, P.S. and P.E. Krüger, *Flexibility as risk factor for stress-fracture development in South African male soldiers.* South African Family Practice, 2015. **57**(4): p. 235-240.
534. Yanovich, R., et al., *Anemia, iron deficiency, and stress fractures in female combatants during 16 months.* J Strength Cond Res, 2011. **25**(12): p. 3412-21.
535. Zhao, L., et al., *Prospective cohort study of the risk factors for stress fractures in Chinese male infantry recruits.* J Int Med Res, 2016. **44**(4): p. 787-95.
536. Bulathsinhala, L., et al., *Risk of Stress Fracture Varies by Race/Ethnic Origin in a Cohort Study of 1.3 Million US Army Soldiers.* J Bone Miner Res, 2017. **32**(7): p. 1546-1553.
537. Fedgo, A.A. and S. Stahlman, *Increased risk for stress fractures and delayed healing with NSAID receipt, U.S. Armed Forces, 2014-2018.* Msmr, 2020. **27**(2): p. 18-25.
538. Finestone, A.S., et al., *Evaluation of the performance of females as light infantry soldiers.* Biomed Res Int, 2014. **2014**: p. 572953.
539. Johnson, A.S., et al., *Incidence and Risk Factors for Bone Stress Injuries in United States Air Force Special Warfare Trainees.* Mil Med, 2024.
540. MacGregor, A.J., et al., *Sex Differences in the Incidence and Risk of Ankle-Foot Complex Stress Fractures Among U.S. Military Personnel.* J Womens Health (Larchmt), 2022. **31**(4): p. 586-592.
541. Waterman, B.R., et al., *Epidemiology of Lower Extremity Stress Fractures in the United States Military.* Mil Med, 2016. **181**(10): p. 1308-1313.
542. Schaffer, R.A., et al., *Predictors of stress fracture susceptibility in young female recruits.* American Journal of Sports Medicine, 2006. **34**(1): p. 108-115.
