# Supplementary material for: The Incidence and Risk Factors for the Development of Fractures in Military Populations: A Systematic Review
Source: Healthcare (Basel). 2026 May 13;14(10):1322. doi: 10.3390/healthcare14101322 (PMC13205265; doi:10.3390/healthcare14101322)
Supplement: Supplementary file 1 [file healthcare-14-01322-s001.zip › Supplementary Material S4 - Data Extraction Table (recruits).pdf]

Characteristics and key findings of studies of traumatic fractures in military recruit/trainee populations

Study	Study Design	Participants	Methods (Diagnosis / Exposure to Risk Factors)	Occupations or occupational tasks: comparative levels of incidence or prevalence	Other contextual or risk factors	Study Quality Scores #
Claassen, Hu & Rohrbeck (2014) [10]<br><br>*Country of origin: United States of America*	Retrospective cohort	Active U.S. military service members (Army, Navy, Air Force, Marine Corps, Coast Guard), recruits, and members deployed to OEF, OIF or OND. Surveillance period for active service (except Coast Guard) and recruit cohorts was 1/01/2003 – 31/12/2012; deployed personnel surveillance was 1/01/2008 – 31/12/2012. Coast guard periods were 2007 – 2012.<br>Based on case number and case incidence rate data reported for all fractures in recruits, we calculated an estimate of the total person-years of exposure of recruits to recruit training to be 284,439 person-years.	Data from the Defense Medical Surveillance System, Theater Medical Data Store, and Transportation Command Regulating and Command and Control Evacuation System were queried for incident ICD-9 codes for fracture types: stress, pathologic, head, vertebra, ribs, sternum, larynx and trachea, pelvis, arm, hand, leg, foot/ankle, unspecified.<br><br>Fracture and exposure data presented here exclude data for active service members and deployed personnel and relate only to recruits.	**Overall incidence rate of traumatic/frank fractures in *recruits* from 2003 – 2012, excluding** stress fractures and pathologic fractures (the latter assumed to be stress fractures in nearly all cases), **was 21.8 fractures per 1,000 person-years**<br><br>**Incidence rates (fractures per 1,000 person years) of traumatic/frank fractures, excluding stress fractures and pathologic fractures, in recruits from the U.S. Armed Forces, by anatomical location**<br>**Anatomical location — IR**<br>Foot/ankle — 8.1<br>Hand — 6.1<br>Leg — 2.6<br>Arm — 1.6<br>Head — 1<br>Ribs — 0.7<br>Vertebra — 0.5<br>Pelvis — 0.4<br>Shoulder — 0.4<br>Unspecified — 0.3	**Estimated incidence rates (fractures per 1,000 person-years) of traumatic/frank fractures (excluding stress fractures and pathologic fractures) in recruits, by sex – derived from data reported in the study and assuming similar IR for pathologic fractures in both sexes**<br>**Sex — IR**<br>Female — 30.0<br>Male — 20.3<br>Female: male IRR for traumatic/frank fractures in recruits: 1.48	67%<br><br>Level of Evidence: III-2
Kucera et al. 2016 [73]<br><br>*Country of origin: United States of America*	Prospective cohort	Military academy cadets or midshipmen from USMA (Westpoint), USNA, and USAFA, totalling three classes from each between 2005 and 2008 (N = 9,811; *male* N = 7,390, age range = 16 – 23 years, mean age = 18.8 years; *female* N = 2,421, age range = 16 – 22, mean age was not available to be computed).	Lower extremity injuries, including fractures occurring during two months of basic training (1 Jul-31 Aug) were identified via queries of the Defense Medical Surveillance System (DMSS) using ICD-9 codes.	**Frequencies and calculated incidence rates (fractures per 1,000 person-years) of lower extremity traumatic *fractures* among first-year cadets during 2 months of basic training**<br>**Location — Fracture (n; IR)**<br>Hip — 3; 1.83<br>Upper leg/thigh — 6; 3.67<br>Knee — 0; 0<br>Lower leg/ankle — 24; 14.68<br>Foot/toes — 26; 15.90<br>Other/unspecified lower extremity — 1; 0.61<br>Total (lower extremity sites) — 60; 36.69		78%<br><br>Level of Evidence: II

Study	Study Design	Participants	Methods (Diagnosis / Exposure to Risk Factors)	Occupations or occupational tasks: comparative levels of incidence or prevalence	Other contextual or risk factors	Study Quality Scores [#]
Montain et al. 2013 [24]<br><br>*Country of origin: United States of America*	Retrospective cohort	All U.S. Army Basic Combat Training (BCT) trainees from Jan 1997 – Jan 2007 (*n* = 421,461 men; *n* = 90,141 women).	The Armed Forces Health Surveillance Center (AFHSC) data repository was utilised to extract personnel demographics, characteristics, and medical encounters data. Medical encounters were queried using ICD-9 codes relating to lower leg fractures (820-829) and frank fractures (800-829).<br>Outcome measures of interest were personnel primary Home of Record, fracture occurrence, exposure to ultraviolet (UV) light, and relationship between such exposure and fracture occurrence.	Overall *case* incidence rate for *lower limb* frank fractures during army basic training over the period of observation was 7.7 cases per 1,000 person-years<br><br>Overall *case* incidence rate for all *frank* fractures (including lower limb frank fractures) during army basic training over the period of observation was 12.2 cases per 1,000 person-years	***Case* incidence rates (cases per 1,000 person-years) and odds ratios (OR) for lower limb (LL) and frank fractures (FF) comparing risks for men and women trainees**<br><br>**Men IR** / **Women IR** / **OR (95% CI) women/men**<br>LL = 6.9 / LL = 11.5 / LL = 1.68 (1.56-1.80)<br>FF = 11.1 / FF = 17.5 / FF = 1.59 (1.51-1.69)<br><br>Odds ratio comparing risks for personnel from low and high UV index groups of sustaining a lower limb fracture: 0.95 (95% CI 0.87-1.02; NS)<br><br>**Odds ratios (OR) comparing risks of sustaining *lower limb* fractures during BCT between HOR UV index levels or race/ethnicity category, stratified by sex**<br><br>**UV Index** / **Men (OR [95% CI])** / **Women (OR [95% CI])**<br>High / 1.00 (reference) / 1.00 (reference)<br>Medium / 1.02 (0.94-1.12) / 1.05 (0.91-1.21)<br>Low / 0.98 (0.89-1.07) / 0.93 (0.80-1.09)<br>**Race/ ethnicity** / **Men (OR [95% CI])** / **Women (OR [95% CI])**<br>White / 2.01 (1.77-2.29) / 1.84 (1.57-2.15)<br>Black / 1.00 (reference) / 1.00 (reference)<br>Hispanic / 1.74 (1.48-2.06) / 1.44 (1.15-1.79)<br>Asian / 1.44 (1.11-1.86) / 1.40 (0.96-2.05)<br>Am. Indian / 1.68 (1.14-2.49) / 2.00 (1.30-3.08)	78%<br><br>Level of Evidence: III-2

Study	Study Design	Participants	Methods (Diagnosis / Exposure to Risk Factors)	Occupations or occupational tasks: comparative levels of incidence or prevalence	Other contextual or risk factors	Study Quality Scores #
					(see below)	

Other	2.88 (1.64-5.04)	1.58 (0.58-4.27)
Unknown	1.68 (1.07-2.66)	0.66 (0.25-1.78)

Odds ratios (OR) comparing risks of sustaining *lower limb* fractures during BCT between HOR UV index levels and stratified by racial group/ethnicity and sex

Race / Ethnicity	UV Index	Men (OR [95% CI])
White	High	1.00 (reference)
	Medium	0.94 (0.85-1.05)
	Low	0.85 (0.76-0.94)
Black	High	1.00 (reference)
	Medium	1.33 (0.98-1.80)
	Low	1.48 (1.05-2.09)
Hispanic	High	1.00 (reference)
	Medium	1.26 (0.96-1.67)
	Low	1.11 (0.84-1.47)
Asian	High	1.00 (reference)
	Medium	0.72 (0.40-1.29)
	Low	1.19 (0.70-2.00)
Am. Indian	High	1.00 (reference)

Study	Study Design	Participants	Methods (Diagnosis / Exposure to Risk Factors)	Occupations or occupational tasks: comparative levels of incidence or prevalence	Other contextual or risk factors			Study Quality Scores #
						Medium	1.13 (0.50-2.59)	
						Low	0.45 (0.15-1.31)	
					**Race / Ethnicity**	**UV Index**	**Women (OR [95% CI])**	
					White	High	1.00 (reference)	
						Medium	0.94 (0.78-1.13)	
						Low	0.80 (0.65-0.97)	
					Black	High	1.00 (reference)	
						Medium	1.15 (0.85-1.56)	
						Low	1.01 (0.68-1.50)	
					Hispanic	High	1.00 (reference)	
						Medium	1.52 (1.01-2.28)	
						Low	0.98 (0.61-1.57)	
					Asian	High	1.00 (reference)	
						Medium	0.92 (0.41-2.05)	
						Low	0.70 (0.27-1.81)	
					Am. Indian	High	1.00 (reference)	
						Medium	1.00 (0.41-2.48)	
						Low	0.35 (0.10-1.25)	

Study	Study Design	Participants	Methods (Diagnosis / Exposure to Risk Factors)	Occupations or occupational tasks: comparative levels of incidence or prevalence	Other contextual or risk factors	Study Quality Scores #
Popovich et al. 2000 [47]<br><br>*Country of origin: United States of America*	Quasi-experimental	Six companies of U.S. Army recruits undergoing basic training (8 weeks) from July, August and September 1989 (N = 1357).	Medical records were reviewed from the William Beaumont Army Medical Center utilising customised forms designed for the study. Injury diagnoses were confirmed via bone scans and x-rays where necessary.<br>Company interventions were as follows: C1 and C2 were standard controls, R2, R3, and R4 were training companies which utilised rest from running during week 2, 3, or 4, respectively. R5 increased running volumes; however, withdrew during week 4 and did not resume until week 6 due to an early impression of higher injury rates.	Overall incidence of traumatic fractures in U.S. Army recruits undergoing basic training was 16.2 fractures per 1,000 recruits over the 8-week period of observation, equating to 105.3 fractures per 1,000 person-years.<br>Overall incidence of traumatic *lower extremity* fractures in U.S. Army recruits undergoing basic training was 14.0 fractures per 1,000 recruits over the 8-week period of observation, equating to 91.0 fractures per 1,000 person-years.<br><br>Overall total incidence of traumatic *upper extremity* fractures in U.S. Army recruits undergoing basic training was 2.2 fractures per 1,000 recruits over the 8-week period of observation, equating to 14.3 fractures per 1,000 person-years.		89%<br><br>Level of Evidence: III-2

Methodological quality percentage score is based on the critical appraisal tool specific to the study design, described in the methods section of this review. The levels of evidence are also described in the methods section of this review.

OEF: Operation Enduring Freedom. OIF: Operation Iraqi Freedom. OND: Operation New Dawn. OR: Odds Ratio. IDF: Israel Defense Forces. IR: Incidence Rate. U.S.: United States. BCT: Basic Combat Training. AFHSC: Armed Forces Health Surveillance Center. FF: Frank Fracture. LF: Lisfranc Fracture. HOR: Home of Residence. UV: Ultraviolet. USMA: United States Military Academy. USNA: United States Naval Academy. USAFA: United States Air Force Academy.
